# Supplementary material for: Transfer of IgG from long COVID patients induces symptomology in mice
Source: Cell Rep Med. 2026 Mar 24;7(4):102693. doi: 10.1016/j.xcrm.2026.102693 (PMC13130648; doi:10.1016/j.xcrm.2026.102693)
Supplement: Document S1. Figures S1–S10 and Tables S1, S2, S4, and S6 [file mmc1.pdf]

Cell Reports Medicine, Volume 7

Supplemental information

Transfer of IgG from long COVID patients induces symptomology in mice

Hung-Jen Chen, Brent Appelman, Hanneke L.D.M. Willemen, Amelie Bos, Judith Prado, W. Ashwin Mak, Noa Keijzer, Patrícia Silva Santos Ribeiro, Sara Vieira Goncalves, Sabine Versteeg, Chiara.E. Geyer, Mads Larsen, Eline Schüchner, Marije K. Bomers, Ayesha H.A. Lavell, Amsterdam UMC COVID-19 biobank, Braeden Charlton, Rob Wüst, W. Joost Wiersinga, Michèle van Vugt, Gestur Vidarsson, Niels Eijkelkamp, and Jeroen den Dunnen

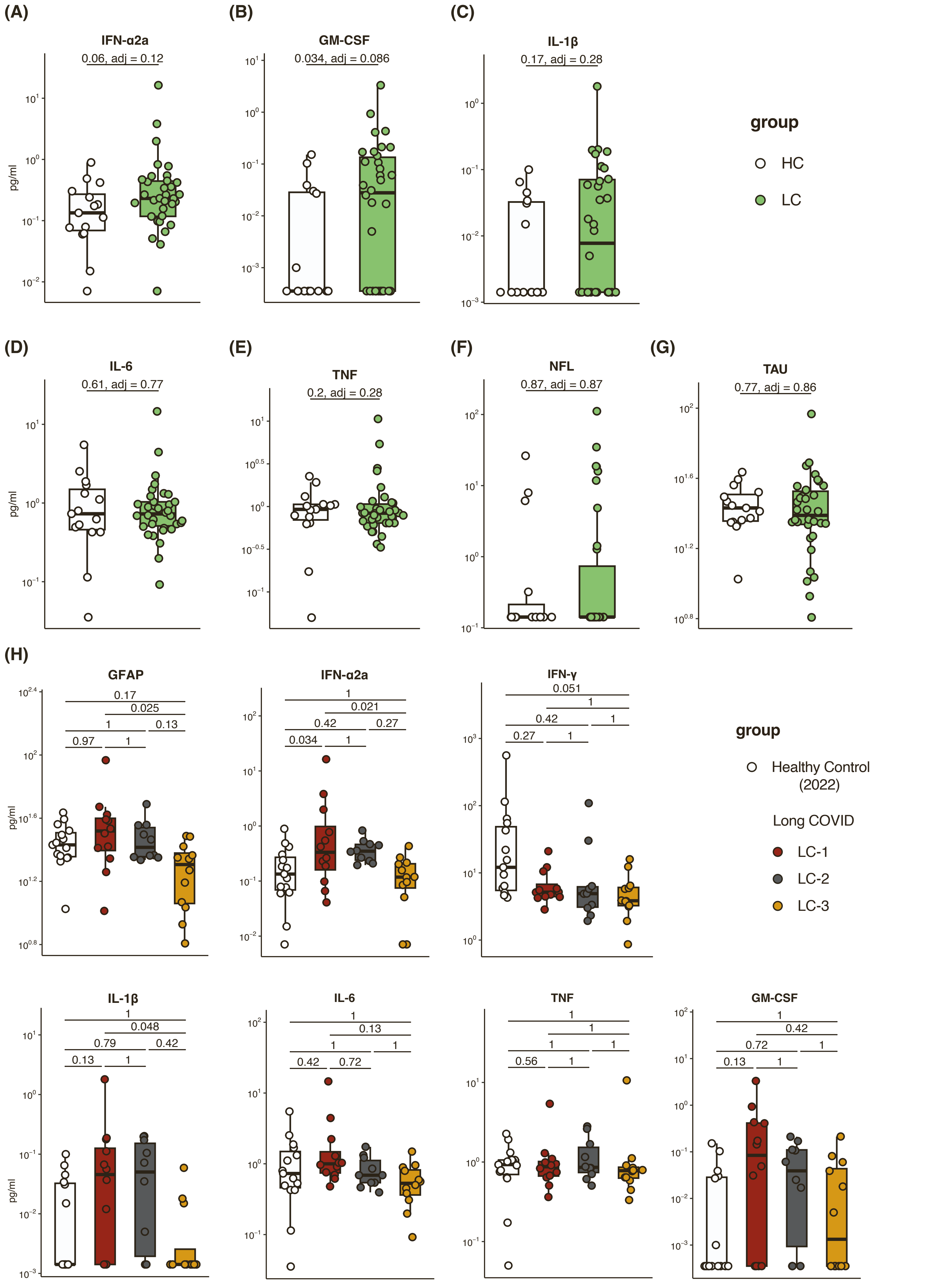

(A)
IFN-α2a
0.06, adj = 0.12
pg/ml
(B)
GM-CSF
0.034, adj = 0.086
(C)
IL-1β
0.17, adj = 0.28
group
HC
LC
(D)
IL-6
0.61, adj = 0.77
(E)
TNF
0.2, adj = 0.28
(F)
NFL
0.87, adj = 0.87
(G)
TAU
0.77, adj = 0.86
(H)
GFAP
0.17
0.025
1
0.13
0.97
1
IFN-α2a
1
0.021
0.42
0.27
0.034
1
IFN-γ
0.051
1
0.42
1
0.27
1
group
Healthy Control (2022)
Long COVID
LC-1
LC-2
LC-3
IL-1β
1
0.048
0.79
0.42
0.13
1
IL-6
1
0.13
1
1
0.42
0.72
TNF
1
1
1
1
0.56
1
GM-CSF
1
0.42
0.72
1
0.13
1

Figure S1: Plasma biomarkers of Long COVID compared to healthy SARS-CoV-2 recovered participants. Targeted quantitative measurements of cytokines and neuro-injury markers of (A-G) 34 Long COVID patients (LC) and 15 healthy controls (HC) with prior SARS-CoV-2 infection using MSD. (H) Subgroup comparisons. Boxes show median and IQR; points are individuals. Numbers above brackets are p values with BH-adjusted p values in parentheses from linear models with group/endotype as the main effect and age, sex, and days-since-infection as covariates; pairwise contrasts are BH-corrected unless stated. All values are shown in pg/ml on a logarithmic y-axis Shown is the interquartile range and the median. ns, not significant. Abbreviations: HC, healthy control; LC, Long COVID; NFL, neurofilament light chain.

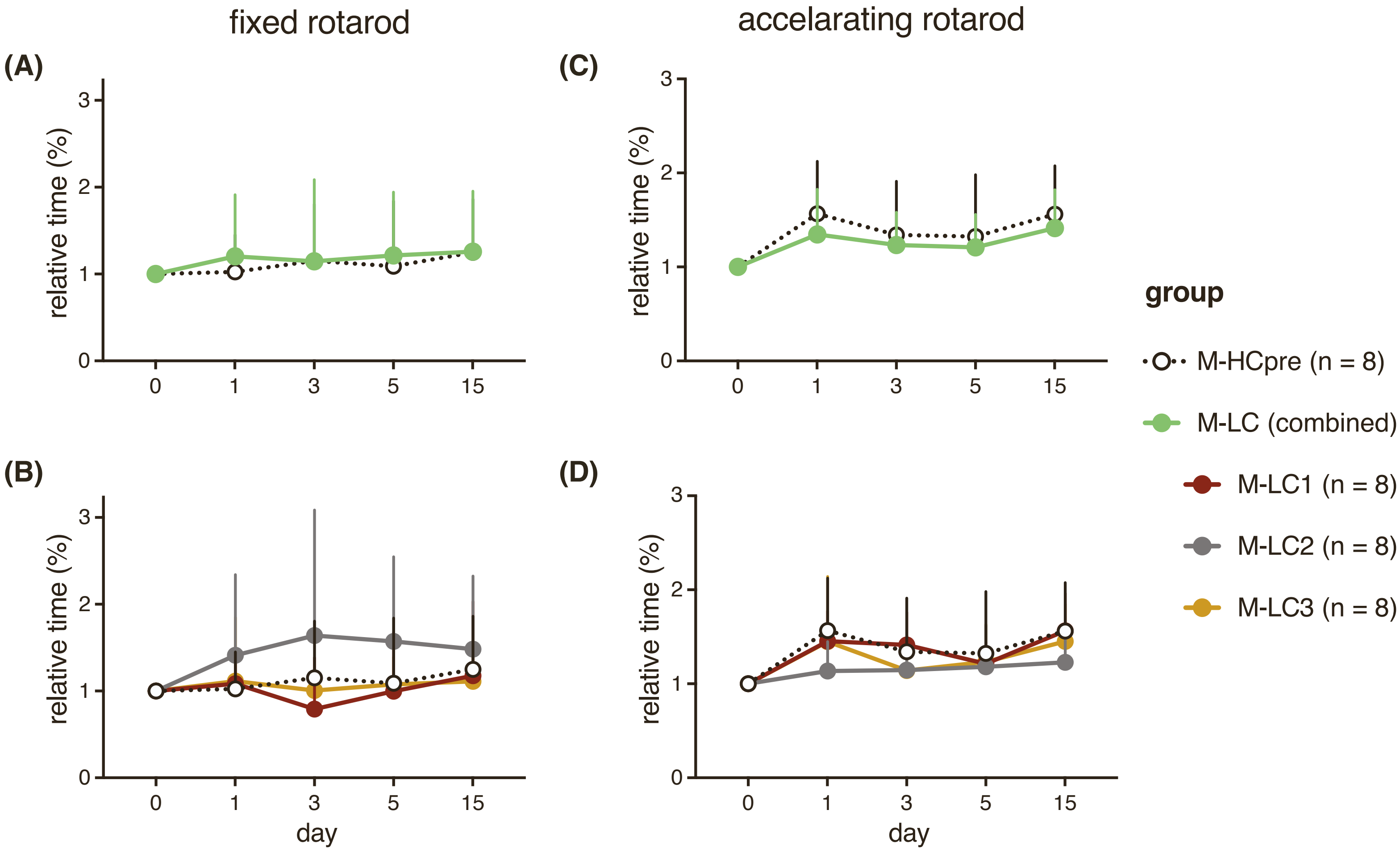

Figure S2. IgG from Long COVID patients did not change time spent on the rotarod. Rotarod test of mice injected with IgG for pre-pandemic healthy controls (M-HCpre) of Long COVID patients (M-LC) (A-B) relative time spent on the rotarod in the fixed-speed test (C-D) relative time spent on the rotarod in the accelerating test. Data points are shown as the mean + SD. Statistical significance is calculated with a linear mixed effects model with post-hoc comparison using the emmeans package with BH adjustment. Shown is the mean with standard deviation.

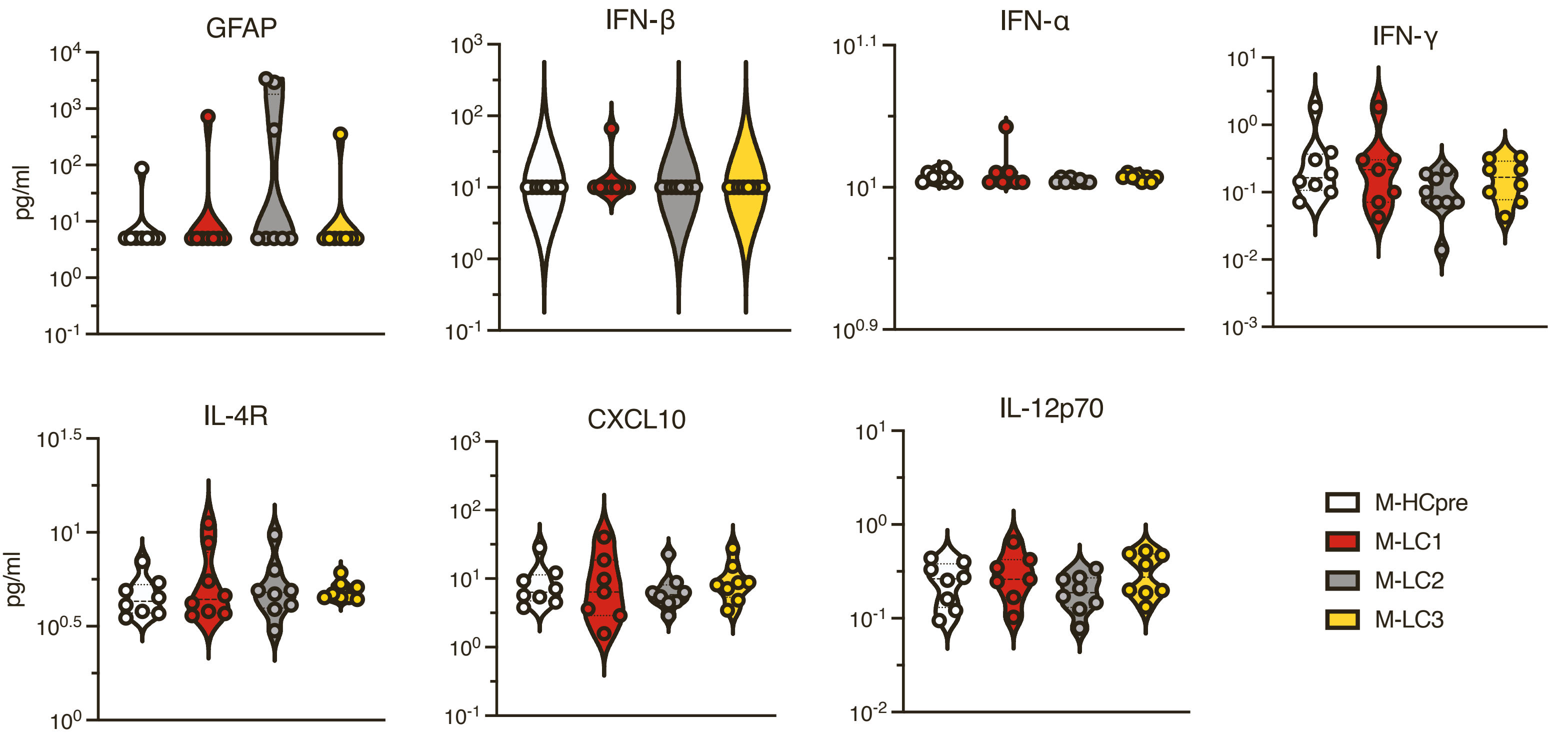

Figure S3. Plasma cytokine levels in recipient mice measured by Luminex at day 15 after injection with pooled human IgG from pre-pandemic healthy controls (M-HCpre) or Long COVID subgroups (M-LC1, M-LC2, M-LC3). Undetectable samples were imputed as half of the detection limit.

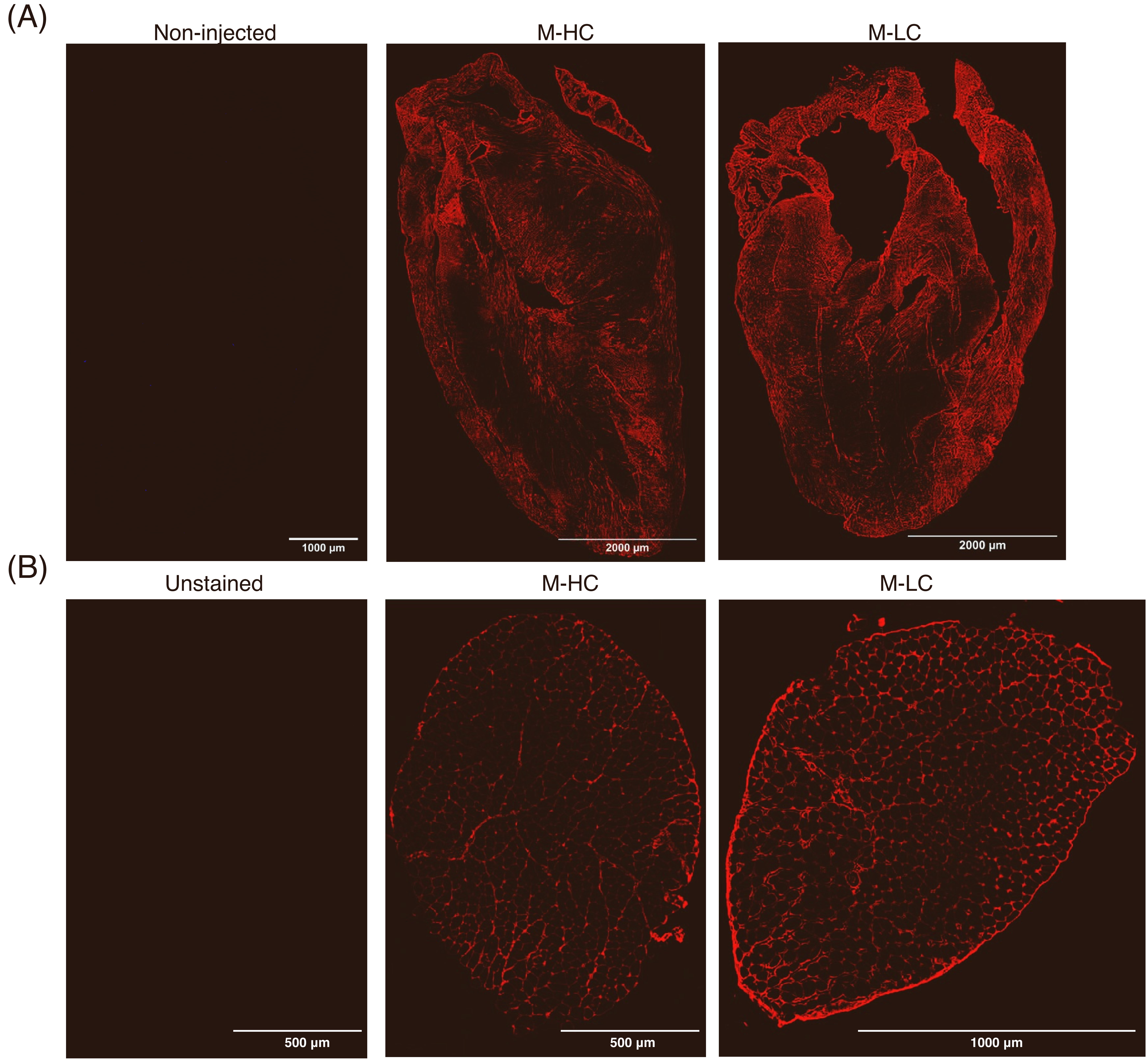
(A)
Non-injected
M-HC
M-LC
1000 μm
2000 μm
2000 μm
(B)
Unstained
M-HC
M-LC
500 μm
500 μm
1000 μm

Figure S4: Detection of injected human IgG (hIgG) in murine heart and skeletal muscle 15-day post-injection. (A) Representative staining of hIgG (red) in hearts from non-injected mice (left), mice injected with pooled IgG from pre-pandemic healthy controls (M-HCpre, middle), or Long COVID patients (M-LC, right. Shown example from the M-LC2 subgroup). (B) Representative staining of hIgG in skeletal muscle from unstained M-LC (left, shown example from the M-LC2 subgroup), M-HCpre (middle), and M-LC (right, shown example from the M-LC2 subgroup). Scale bars, 500-2000 μm.

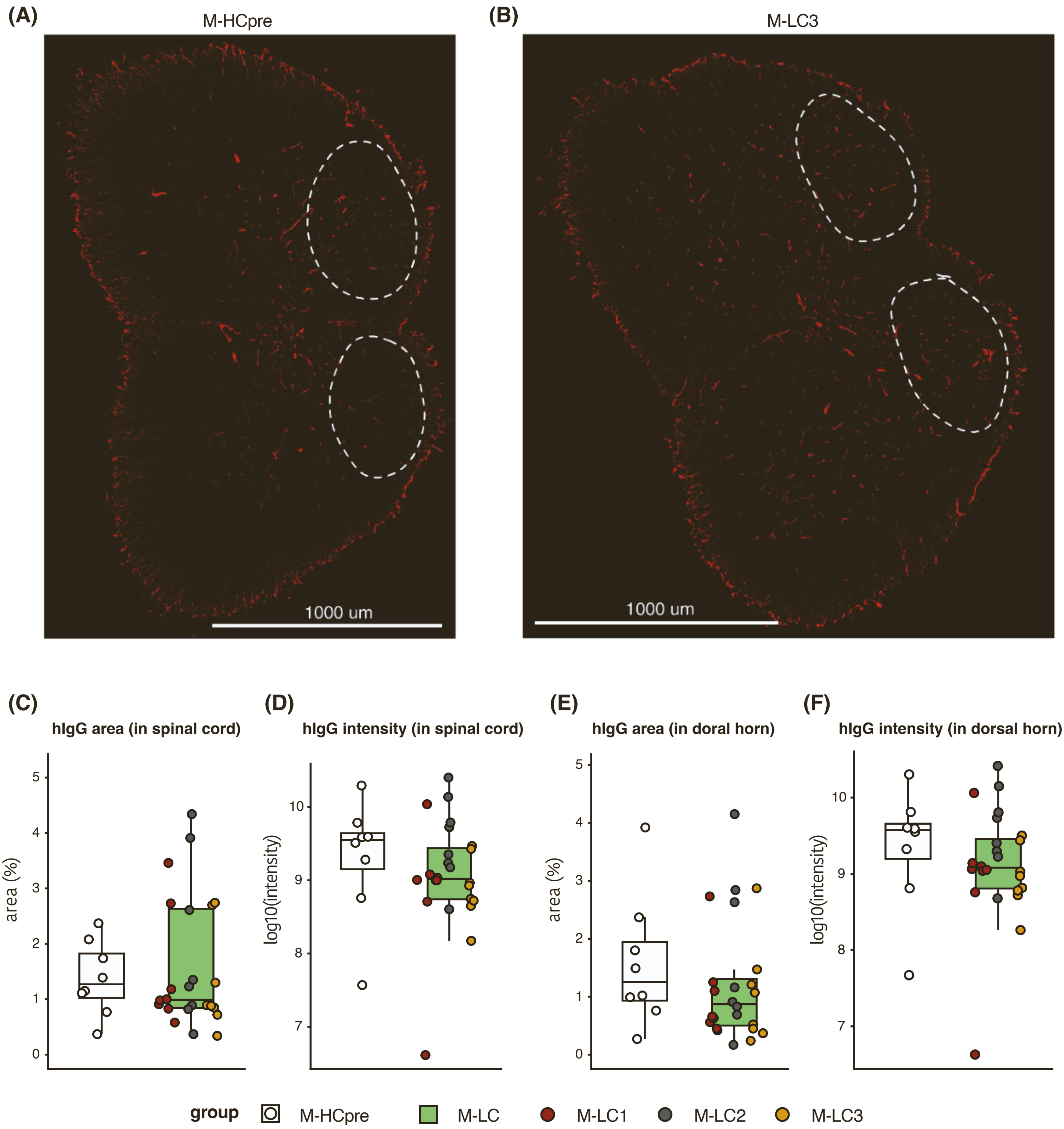

Figure S5: Detection of injected human IgG (hIgG) in murine spinal cord 15-day post-injection. (A-B) Representative staining of hIgG (red) in spinal cord sections from mice injected with pooled IgG from pre-pandemic healthy controls (M-HCpre, A) or Long COVID patients (M-LC, B; Shown example from the M-LC3 subgroup). White dashed circles indicate the dorsal horn area. Scale bars, 1000 μm. (C) Quantification of hIgG-positive area normalized to total spinal cord area per mouse. (D) Quantification of hIgG intensity in the spinal cord. (E) Quantification of hIgG-positive area within the dorsal horn region. Boxes show median and IQR. (F) Quantification of hIgG intensity within the dorsal horn region. Each dot represents one mouse; colors indicate IgG source: M-HCpre (white), M-LC1 (red), M-LC2 (grey), M-LC3 (yellow). No significant differences were observed across (sub)groups by two-sided unpaired t-tests.

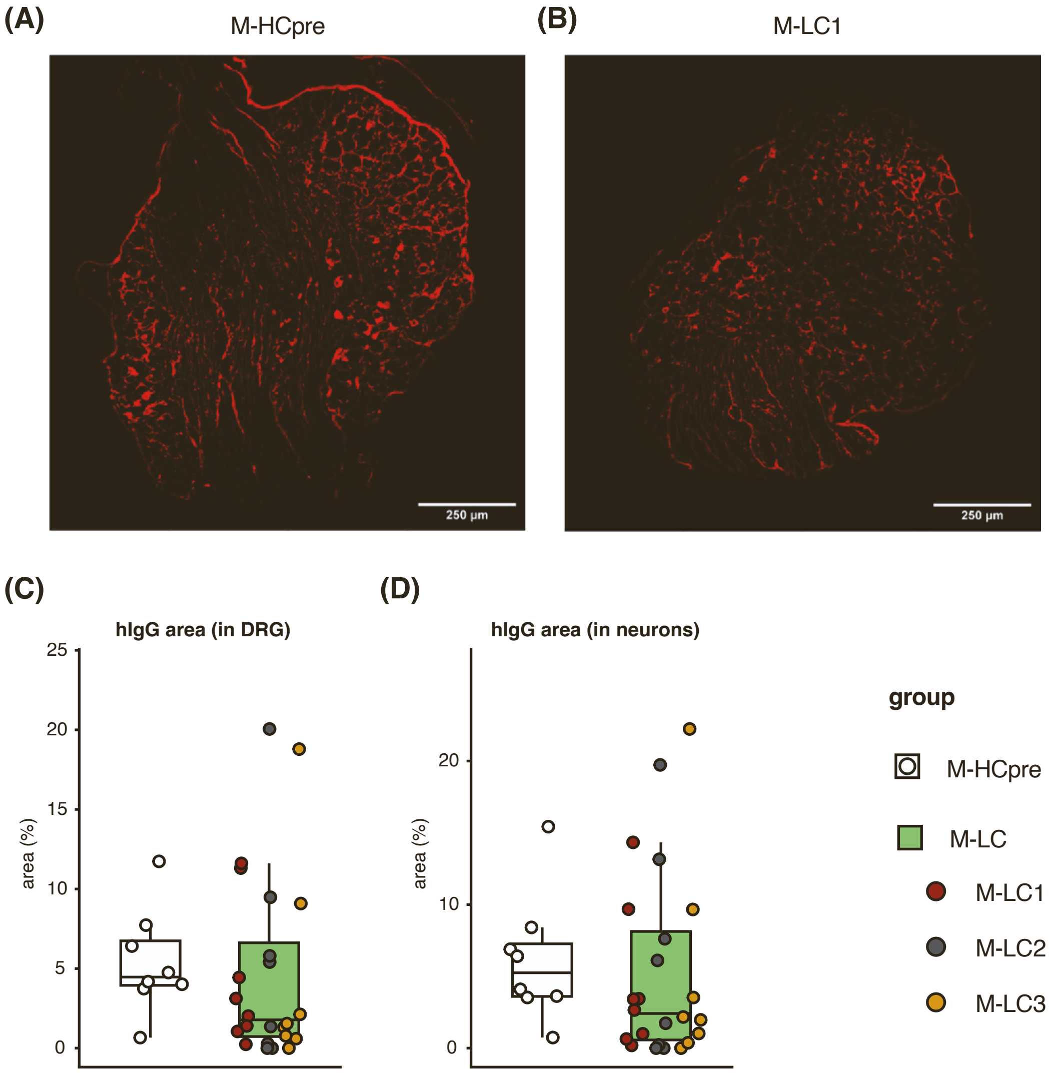

Figure S6: Detection of injected human IgG (hIgG) in murine dorsal root ganglia (DRG) 15-day post-injection. (A-B) Representative staining of hIgG (red) in DRG from mice injected with pooled IgG antibodies from pre-pandemic healthy controls (M-HCpre, A) or Long COVID patients (M-LC, B; Shown example from the M-LC1 subgroup). Scale bars, 250 µm. (C) Quantification of hIgG-positive area normalized to total DRG area per mouse. (D) Quantification of hIgG-positive area within neuronal regions (soma-containing area). Boxes show median and IQR. Each dot represents one mouse; colors indicate the IgG source: M-HCpre (white), M-LC1 (red), M-LC2 (grey), M-LC3 (yellow). No significant differences were observed across (sub)groups by two-tailed unpaired t-tests.

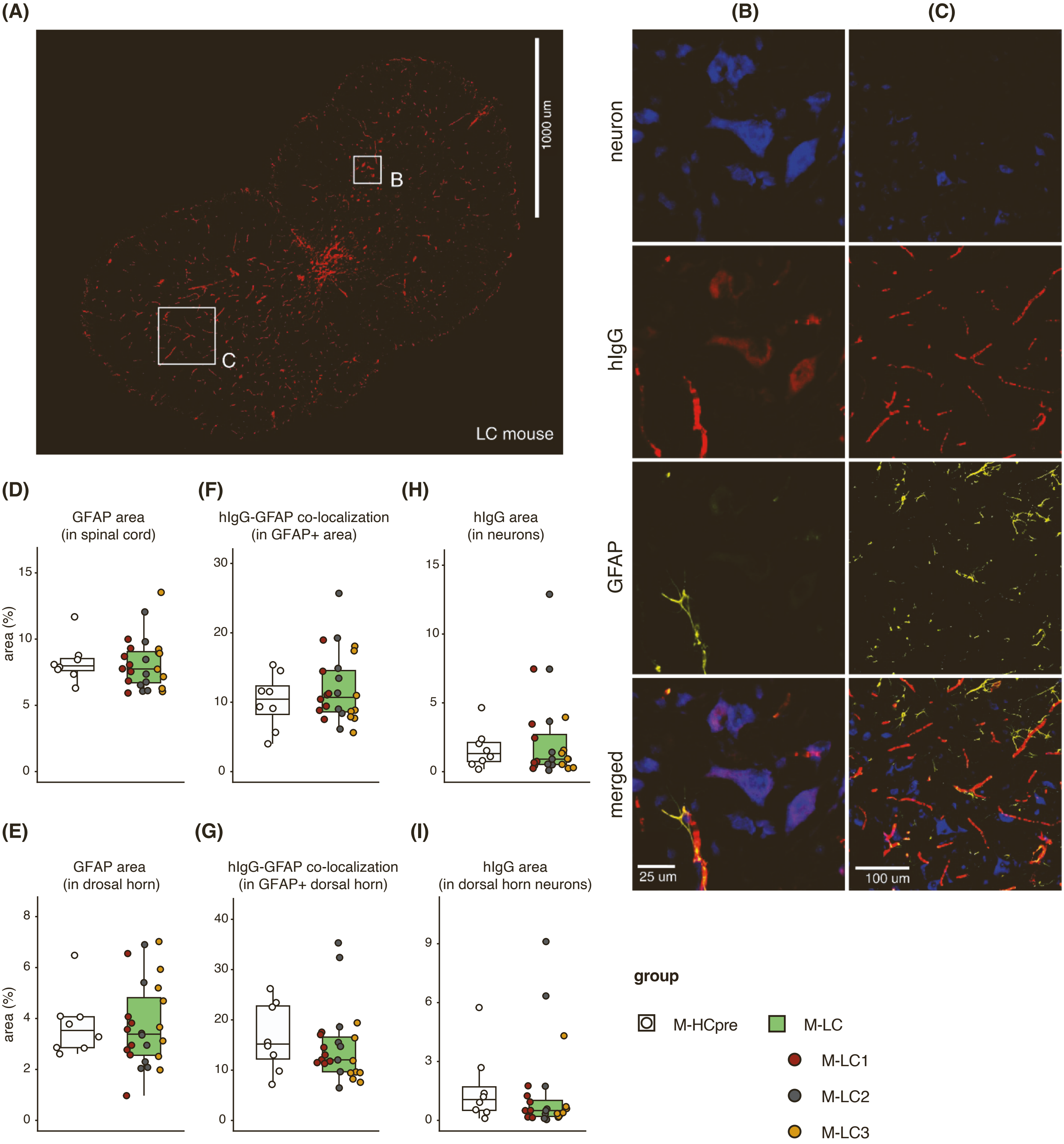

Figure S7: Co-localization of injected human IgG (hIgG) with neurons and astrocytes in murine spinal cord 15-day post-injection. (A) Representative staining of spinal cord sections from mice injected with pooled IgG from Long COVID patients (M-LC, Shown example from the M-LC1 subgroup). Sections were stained for neurons (NeuroTrace, blue), hIgG (red), and astrocytes (GFAP, yellow). White boxes indicate regions magnified in panels (B-C). (B) Magnified view showing hIgG co-localization with neurons. (C) Magnified view showing partial co-localization of hIgG with astrocytes. (D-E) Quantification of GFAP-positive area in total spinal cord (D) and dorsal horn (E). (F-G) Quantification of hIgG co-localization with GFAP in total spinal cord (F) and dorsal horn (G). (H-I) Quantification of hIgG co-localization with NeuroTrace in total spinal cord (H) and dorsal horn (I). Boxes show median and IQR. Each dot represents one mouse; colors indicate IgG source: M-HCpre (white), M-LC1 (red), M-LC2 (grey), M-LC3 (yellow). No significant differences were observed across (sub)groups by two-tailed unpaired t-tests. Scale bars, 1000 μm (A), 25-100 μm (B-C).

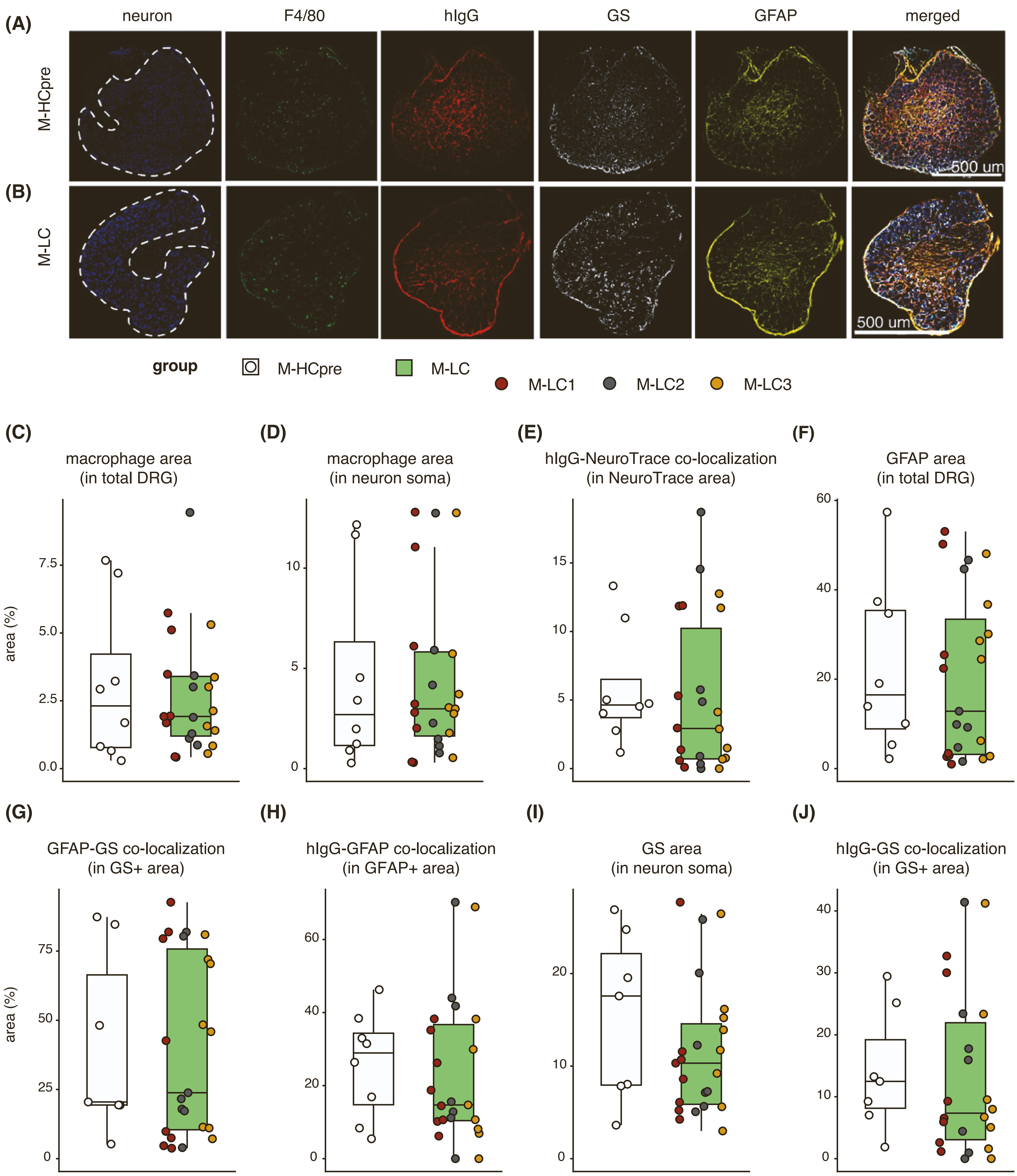
(A)
neuron
F4/80
hIgG
GS
GFAP
merged
M-HCpre
500 um
(B)
M-LC
500 um
group
M-HCpre
M-LC
M-LC1
M-LC2
M-LC3
(C)
macrophage area
(in total DRG)
(D)
macrophage area
(in neuron soma)
(E)
hIgG-NeuroTrace co-localization
(in NeuroTrace area)
(F)
GFAP area
(in total DRG)
area (%)
(G)
GFAP-GS co-localization
(in GS+ area)
(H)
hIgG-GFAP co-localization
(in GFAP+ area)
(I)
GS area
(in neuron soma)
(J)
hIgG-GS co-localization
(in GS+ area)

Figure S8: Detection of injected human IgG (hIgG) and immune cell and neuron activation markers in murine dorsal root ganglia (DRG) 15-day post-injection. (A-B) Representative staining of DRG from mice injected with pooled IgG from pre-pandemic healthy controls (M-HCpre, A) or Long COVID patients (M-LC, B; shown example from the M-LC3 subgroup). Sections were stained for neurons (NeuroTrace, blue), macrophages (F4/80, green), hIgG (red), satellite glial cells (glutamine synthetase, GS, white), and astrocytes (GFAP, yellow). White dashed outlines indicate the soma-containing region of the DRG defined by NeuroTrace labeling. Scale bars, 500 μm. (C-D) Quantification of F4/80^{+} macrophage area in total DRG (C) and within neuronal soma regions (D). (E) Quantification of hIgG co-localization with NeuroTrace staining. (FG) Quantification of GFAP^{+} and GS^{+} areas in DRG soma-containing regions. (H-J) Quantification of hIgG co-localization with GFAP^{+} (H) and GS^{+} (J) areas, and total GS^{+} area in neuronal soma (I). Boxes show median and IQR. Each dot represents one mouse; colors indicate IgG source: M-HCpre (white), M-LC1 (red), M-LC2 (grey), M-LC3 (yellow). No significant differences were observed across groups by two-sided t-tests.

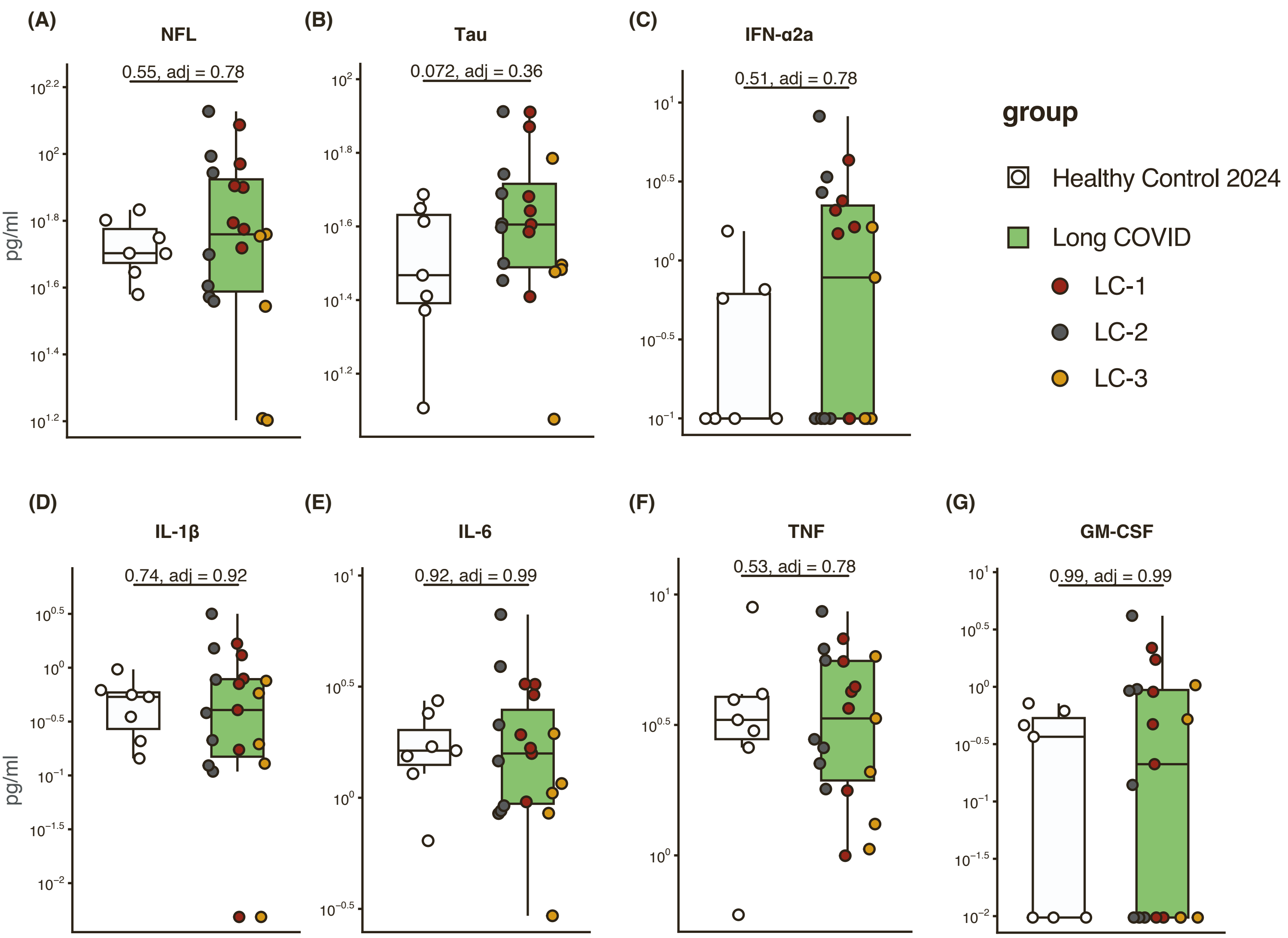

Figure S9. Two-year follow-up plasma biomarkers of Long COVID and post-COVID healthy cohort (2024) and subgroup comparisons. Targeted quantitative measurements using MSD of plasma biomarkers in follow-up plasma from (A-G) the same LC patient group (green) versus a new cohort of post-COVID, non-LC healthy controls (HC2024; white). Boxes show median and IQR; points are individuals. Individual LC donors are colored by subgroup: LC-1 (red), LC-2 (grey), LC-3 (yellow). Numbers above brackets are p values with BH-adjusted p values in parentheses from linear models adjusted for age, sex, and days-since-infection.

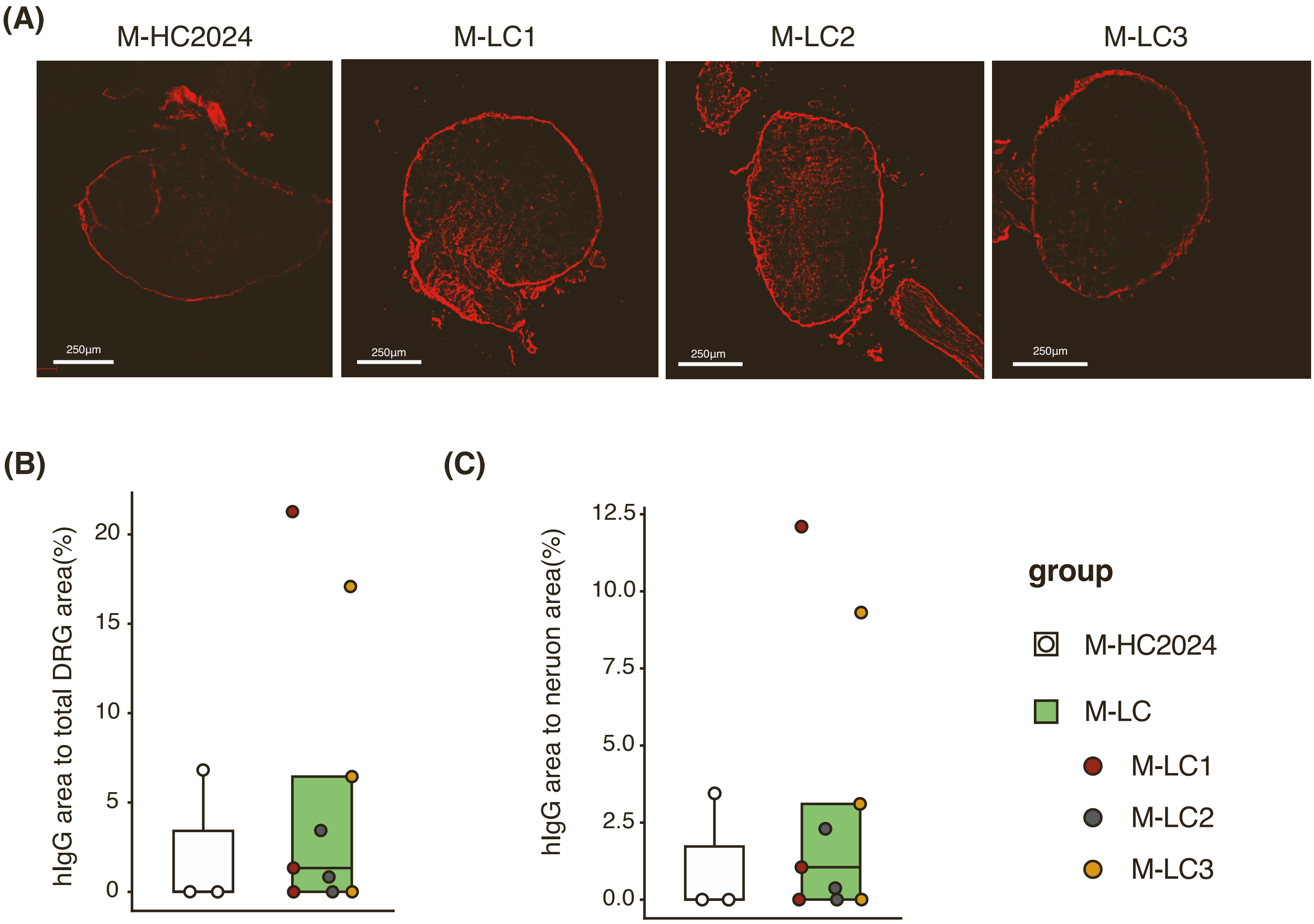

Figure S10: Detection of injected human IgG (hIgG) in murine dorsal root ganglia (DRG) 1-day post-injection. (A) Representative staining of hIgG (red) in DRG from mice injected with pooled IgG from post-SARS-CoV-2-infection healthy controls (M-HC2024) or Long COVID patients (M-LC1-3). Scale bars, 250 µm. (B) Quantification of hIgG-positive area normalized to total DRG area per mouse for M-HC2024 and combined M-LC subgroups. (C) Quantification of hIgG-positive area normalized to total DRG area per mouse across the three Long COVID subgroups (M-LC1, M-LC2, M-LC3) and controls. Boxes show median and IQR. Each dot represents one mouse; colors indicate subgroup: M-HC2024 (white), M-LC1 (red), M-LC2 (gray), M-LC3 (yellow). No significant differences were observed between groups by two-tailed unpaired t-tests.

Supplementary Table 1. overview of cohorts

Cohort	Description	Study	Assays
Long COVID	Patient	AUMC post-COVID-19 Biobank	MSD 2022/2024,<br>proteomics,<br>passive transfer 2022/2024
HC-2022	Post-COVID, non-Long COVID healthy control	S3	MSD 2022
HC-pre	Pre-pandemic healthy control	Sanquin Biobank	passive transfer 2022
HC-2024	Post-COVID, non-Long COVID healthy control	MUSCLE-PASC/ME	MSD 2024,<br>passive transfer 2024

Supplementary Table 2. Baseline characteristics of Long COVID subgroups (2022)

	LC-1<br>N = 12	LC-2<br>N = 10	LC-3<br>N = 12	*P* value
Sex = Male (%)	3 (25.0)	1 (10)	2 (16.7)	0.65
Age (median [IQR])	51 [45, 58]	40 [31, 45]	42 [34, 46]	0.08
Charlson Comorbidity Index (median [IQR])	1 [0, 2]	0 [0, 0]	0 [0, 0]	**0.01**
Time from infection to sampling, days (median [IQR])	232 [160, 316]	330 [274, 375]	231 [190, 381]	0.15
Vaccination = Yes (%)	10 (83)	9 (90)	11 (91.7)	0.80
Working hours prior SARS-CoV-2 (median [IQR])	34 [28, 38]	32 [32, 43]	35 [24, 40]	0.89

Supplementary Table 3. Differentially regulated protein tables OLINK

Supplementary Table 4. Demography of pre-pandemic controls (HC-pre)

	pre-pandemic healthy controls<br>N = 34
Sex = Male (%)	6 (17.6)
Age (median [IQR])	43 [34, 50]

Supplementary Table 5. Normalized IgG autoantibody signal intensities

Intensities are presented as log2-transformed values. Missing values indicate spots in which the foreground fluorescence intensity fell below the local background signal. Protein identifiers are reported as the JHU protein ID and the corresponding NCBI gene symbol.

Supplementary Table 6. Demography of selected Long COVID patients and non-Long COVID healthy controls (2024)

	HC-2024	LC-1 (2024)	LC-2 (2024)	LC-3 (2024)	p	test	Missing
n	7	7	7	5			
sex = Male (%)	3 (42.9)	1 (14.3)	1 (14.3)	1 (20.0)	NaN		0.0
age (median [IQR])	34.00 [26.00, 50.50]	51.00 [51.00, 60.00]	45.00 [32.50, 47.50]	37.00 [34.00, 43.00]	0.104	nonnorm	0.0
BMI (median [IQR])	20.72 [19.90, 22.76]	26.18 [24.39, 30.07]	24.03 [23.51, 25.73]	26.57 [22.84, 31.99]	0.082	nonnorm	0.0
days_since_infection_latest (median [IQR])	678.00 [546.00, 765.00]	499.00 [422.50, 870.50]	863.00 [665.50, 946.00]	336.00 [219.00, 838.00]	0.401	nonnorm	0.0
Working_hours_prepandemic (median [IQR])	40.00 [36.00, 40.00]	34.00 [22.00, 37.00]	36.00 [30.00, 45.00]	35.00 [24.00, 40.00]	0.230	nonnorm	0.0
Working_hours_postpandemic (median [IQR])	40.00 [36.00, 40.00]	7.00 [0.00, 18.00]	0.00 [0.00, 28.00]	0.00 [0.00, 0.00]	0.004	nonnorm	0.0
PEM = Yes (%)	0 (NaN)	6 (85.7)	6 (85.7)	5 (100.0)	NaN		26.9
Charleson_comorbidity_index (median [IQR])	0.00 [0.00, 0.50]	1.00 [0.50, 1.50]	0.00 [0.00, 0.00]	0.00 [0.00, 0.00]	0.029	nonnorm	0.0
SF36_pain (median [IQR])	100.00 [90.00, 100.00]	77.50 [67.50, 83.75]	45.00 [43.75, 67.50]	22.50 [20.00, 67.50]	0.006	nonnorm	0.0
SF36_physical_functioning (median [IQR])	100.00 [97.50, 100.00]	70.00 [60.00, 70.00]	55.00 [45.00, 65.00]	60.00 [10.00, 70.00]	0.002	nonnorm	0.0
SF36_limitations_phys_health (median [IQR])	100.00 [62.50, 100.00]	0.00 [0.00, 0.00]	0.00 [0.00, 0.00]	0.00 [0.00, 0.00]	<0.001	nonnorm	0.0
SF36_limitations_emotional_health (median [IQR])	33.33 [16.67, 66.67]	0.00 [0.00, 33.33]	0.00 [0.00, 50.00]	100.00 [0.00, 100.00]	0.462	nonnorm	0.0
SF36_energy (median [IQR])	85.00 [82.50, 87.50]	50.00 [35.00, 60.00]	45.00 [30.00, 67.50]	25.00 [25.00, 30.00]	0.004	nonnorm	0.0
SF36_emotional_welbeing (median [IQR])	88.00 [76.00, 90.00]	76.00 [64.00, 80.00]	64.00 [56.00, 90.00]	72.00 [64.00, 72.00]	0.357	nonnorm	0.0
SF36_social_functioning (median [IQR])	100.00 [81.25, 100.00]	62.50 [62.50, 68.75]	37.50 [25.00, 81.25]	25.00 [0.00, 50.00]	0.007	nonnorm	0.0
SF36_general_healthy (median [IQR])	90.00 [80.00, 92.50]	40.00 [32.50, 57.50]	40.00 [27.50, 47.50]	25.00 [25.00, 25.00]	0.001	nonnorm	0.0
MFI (median [IQR])	94.00 [90.00, 97.00]	65.00 [57.00, 69.00]	50.00 [44.00, 62.50]	48.00 [40.00, 54.00]	0.001	nonnorm	0.0
EQ5Dvandaag (median [IQR])	85.00 [83.00, 90.00]	72.00 [67.50, 74.50]	54.00 [40.00, 67.00]	25.00 [20.00, 55.00]	0.003	nonnorm	0.0
