# Supplementary material for: Transfer of IgG from long COVID patients induces symptomology in mice
Source: Cell Rep Med. 2026 Mar 24;7(4):102693. doi: 10.1016/j.xcrm.2026.102693 (PMC13130648; doi:10.1016/j.xcrm.2026.102693)
Supplement: Document S2. Article plus supplemental information [file mmc4.pdf]

Cell Reports
Medicine

Article

Transfer of IgG from long COVID patients induces symptomology in mice

Graphical abstract

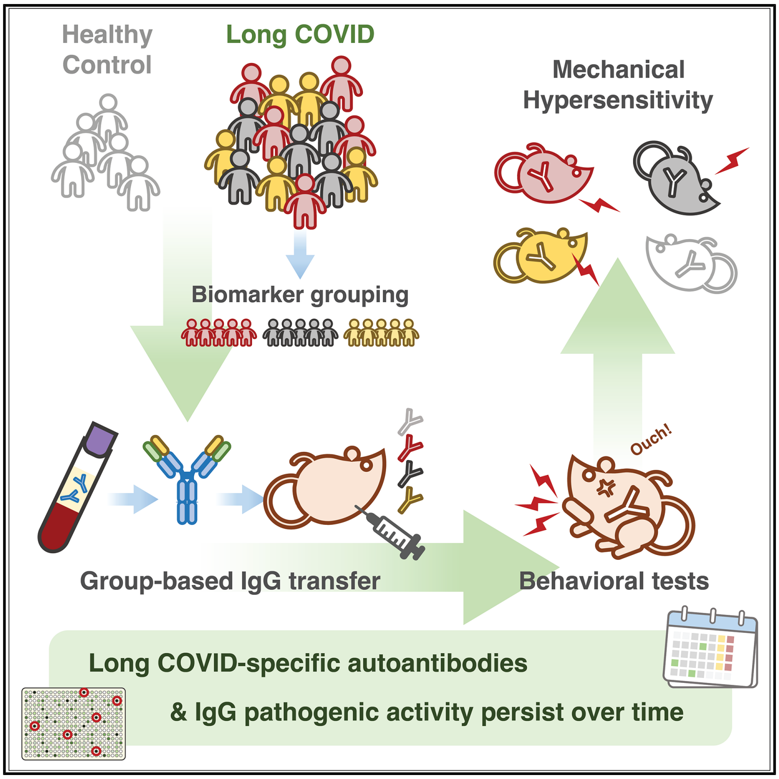

Highlights

- Transfer of long COVID IgG induces pain-associated behaviours in mice
- Pathogenic IgG effects vary across biomarker-defined patient subgroups
- Autoantibody signatures are subgroup specific and persist long-term
- IgG collected from patients 2 years later retains pain-inducing activity *in vivo*

Authors

Hung-Jen Chen, Brent Appelman, Hanneke L.D.M. Willemen, ..., Gestur Vidarsson, Niels Eijkelkamp, Jeroen den Dunnen

Correspondence

n.eijkelkamp@umcutrecht.nl

In brief

Chen et al. show that long COVID IgG induces pain-associated behaviours in mice. Pathogenic IgG effects vary across biomarker-defined subgroups, which exhibit specific, long-lasting autoantibody signatures. Remarkably, IgG collected 2 years later retains this pain-inducing activity, demonstrating that persistent autoantibodies contribute to long COVID pathogenesis.

Chen et al., 2026, Cell Reports Medicine 7, 102693
April 21, 2026 © 2026 The Author(s). Published by Elsevier Inc.
https://doi.org/10.1016/j.xcrm.2026.102693

CellPress

Cell Reports Medicine

CellPress
OPEN ACCESS

Article

Transfer of IgG from long COVID patients induces symptomology in mice

Hung-Jen Chen,[1,6] Brent Appelman,[1,6] Hanneke L.D.M. Willemen,[2,6] Amelie Bos,[1] Judith Prado,[2] W. Ashwin Mak,[1] Noa Keijzer,[1] Patrícia Silva Santos Ribeiro,[2] Sara Vieira Goncalves,[2] Sabine Versteeg,[2] Chiara.E. Geyer,[1] Mads Larsen,[3] Eline Schüchner,[1] Marije K. Bomers,[5] Ayesha H.A. Lavell,[5] Amsterdam UMC COVID-19 biobank, Braeden Charlton,[4] Rob Wüst,[4] W. Joost Wiersinga,[1] Michèle van Vugt,[1] Gestur Vidarsson,[3] Niels Eijkelkamp,[2,6,7,8,*] and Jeroen den Dunnen[1,6,7]

[1]Center for Infection and Molecular Medicine, Amsterdam Institute for Infection and Immunity, Amsterdam University Medical Center, location AMC, Amsterdam, the Netherlands
[2]Center for Translational Immunology, University Medical Center Utrecht, Utrecht University, Utrecht, the Netherlands
[3]Department of Experimental Immunohematology, Sanquin Research, Amsterdam, the Netherlands
[4]Faculty of Behavioral and Movement Sciences, Vrije Universiteit, Amsterdam, the Netherlands
[5]Department of Infectious Diseases, Amsterdam Institute for Infection and Immunity, Amsterdam University Medical Centers, location VUMC, Amsterdam, the Netherlands
[6]These authors contributed equally
[7]Senior author
[8]Lead contact
*Correspondence: n.eijkelkamp@umcutrecht.nl
https://doi.org/10.1016/j.xcrm.2026.102693

SUMMARY

SARS-CoV-2 infections have led to a surge in long COVID, a post-infectious syndrome in which autoantibodies are proposed to play a pathogenic role, analogous to fibromyalgia. Here, we test this hypothesis by transferring total IgG from long COVID patients into mice. We stratified patients into three subgroups using plasma levels of glial fibrillary acidic protein (GFAP), neurofilament light chain (NFL), and interferon-β, with subgroup-specific pathways supported by plasma proteomics. Transfer of pooled total IgG induces pronounced and persistent mechanical hypersensitivity. Notably, IgG collected 2 years later from the same long COVID patients who remained symptomatic reproduced mechanical allodynia in mice, demonstrating longitudinal stability of pathogenic activity. Proteome-wide autoantibody profiling identifies elevated, subgroup-linked autoreactivities that persist over time and are validated by independent assays. Together, these findings demonstrate that long COVID IgG can induce mechanical hypersensitivity in mice, support a causal role for autoantibodies in long COVID pathogenesis, and may establish a murine model for therapeutic development.

INTRODUCTION

The emergence of coronavirus disease 2019 (COVID-19), caused by severe acute respiratory syndrome coronavirus 2 (SARS-CoV-2), has sparked a global health crisis, with over 780 million cases and 7 million deaths reported to date.[1] Ample evidence highlights a concerning trend among COVID-19 survivors, with a significant subset (>10%) experiencing a spectrum of persistent symptoms exceeding 12 weeks post-initial recovery.[2–4] This condition is referred to as post-acute sequelae of SARS-CoV-2 infection (PASC), post-COVID syndrome, or colloquially as long COVID. Long COVID presents a heterogeneous array of symptoms, including cough, fatigue, post-exertional malaise (PEM), neurocognitive impairment (brain fog, sleep, and anxiety disorders), sensory and musculoskeletal manifestations (joint pain, chest pain, and muscle ache), postural orthostatic tachycardia syndrome, fever, shortness of breath, gastrointestinal disturbances, and palpitations.[3,5–7] However, the underlying pathophysiology of long COVID remains elusive.

Several potential mechanisms have been proposed, connecting long COVID symptomatology to dysregulated interferon (IFN) response, inflammation, cellular metabolism, persistent infection, dysbiosis, neuroinflammation, and autoimmune processes.[3,8–14] Among these factors, studies have demonstrated that autoimmunity is induced in both acute and post-acute phases of COVID-19.[15–17] While acute disease severity correlates with anti-viral protein antibodies,[18] long COVID is characterized by the presence of autoantibodies targeting diverse self-antigens.[19] These long COVID-associated autoantibodies bind to chemokines,[20] G protein-coupled receptors,[21] neurotransmitters,[22] and various immunomodulating proteins.[16,23] Moreover, it has been hypothesized that autoantibodies play a crucial role in other chronic fatigue and post-acute infection syndromes, such as post-Lyme disease syndrome, Q-fever fatigue syndrome, fibromyalgia, and myalgic encephalomyelitis/chronic fatigue syndrome (ME/CFS).[6] Yet, whether these autoantibodies are mere bystanders or active contributors to long COVID symptoms is not known.

© 2026 The Author(s). Published by Elsevier Inc.
This is an open access article under the CC BY license (http://creativecommons.org/licenses/by/4.0/).

Table 1. Baseline characteristics of long COVID patients and non-long COVID healthy controls (2022)

	Long COVID *N* = 34	2022 Post-COVID healthy controls *N* = 15	*p* value
Sex = male (%)	6 (17.6)	3 (20.0)	1.000
Age (median [IQR])	43 [34, 50]	35 [32, 49]	0.385
Charlson comorbidity index (median [IQR])	0 [0, 1]	–	N/A
Time from infection to sampling, days (median [IQR])	275 [195, 379]	278 [271, 288]	0.948
Vaccination before initial SARS-CoV-2 infection = yes (%)	0 (0)	0 (0)	1.000
Vaccination before sampling = yes (%)	30 (88.2)	0 (0)	<0.001
Working hours prior SARS-CoV-2 infection (median [IQR])	34 [27, 40]	–	N/A

IQR, interquartile range; N/A, not applicable.

Recent experimental evidence supports the potential involvement of autoantibodies in driving long COVID symptomatology. Following therapeutic apheresis, clinical improvement in long COVID patients appears to be associated with autoantibody reduction.[22] Moreover, transfer of immunoglobulin G (IgG) from fibromyalgia patients to mice induces pain-associated behavior.[24] Similarly, injection of patient-derived anti-CASPR2 autoantibodies into mice produces mechanical pain-related hypersensitivity in the absence of neural injury.[25] Hence, we postulate that autoantibodies may play a causal role in the manifestation of long COVID symptoms in at least a subset of patients. In this study, we set out to elucidate the involvement of autoantibodies in long COVID pathogenesis by establishing a patient-derived IgG-transferring mouse model for long COVID.

RESULTS

Long COVID patients are characterized by altered interferons and GFAP

We enrolled 34 patients from the Amsterdam UMC outpatient post-COVID clinic based on World Health Organization definition. All participants had confirmed prior SARS-CoV-2 infection and were in good physical and mental health prior to infection. None of the individuals were hospitalized for COVID-19, and their symptoms persisted for a minimum of 6 months following the initial infection (Table 1). The diagnosis of long COVID was made by a physician dedicated to post-COVID-19 at the Amsterdam UMC. Patients presented a varied array of symptoms, with fatigue being consistently reported among all participants. Additionally, 29 of 34 patients experienced PEM, 25 of 34 reported pain symptoms, and 26 of 34 were unable to resume their previous occupational roles at the time of inclusion. As controls, we included 15 healthy donors who had gone through SARS-COV2 infection(s) without residual/persistent symptoms from the same source population with comparable distributions of age, sex, and days since SARS-CoV-2 infection (HC-2022, Table 1).

Previous studies have shown that long COVID is characterized by altered IFN levels,[8,26] chronic inflammation,[27,28] and signs of neuronal damage and neuroinflammation.[29–31] Thus, we performed targeted biomarker quantification against 10 serum proteins, including IFN-Is and -IIs, prototypic COVID-19 pro-inflammatory cytokines (interleukin [IL]-1β, IL-6, tumor necrosis factor, and granulocyte-macrophage colony-stimulating factor [GM-CSF]),[32] and neuronal damage (neurofilament light chain [NFL]) and astrogliosis markers (glial fibrillary acidic protein [GFAP]). Principal component (PC) analysis revealed the strongest separation between long COVID patients and healthy controls along PC2, driven primarily by IFN-γ, IFN-β, IFN-α2a, GM-CSF, and GFAP (Figure 1A). When we performed differential expression analysis using a linear model adjusted for age, sex, and time from the initial SARS-CoV-2 infection to sampling, IFN-γ was the strongest negative contributor and was reduced in long COVID patients (Figure 1B). Both IFN-Is, IFN-β and IFN-α2a, did not reach statistical significance (Figures 1C and S1A). GFAP was detectable in 10 of 34 long COVID patients and undetectable in all healthy controls (Figure 1D). Acute-phase COVID-19 pro-inflammatory cytokines and neurodegenerative markers (TAU and NFL) were comparable between long COVID patients and healthy controls (Figures S1B–S1G).

Notably, despite the lack of significant group-level differences, we observed high inter-individual variability of these biomarkers in our cohorts. GFAP and NFL were detectable only in a subset of patients, while IFN-β levels exhibited a broad dynamic range and still tended to be elevated. Given prior evidence linking astroglial activation/axonal injury (GFAP and NFL)[33,34] and IFN-I activity[8,35,36] to long COVID pathobiology, we sought to use these biomarkers to stratify patients and capture potential neuroimmune subgroups. We implemented a two-step strategy to stratify patients. First, individuals with elevated GFAP and/or NFL relative to the control distribution were classified as LC-1 (n = 12, Figures 1E and 1F). Second, the remaining patients were subdivided by IFN-β level into LC-2 (high IFN-β, n = 10) and LC-3 (low IFN-β, n = 12) (Figure 1G). This approach aimed to enrich for individuals with potential central nervous system involvement or immune dysregulation.

Proteomics profiling supports molecular distinction of long COVID subgroups

To evaluate the molecular distinction of the predefined long COVID subgroups, we retrospectively profiled 2,865 plasma proteins in 31 of 34 long COVID patients (3 excluded for insufficient sample volumes). Partial least-squares discriminant analysis (PLS-DA) separated LC-1 from LC-2/LC-3 along PC1 and distinguished LC-2 from LC-3 along PC2 (Figure 2A). Gene set enrichment analysis of the PC loadings indicated that LC-1 was depleted for cell surface proteins but enriched for intracellular transport proteins (Figure 2B), LC-2 was enriched for

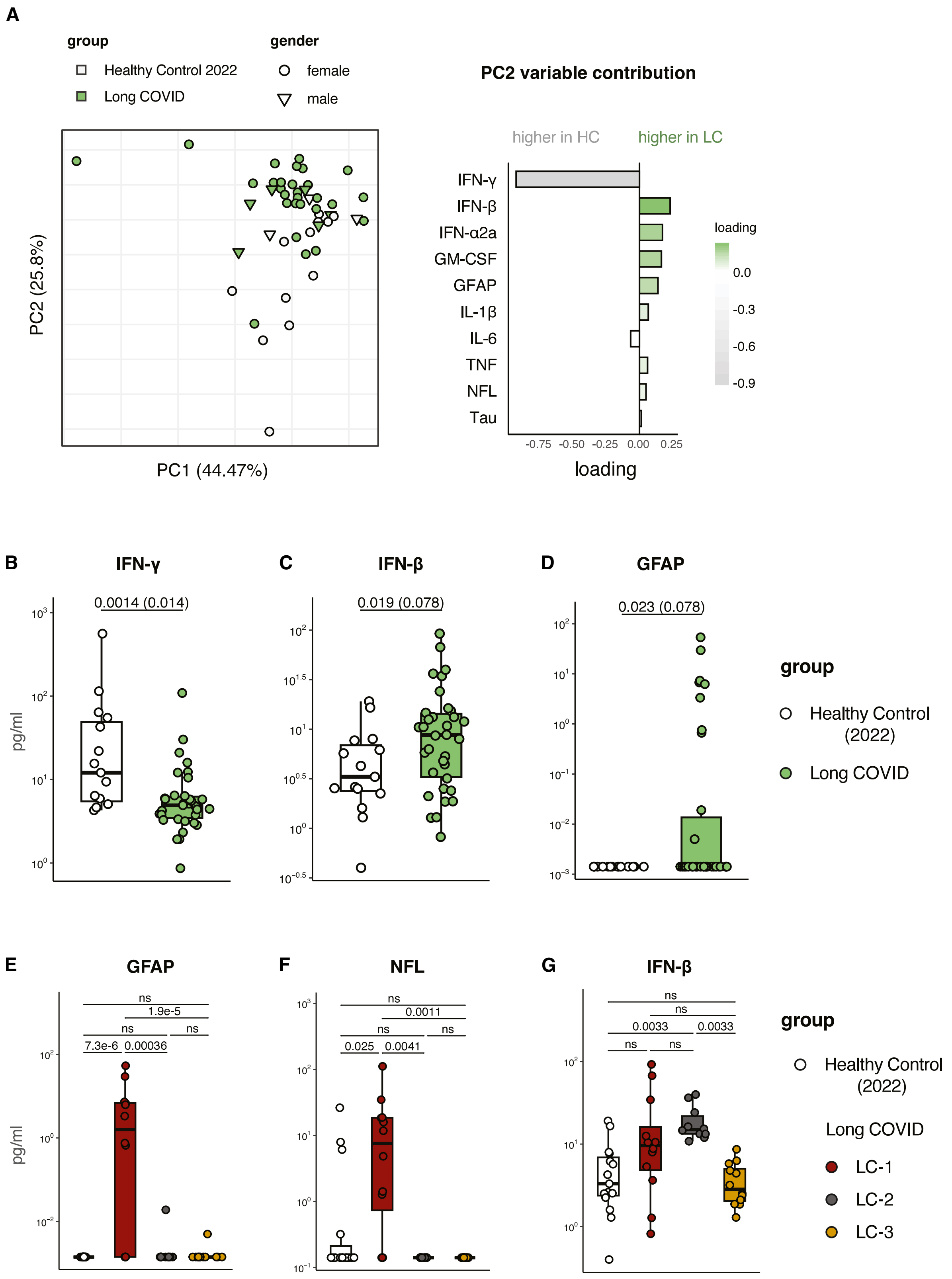

(legend on next page)

muscle-related signatures, and LC-3 was enriched for lipoprotein-related signatures (Figure 2C). Clustering the top 200 PC1 and PC2 loadings revealed subgroup-associated protein modules (Figure 2D). For example, clusters C1, C2, and C3, which included GFAP and NFL, were elevated in LC-1. Clusters C5 and C6, containing immune activation markers such as IL-12 (IL-12p35 and IL-12p40) and HIF-1α, were increased in LC-2. Clusters C3, C4, and C6, containing immune cytokines and receptors like TNFSF9, IL4R, and TGFBR1, showed higher levels in LC-3. Differential protein analyses comparing each subgroup to the other two corroborated our stratification strategy: LC-1 showed higher GFAP and NFL, while LC-2 showed increase in the IFN-response chemokine CXCL10 (Table S3). Because plasma proteins can reflect tissue injury and protein release into circulation, tissue enrichment analysis of differentially regulated proteins mapped LC-1 to nervous system tissue, LC-2 to skeletal muscles, and LC-3 to liver, pancreas, testis, spinal cord, and skin (Figure 2E).

Long COVID IgG transfer induces sensory hypersensitivity in mice

We hypothesized that if (auto)antibodies are causative for long COVID, patient-derived IgG antibodies will result in tissue damage and subsequent associated symptoms in mice. We purified IgG from patient plasma, pooled samples by long COVID subgroups, and compared them with 34 pooled pre-pandemic healthy control (HC-pre, Table S4) IgG. Each pooled human IgG (hIgG) was injected into 8 C57Bl/6 mice (4 females and 4 males) at a single dose of 260 mg/kg (∼1/3 of total circulating IgG per mouse,[37] a dose lower than in a prior study[24]). We assessed systemic inflammation by measuring plasma GFAP, IFNs, and cytokines in recipient mice and did not observe evidence of systemic inflammation or differences between long COVID and control IgG groups at day 15 post-injection (Figure S3). Since neurosensory symptoms, i.e., pain, are prevalent in long COVID patients,[3] we investigated whether patient-derived IgG causes neurological symptoms. We measured mechanical sensory thresholds with the von Frey test and thermal sensitivity using the Hargreaves test.[38] Mice that received long COVID patient IgG (M-LC) developed a pronounced reduction in mechanical sensory threshold (increased mechanical sensitivity) that lasted for at least 15 days compared to the HC-pre IgG-injected control mice (M-HCpre) (Figure 3A). Considering the long COVID subgroups, the reduction in mechanical threshold primarily occurred in M-LC1 and M-LC3 (Figure 3B). Notably, the time course for the development of mechanical hypersensitivity was different in M-LC1 and M-LC3: M-LC1 developed mechanical hypersensitivity from day 3, while the mechanical threshold in M-LC3 was already reduced at 24 h post-hIgG injection.

In both M-LC and M-HCpre, the latency to heat stimulation was reduced, which indicates increased heat sensitivity. While this thermal hypersensitivity normalized within 2–3 days in mice injected with HC-pre hIgG, in mice that had received long COVID hIgG, the hyper-sensitive state persisted until at least 15 days post-injection (the last measurement, Figure 3C). Considering long COVID subgroups, the latency to heat stimulation was only persistently reduced in M-LC1 and M-LC3, whereas M-LC2 mice did not significantly differ from the M-HCpre group at any time point measured (Figure 3D). Interestingly, mice injected with hIgG from LC-1 group only differed from HC starting from day 3, while mice injected with IgG from LC-3 developed thermal hypersensitivity that was stronger than in mice injected with HC igG starting from day 1 after injection. These tests indicate that IgG from different subgroups of long COVID patients elicited distinct sensory symptoms, with subgroup-specific course in pain-associated behavior.

Long COVID IgG transfer affects locomotor activity in mice

Considering the wide range of musculoskeletal complications observed in long COVID patients,[7,39] we hypothesized that transferring long COVID IgG could also affect locomotor behavior. We assessed general locomotor activity levels using an open field test.[40] While overall activity was comparable across most groups (Figure 4A), mice injected with LC-2 IgG (M-LC2) showed a statistically significant but modest reduction in walking distance, approximately 40% less than M-HC at 1 day post-injection (Figure 4B). M-LC1 and M-LC3 did not differ from M-HC at any of the tested time points. Movement pattern analysis suggested that the reduced distance in M-LC2 was primarily due to increased immobility (Figures 4C and 4D). In addition to locomotor activity, motor strength, coordination, and balance are also important factors in movement behavior. We assessed these motor functions in mice using two rotarod paradigms[41]: one with fixed speed and another with accelerating speed over time to investigate baseline motor coordination and detect subtle impairments in motor coordination, respectively. In both assays, M-LC or M-HC mice did not perform differently in these tests (Figures S2A–S2D). Together, these data indicate that transfer of LC-2 IgG to mice induced a transient and modest reduction in general locomotor activity due to increased immobility, without affecting gross coordination and balance.

Autoantibody profiling maps subgroup-linked targets

Since passive IgG transfer induced mechanical hypersensitivity, we next investigated antigen specificities of the transferred hIgGs using HuProt proteome arrays (>21,000 intra-/extracellular human proteins). IgG signals were normalized with a

Figure 1. Plasma biomarkers of long COVID compared with non-long COVID SARS-CoV-2 convalescences

(A) Targeted quantitative measurements of plasma biomarkers of 34 long COVID (LC) patients and 15 post-SARS-CoV-2 infection healthy controls (HC): (A) PCA of all markers showing partial separation of LC (green) and controls (white); symbols denote sex. PC2 variable loadings indicating contributors to case-control separation (negative, higher in controls; positive, higher in LC).

(B–D) Boxplots (scale in $\log_{10}$ pg/mL) for IFN-γ, IFN-β, and GFAP comparing LC vs. controls.

(E–G) Boxplots for LC subgroup comparisons: GFAP, NFL, and IFN-β across HC/LC-1/LC-2/LC-3.

Boxes show median and interquartile range; points are individuals. Numbers printed on the plots are *p* values with the Benjamini-Hochberg (BH)-adjusted *p* value in parentheses from linear models adjusted for age, sex, and days since infection. ns, not significant.

CellPress
OPEN ACCESS

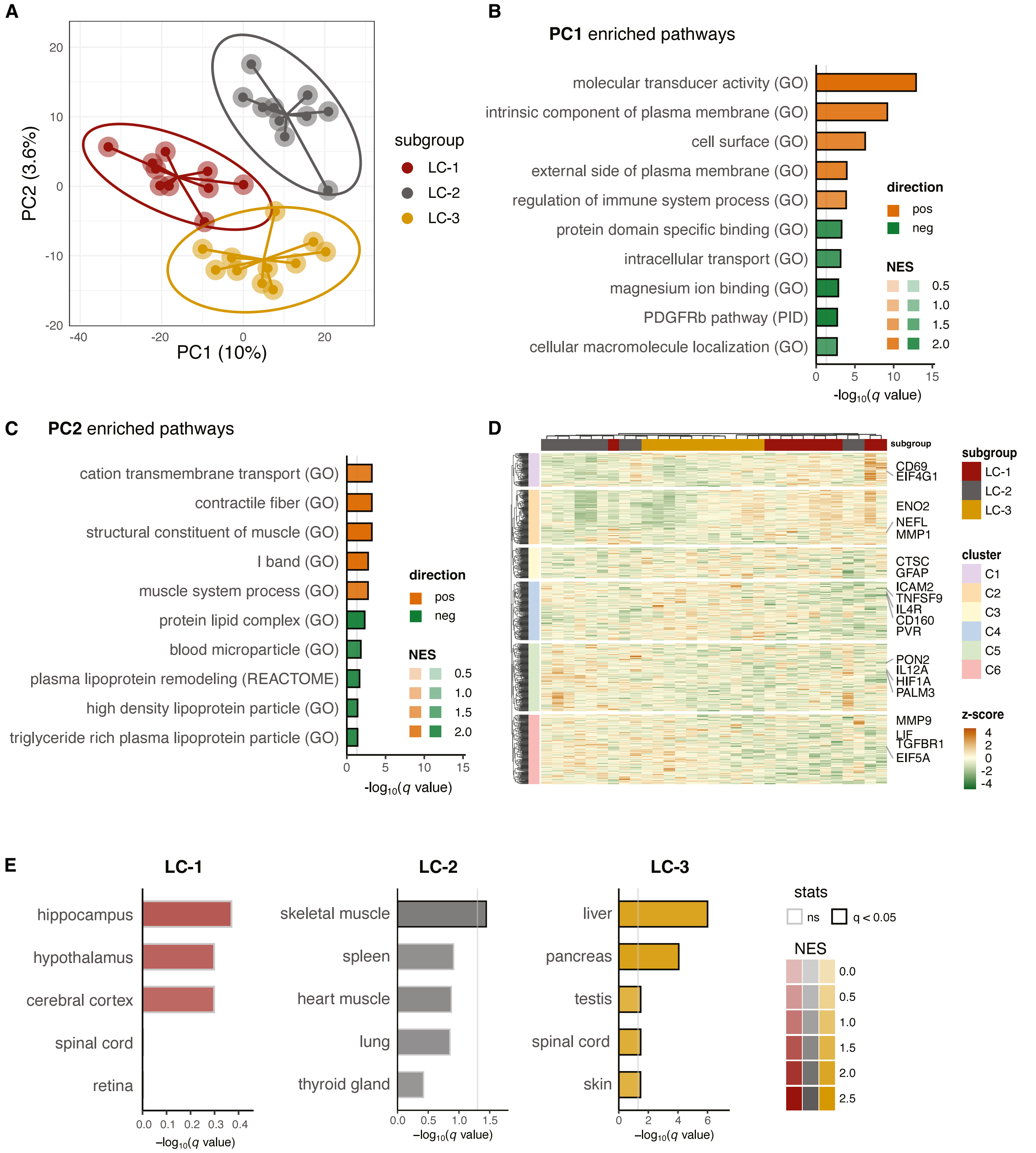

Figure 2. Plasma proteomics clustering analysis identified biomarkers distinguishing three long COVID subgroups

(A) Partial least-squares discriminant analysis of plasma proteome shows a separation of LC-1 from LC-2 and LC-3 on PC1, while LC-2 and LC-3 segregated on PC2.

(B and C) Gene set enrichment analysis of PC1 and PC2 loadings. Bar length shows −log10(q value); color tiles show normalized enrichment scores (NES). “pos” and “neg” indicate enrichment toward the positive or negative direction of the PC ($PC1^+$, LC-1; $PC2^+$, LC-2).

(legend continued on next page)

robust linear model and standardized to the on-array hIgG calibration spots[42] (Table S5). We defined long COVID-associated autoreactivities as features with intensity >2-fold of the pooled HC-pre IgG and exceeding the 1 μg/mL on-array IgG standard. Using this threshold, we identified 134 autoantibodies elevated in long COVID (Figure 5A). We next defined subgroup-specific reactivities as antigens with standardized intensity >1.5× that of the next-highest subgroup. LC-1 was enriched for epidermal keratins (i.e., KRT6A/KRT14) and Argonaute (AGO) proteins (AGO1-4). LC-2 featured anti-IFNA1 and neuronal/glial targets such as GAD2. LC-3 showed distinct autoreactivity linked to inflammation and nociception (e.g., PRKCE,[43,44] TAB1,[45] and OPRD1[46]) (Figure 5A). Pathway analysis of all targets highlighted epithelial structural constituents, Dicer/RNA silencing, and IL1R signaling pathways (Figure 5B). For the subgroups, LC-1 was strongly enriched for skin-epidermis structural components, LC-2 for sensory perception and receptor activation pathways, and LC-3 for hypoxia and β-defensin-related programs. Notably, applying more stringent cutoffs (e.g., requiring >4-fold intensity relative to HC-pre) identified 60 autoantibodies (Figure 5A, underlined) and the same enriched pathways (data not shown), highlighting the robustness of these subgroup-linked signatures.

To test whether transferred IgG accumulated in relevant tissues, we assessed hIgG deposition in hearts, skeletal muscles, spinal cords, and dorsal root ganglia (DRG) 15 days post-injection, as these tissues have been postulated to be affected in long COVID pathology. We used an anti-hIgG detection antibody validated in mouse tissue and confirmed no detectable cross-reactivity with mouse IgG (data not shown). We detected hIgG in all examined tissue types with no significant differences between healthy controls (M-HCpre) and long COVID patients (M-LC) and between LC subgroups (Figures S4–S6). Given the pain-like behaviors in recipient mice, we further quantified markers in spinal cord and DRG linked to chronic pain: GFAP for astrocyte activation, glutamine synthetase (GS) for satellite glial cells, and F4/80 for macrophage infiltration and inflammation in DRGs (Figures S7 and S8). hIgG partially co-localized with neuronal and glial markers, but expression of GFAP, GS, and F4/80, as well as hIgG co-localization with these markers, did not differ between M-LC and M-HCpre. Thus, while transferred hIgG reached sensory tissues, it did not produce detectable subgroup-specific glial or immune activation at this time point.

Two-year persistence of biomarkers, autoantibodies, and replicated IgG-induced hypersensitivity

To test temporal stability, we re-profiled cytokines and neuroinjury markers in plasma collected in 2024 from 19 of the same long COVID patients who remained symptomatic around 2 years after initial sampling and compared them with a new age- and sex-matched healthy control cohort that has experienced SARS-CoV2 infections (HC-2024, N = 7, Table S6). At follow-up, long COVID patients again trended toward higher IFN-β (Figure 6A) with LC-2 appearing to be at relatively higher levels and a subset retained higher GFAP levels (Figure 6B). IFN-γ no longer differed between long COVID patients and controls (Figure 6C). NFL, Tau, IFN-α2a, and other pro-inflammatory cytokines were not discriminatory (Figures S9A–S9G). Notably, two neuro-injury markers, GFAP and NFL, were relatively increased in LC-2, becoming comparable to LC-1 at this measurement (Figures 6B and S9A). These data indicate that a subset of long COVID biomarkers persist for at least 2 years in our cohort.

We next repeated passive IgG transfer using pooled IgG from the long COVID samples collected at 2024 and HC-2024 cohorts, with the Von Frey assay prespecified as the primary behavioral endpoint. Using the same subgroups, dose, and blinded assessment as the initial experiment, mice receiving long COVID IgG again showed reduced mechanical withdrawal thresholds vs. HC-2024 IgG (Figure 6D). Stratified by subgroup, LC-1 and LC-3 IgG reproduced robust mechanical hypersensitivity (Figure 6E). Notably, LC-2 IgG also induced clear hypersensitivity in mice in this experiment. To assess whether hIgG-tissue engagement occurs earlier, we stained mouse DRG for hIgG at day 1 post-injection. Consistent with the day 15 histology from the initial experiment, hIgG deposition was detectable, but did not differ clearly between LC and HC IgG groups (Figure S10).

Finally, to validate key autoantibody persistence, we measured selected IgG targets using Luminex bead-based assays with recombinant proteins from independent suppliers and antigen-specific monoclonal antibodies as calibrators and positive controls. Several subgroup-linked autoantibodies showed consistently high reactivity at baseline (2022) and follow-up (2024): LC-1: ALDH1L1, MAGEB10, AGO2, and KRT6A; LC-2: GSTT1, GAD2, and GIF; LC-3: MB21D2) (Figure 6F). This supports persistent, subgroup-linked autoantibody signatures.

DISCUSSION

In this study, we demonstrate that IgG from long COVID patients can induce pain-like behavior in mice, providing functional evidence that autoantibodies contribute to long COVID pathogenesis. Using a biomarker-guided, longitudinal design, we further show that this pathogenic activity persists over time within individuals. Across both time points, circulating IFN-Is and neuroinjury markers (GFAP, NFL, and Tau) distinguished long COVID patients from post-SARS-CoV-2 infection healthy controls and remained stable within individuals. Proteome-wide autoantibody profiling revealed subgroup-specific autoreactivities, with several IgG specificities persisting for at least 2 years. Together, these findings support a causal role for sustained (auto)antibody responses in long COVID pathophysiology and establish a translational model for mechanistic investigation and therapeutic testing.

Persistent elevation of IFN-Is in long COVID has been reported previously,[36,47,48] and acute-phase IFN-α/β surges are known to reduce serotonin levels, potentially contributing

(D) Heatmap of the top 200 loadings from PC1/PC2 clustered into six protein modules (C1–C6) with distinct patterns; example annotations include GFAP/NFL (LC-1 linked), IL-12/HIF-1α (LC-2 linked), and TNFSF9/IL4R/TGFBR1 (LC-3 linked).
(E) Tissue enrichment of differentially regulated proteins (one vs. rest) for each subgroup.
Black-bordered squares indicate q < 0.05. GO, Gene Ontology; PID, Pathway Interaction Database.

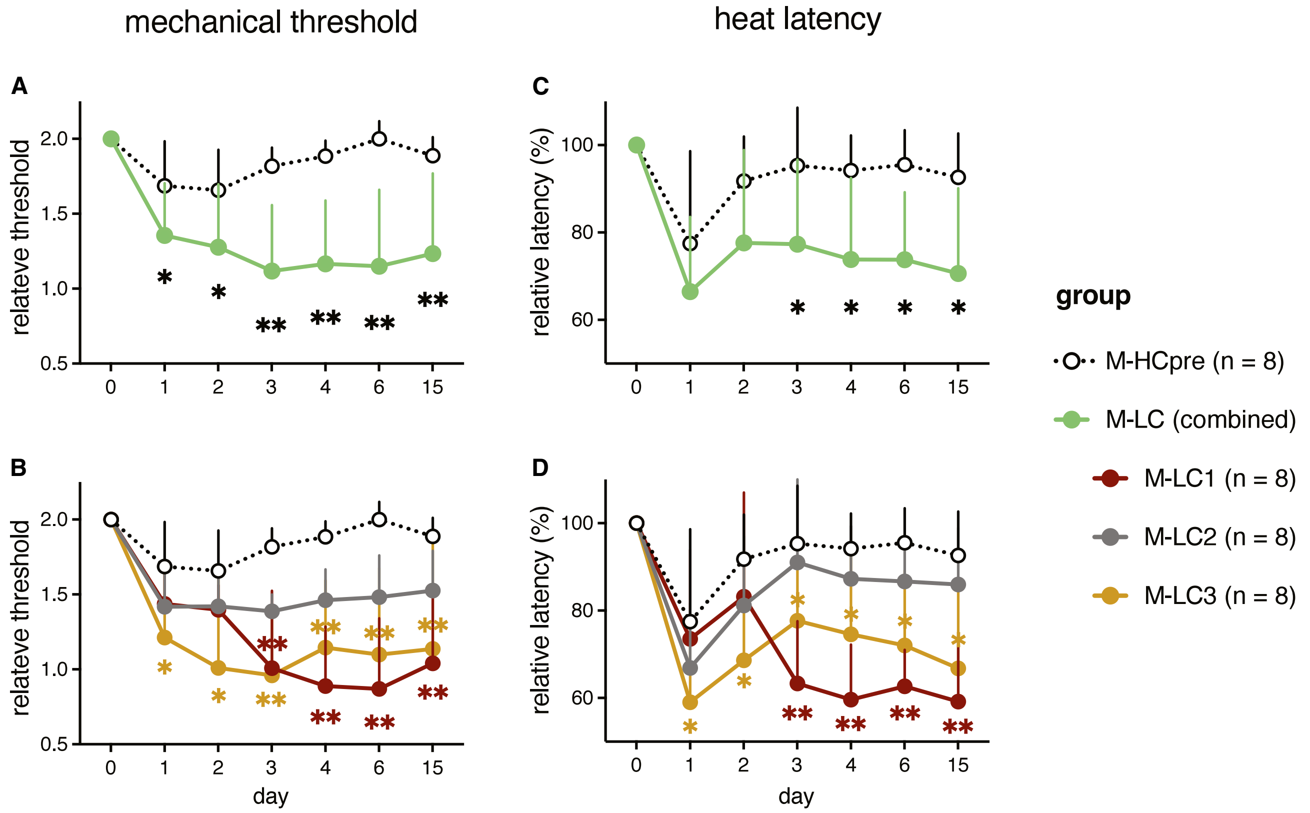

Figure 3. Passive transfer of long COVID IgG induces sensory hypersensitivity in mice

Mice (n = 8 mice/group) received a single intraperitoneal injection of pooled total human IgG from long COVD patient subgroups (M-LC1/2/3) or pre-pandemic healthy donors (M-HC-pre).

(A and B) Relative mechanical sensitivity (50% threshold of the mechanical force) to baseline (pre-injection) in the von Frey test.

(C and D) Relative heat sensitivity (latency) to baseline (pre-injection) in the Hargreaves assay.

Data points are shown as the mean +SD. Statistical testing used linear mixed-effects models with post hoc contrasts (R package emmeans) and Benjamini-Hochberg correction; asterisks denote significance vs. M-HCpre at the same time point. *$p < 0.05$, **$p < 0.01$.

to long COVID pathogenesis.[8] Consistent with these observations, our cohort exhibited sustained IFN-I signatures at both time points, with IFN-β showing the greatest consistency. In contrast, IFN II (IFN-γ) levels were reduced at baseline (2022) but normalized at follow-up (2024), mirroring mixed findings reported for IFN-γ in long COVID.[28,49] The persistent elevation of IFN-Is strengthens the mechanistic rationale for the biomarker-based subgrouping strategy, especially given their implied role in long COVID pathogenesis and other post-viral syndromes. We also observed increased plasma GFAP, aligning with other reports of increased GFAP and neuroglial perturbations in long COVID patients.[33,50,51] Mechanistically, sustained peripheral IFN-I signaling may be transmitted through the choroid plexus, where IFN-I gene expression persists after SARS-CoV-2 infection even in the absence of detectable viral RNA,[52] providing a potential route by which peripheral immune dysregulation could influence central nervous system glia and cognition.[53]

Our findings differ from studies reporting chronic systemic inflammation in long COVID, as we did not detect significant elevations in acute-phase proinflammatory cytokines. Many prior studies included individuals with severe acute COVID-19,[27,54,55] whereas our cohort consisted exclusively of patients with mild initial illness. Emerging evidence suggests the existence of both inflammatory and non-inflammatory long COVID endotypes, with persistent systemic inflammation observed predominantly in those who experienced severe acute disease.[56] Stratifying cohorts by acute disease severity may, therefore, help reconcile these discrepant findings.

Infections commonly elicit autoantibodies that alter signaling agonistically or antagonistically,[57,58] as observed in SARS-CoV-2,[59] influenza,[60] West Nile virus,[61] and Epstein-Barr virus.[62] Pathogenic IgG has been implicated in pain-related post-infection syndromes such as fibromyalgia and ME/CFS, with pathogenic IgG targeting glia and other antigens, including catalytic antibodies to myelin basic protein and β2-adrenergic receptors.[63,64] Additionally, in post-treatment Lyme disease syndrome, neural autoreactivity is enriched relative to healthy controls.[65] Our proteome-scale mapping identified an enriched/dense autoreactive repertoire in long COVID with subgroup-specific targets that plausibly map to clinical phenotypes, further supporting viral-induced autoimmunity.

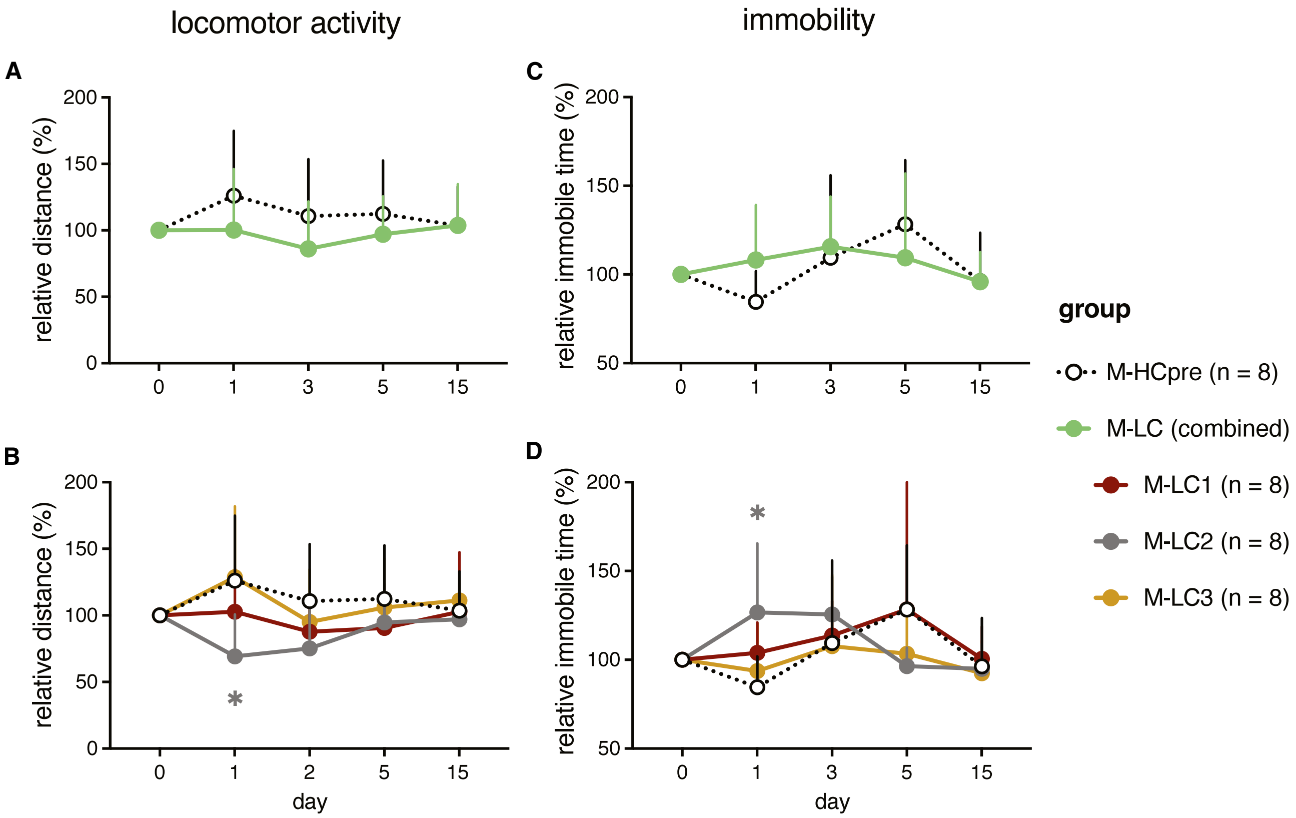

Figure 4. Passive transfer of long COVID IgG reduces locomotor activity in mice

Open field test of mice (*n* = 8 mice/group). Mice received a single intraperitoneal injection of pooled total human IgG from long COVD patient subgroups (M-LC1/2/3) or pre-pandemic healthy donors (M-HCpre).

(A and B) Relative walking distance to baseline (pre-injection) in the open field test.

(C and D) Relative immobility (time) to baseline (pre-injection) in the open field test.

Data points are shown as the mean +SD. Statistical testing used linear mixed-effects models with post hoc contrasts (R package emmeans) and Benjamini-Hochberg correction; asterisks denote significance vs. M-HCpre at the same time point. $^*p < 0.05$.

Our work identified putative mechanistic links by subgroup. The LC-1 subgroup showed elevated plasma GFAP and IgG reactivity against the astrocytic marker ALDH1L1, suggesting potential astrocyte activation, a process implicated in nerve injury and chronic pain.[66] Beyond astrocytes, FAP is expressed in fibroblasts and epidermal keratinocytes[67,68] and LC-1 autoantibodies were enriched for keratin targets. Because keratinocytes form synapse-like contacts with intraepidermal nerve fibers and modulate nociceptor activity,[69–71] this raises the possibility that LC-1 IgG could sensitize nociceptors through peripheral mechanisms. MMP-1, the most elevated plasma protein in LC-1, can also promote pain signaling via PAR1 activation.[72,73] Together, these observations point to a plausible but still speculative mechanism in which LC-1 IgG may induce mechanical hypersensitivity through combined peripheral and central pathways, with elevated GFAP reflecting glial and/or epithelial involvement.

The LC-2 subgroup exhibited plasma enrichment of skeletal and cardiac muscle-related proteins (e.g., TTN) and showed anti-IFNA1 IgG reactivity. Despite the presence of anti-IFNA1 IgG, IFN-β and its responding chemokine CXCL10 were elevated in these patients. Although anti-IFN-I autoantibodies during acute COVID-19 are typically neutralizing,[74,75] non-neutralizing or even stabilizing effects have also been described.[74,76–79] In mice receiving LC-2 IgG (M-LC2), plasma type-I IFN and CXCL10 levels were similar to other groups at 15 days post-injection. These findings suggest that the detected anti-IFNA1 IgGs are likely to be non-neutralizing or of low affinity and unlikely to explain either patient symptoms or mouse behavioral effects. Instead, persistent IFN-I signaling, as seen in chronic viral infections and autoimmune, can itself promote anti-IFN antibody formation,[77] shape the IgG repertoire and effector functions,[80] and influence central nervous system glia and cognition.[52,53] Interestingly, follow-up LC-2 IgGs collected 2 years later induced mechanical hypersensitivity, coinciding with rising GFAP and NFL levels approaching those of LC-1. This temporal convergence between glial injury markers and pain behavior suggests a possible evolution of the LC-2 endotype, in which sustained type-I IFN-biased immunity or progressive maturation of the IgG repertoire could increasingly engage glial nociceptor circuits. Additionally, future studies with larger cohorts or in larger-animal models will be needed to clarify the functional relevance of the modest locomotor phenotype.

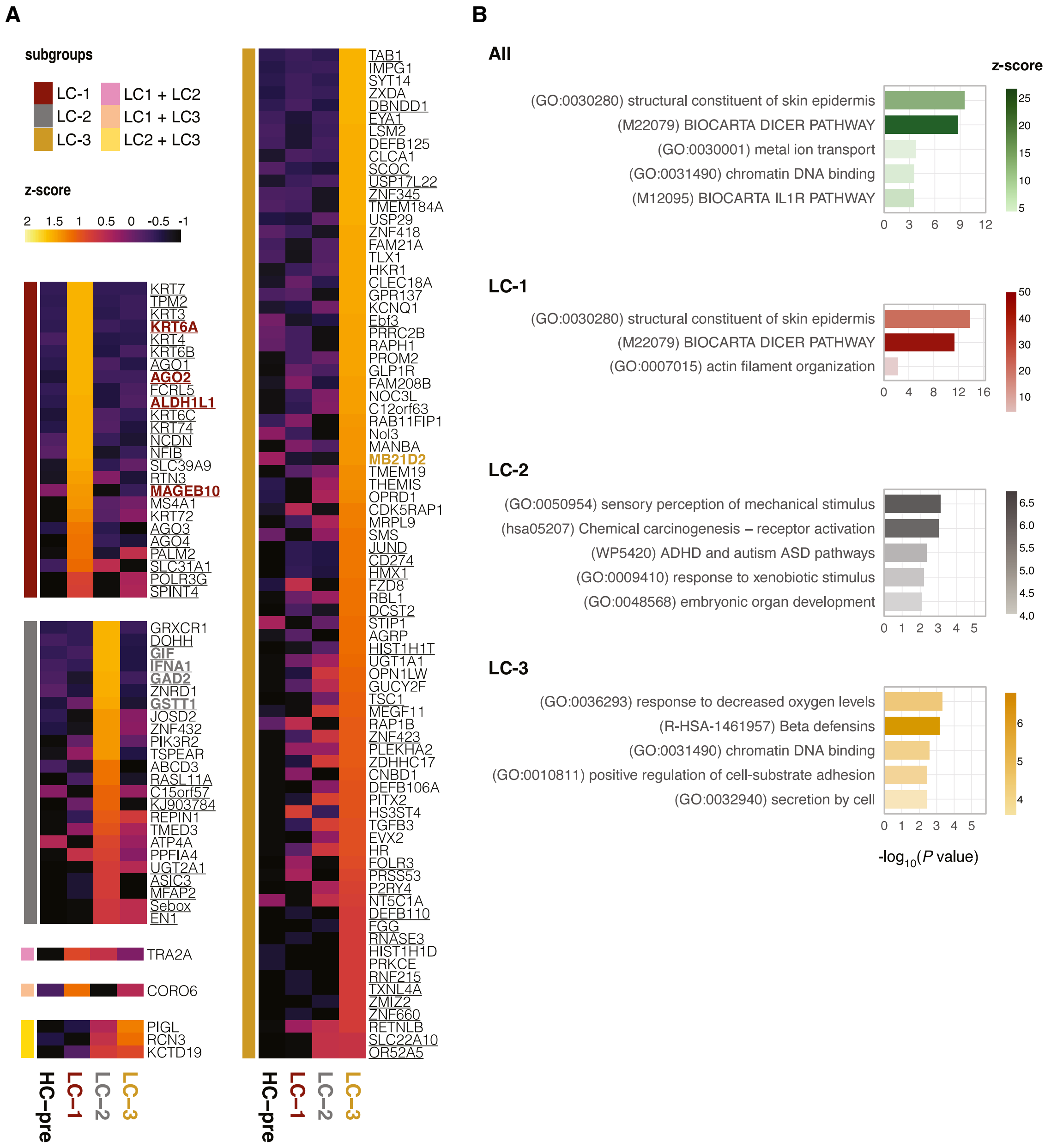

Figure 5. Autoantibody profiling of pooled IgG reveals subgroup-linked targets and persistence

(A) HuProt protein arrays profiled 2022 baseline plasma. LC-elevated autoantigens (n = 134) were called at 2-fold higher than healthy control intensity and above the on-array 1 μg/mL IgG standard. Heatmap shows Z scores; row color denotes the subgroup with highest reactivity (subgroup specificity is called if signal intensity is more than 1.5 times the next highest). unlined: >4-fold intensity relative to HC-pre

(B) Pathway analysis of LC-elevated and subgroup-mapped targets highlights epithelial structure, RNA silencing/Dicer, IL1R, sensory/receptor activation, and hypoxia/β-defensin programs.

LC-3 plasma was enriched for leukocyte activation proteins and signatures associated with multiple organs. Among the top increases was EIF5A, whose inhibition reduces firing of human induced pluripotent stem cell-derived neurons and prevents mechanical hypersensitivity.[81] Autoantibodies in LC-3 were enriched for proteins involved in metabolic processes. Notably, anti-MB21D2 targets a positive regulator of the cGAS-STING pathway in anti-viral IFN responses.[82] This aligns with comparatively lower IFN-I levels observed in LC-3, implying pathway-specific modulation of antiviral signaling mechanisms.

Interestingly, the plasma proteomics and autoantibody profiles in each LC subgroup point to indirectly related yet non-overlapping aspects of pathology. For example, LC-1 showed a plasma proteome enriched for nervous system-derived proteins suggestive of glial/neuronal stress, while its autoantibody profile was not dominated by classical neuro-antigens except

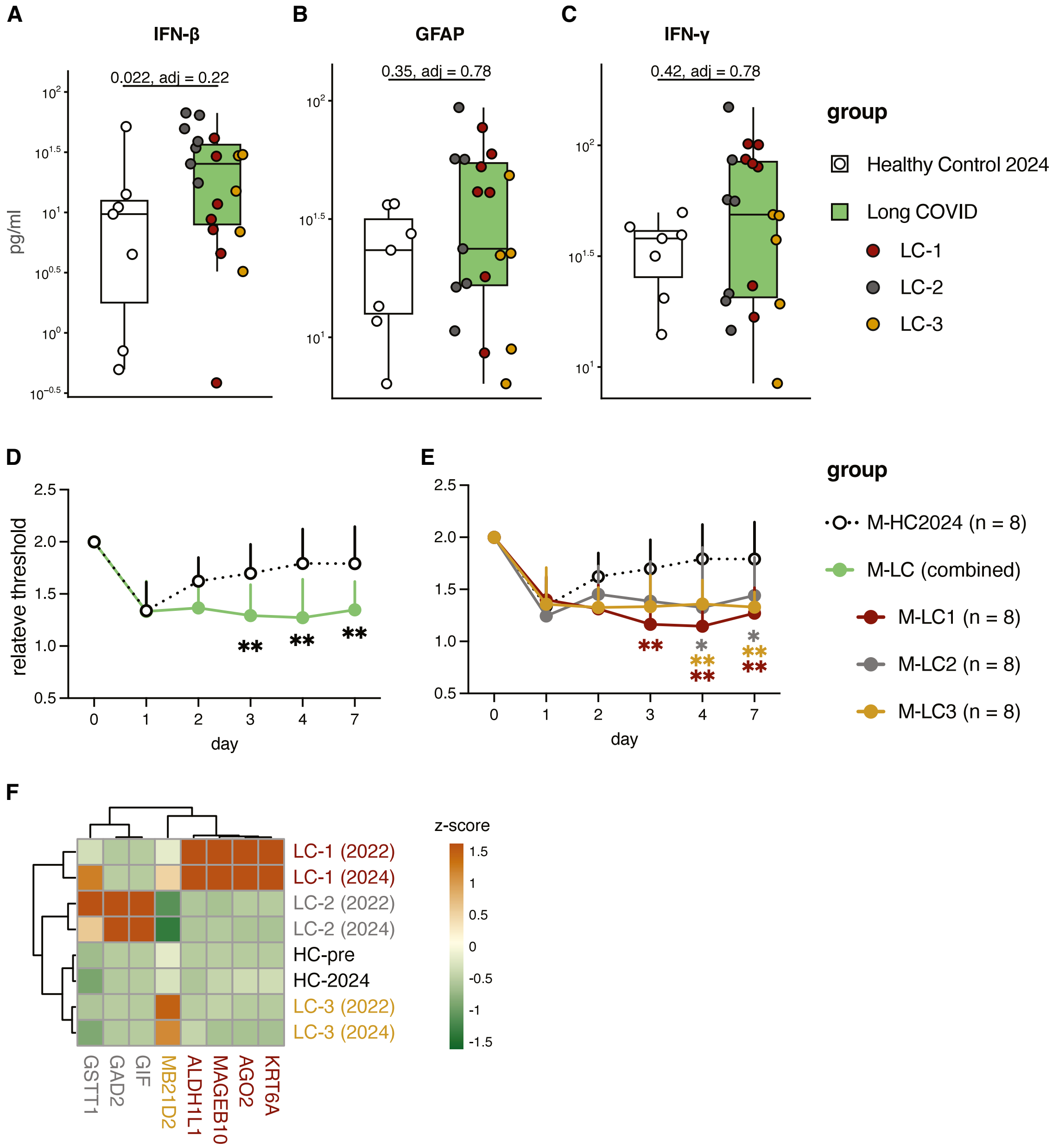

Figure 6. Two-year follow-up plasma biomarkers, autoantibodies, and replicated IgG-induced hypersensitivity

(A–C) Targeted quantitative measurements using Meso Scale Discovery of follow-up plasma from long COVID (LC; green, $n = 19$) vs. a new cohort of post-SARS-CoV-2, non-LC healthy controls (HC-2024; white, $n = 7$). Numbers above brackets are p values with Benjamini-Hochberg (BH)-adjusted p values in parentheses from linear models adjusted for age, sex, and days since infection. Individual LC donors are colored by subgroup: LC-1, red; LC-2, gray; LC-3, yellow.

(D and E) Passive transfer using follow-up pooled total IgG from LC donors (combined or endotype-specific pools; M-LC, M-LC1/2/3) and HC-2024 (M-HC2024). C57BL/6 mice ($n = 8$ mice/group) received a single intraperitoneal dose (260 mg/kg). Relative mechanical threshold (von Frey) is normalized to each mouse's baseline (days 1–7). Points/lines show mean +SD. Statistics used linear mixed-effects models with post hoc contrasts (emmeans) and BH correction; asterisks denote significance vs. M-HC2024 at the same time point. *$p < 0.05$, **$p < 0.01$.

(F) Heatmap of validated LC-associated autoantibodies showing Z score elevations at baseline (2022) and at 2-year follow-up (2024), indicating persistence. Columns are subgroup/time point; rows include representative antigens. Warmer colors reflect higher reactivity (Z scored within antigen). Label colors indicate subgroup: LC-1, red; LC-2, gray; LC-3, yellow; healthy controls (HC-pre, HC-2022) are black. HC-pre, pre-pandemic healthy controls; HC-2022, post-COVID controls from 2022; HC-2024, post-COVID controls for the follow-up transfer.

astrocytic marker ALDH1L1. Instead, the prominent LC-1 autoantigens included AGO protein family, which is known for their function in RNA interference. Yet, without known pathogenic mechanisms, anti-AGO IgGs have been linked to autoimmune neurologic diseases.[83] Similarly, LC-2's plasma proteome shows high levels of skeletal/cardiac muscle-associated proteins, while its key autoantibodies do not directly target muscle fibers. The presence of anti-GAD2 antibodies in LC-2 offers a potential mechanistic bridge between motor and nociceptive features, given prior associations of anti-GAD2 with neuromuscular and pain syndromes.[84,85] These indirect but associated links between plasma profile and autoantibody profile suggest that proteomic biomarkers and autoantibody profiles represent different phases or layers of long COVID pathology, underscoring a complex, multi-layered disease process rather than a one-to-one correspondence between tissue injury and autoimmunity.

The subgroup tissue-enrichment patterns observed in our proteomic and autoantibody pathway analysis (LC-1, nervous system; LC-2, muscle; LC-3, metabolic tissues) suggest potential target organs for circulating autoantibodies. Under our conditions (single intraperitoneal dose; day 1 and day 15 readouts), transferred hIgG deposition accumulated in all examined tissues in mice across all groups, with no discernible differences vs. controls and no clear separation among LC subgroups. This suggests that tissue deposition is a generic property of circulating hIgG in this mouse assay, rather than long COVID (subgroup) specific. The absence of disease-/subgroup-specific tissue staining patterns limits the interpretability of co-staining approaches and precludes meaningful validation using tissues from specific antigen-knockout mice. This contrasts with previous reports describing patient-specific tissue accumulation and transfer-induced hypersensitivity in fibromyalgia[24] and long COVID.[86] Notably, IgG from both healthy control cohorts (HC-pre and HC-2024) also showed widespread tissue deposition. These differences may reflect the pooling of donor IgG in our study, which could dilute high-affinity or pathogenic specificities present in individual patients or differences in IgG composition, such as subclass distribution, Fc glycosylation, or complement engagement, that influence tissue retention.

If validated, our findings could open avenues for precision immunotherapy in long COVID. Therapies that remove or neutralize pathogenic IgG, such as immunoadsorption, plasmapheresis, intravenous immunoglobulin, or FcRn blockade, may provide symptomatic relief in antibody-driven endotypes. Longer-lasting strategies that deplete autoreactive B or plasma cells (e.g., anti-CD38 or anti-CD20 antibodies, or B cell-directed CAR-T/NK approaches) could further be explored for refractory cases. Elucidating antigenic targets using knockout models and defining downstream effector mechanisms may ultimately enable stratified, endotype-specific interventions aimed at durable immune rebalancing.

In conclusion, our study demonstrates that passive transfer of IgG from long COVID patients to mice induces reproducible sensory hypersensitivity, paralleling persistent IFN-I and neuro-injury signatures, as well as subgroup-specific autoreactivities that remain stable over time. The durability of these effects across two time points, separated by two years, strengthens the evidence for a causal contribution of autoantibodies to long COVID pathogenesis and highlights the importance of longitudinal immunophenotyping. This murine model establishes a foundation for mechanistic dissection and the development of endotype-informed therapeutic strategies.

Limitations of the study

Our study has several limitations. While limited by its single-center design and lack of a prespecified power calculation, our study provides evidence for a pathogenic role of IgG in long COVID and merits replication in larger, adequately powered, multi-center cohorts. Nonetheless, independent preliminary studies have similarly reported that transfer of long COVID IgG can induce symptomatology in mice,[86–88] supporting a causal role.

We prioritized cytokine and neuro-injury markers for subgrouping based on mechanistic relevance and reproducibility. Studies with larger cohorts, denser longitudinal sampling, and unbiased proteomics-driven clustering may reveal additional or partially overlapping endotypes. While pooled IgG transfer reduces supply constraints and variability, it may mask donor-specific effects, dilute pathogenic autoantibodies, and complicate symptom attribution. Because IgG was pooled within each subgroup, donor-level molecular profiles cannot be directly linked to behavioral outcomes. Future studies using one-donor-to-one- or multiple-mouse designs will be essential to resolve these associations.

Although IgG was purified using well-established methods and quality controlled, the potential contribution of residual non-IgG components (e.g., from IgG-antigen complexes) cannot be fully excluded. Additionally, our behavioral assessments focused primarily on nociception and spontaneous activity and did not capture fatigue, exertional intolerance, dysautonomia, or cognitive dysfunction. Cross-species constraints, such as hIgG-murine FcγR interactions and antigen orthology, may also influence pathogenic effects or underrepresent tissue/cell targeting.

In the first transfer experiment, most patient participants had been vaccinated prior to sampling, whereas the controls were not. Importantly, in our follow-up experiment, we used post-pandemic controls (exposed and vaccinated), and their IgG still did not induce the overt pain phenotype seen with long COVID IgG, suggesting vaccination alone is unlikely to explain the transfer effects. Nevertheless, vaccination can shape IgG repertoires and Fc glycosylation,[89] and immune-mediated syndromes following vaccination have been reported.[90,91] Future studies should, therefore, match cohorts on vaccination status and timing and incorporate functional IgG analyses, including subclass distribution and Fc glycoform profiling, to disentangle infection-driven from vaccine-modulated immune effects.

Collectively, these considerations reflect deliberate design choices made to prioritize mechanistic clarity, longitudinal stability, and translational feasibility in an initial human-to-mouse transfer model, and they define a clear roadmap for iterative refinement in future studies.

RESOURCE AVAILABILITY

Lead contact

Further information and requests for resources and reagents should be directed to and will be fulfilled by the lead contact, Niels Eijkelkamp (n.eijkelkamp@umcutrecht.nl).

Materials availability

This study did not generate new unique reagents.

Data and code availability

- All data reported in this paper will be shared by the lead contact upon request. HuProt autoantibody intensity of the full array is in the supplementary table.
- This paper does not report original code. All analyses were performed using R (RStudio) with publicly available, well-established R packages and did not involve novel algorithms or non-standard analytical workflows. The R scripts used in this study are available at https://github.com/bappelman-AmsterdamUMC/R-CODE—IgG-transfer_LC and https://github.com/oliverch77/LongCOVID_IgG_transfer_2022.
- Any additional information required to reanalyze the data reported in this paper is available from the lead contact upon request.

CONSORTIA

The members of the Amsterdam UMC COVID-19 biobank are Michiel van Agtmael, Anne Geke Algera, Brent Appelman, Floor van Baarle, Martijn Beudel, Harm Jan Bogaard, Marije Bomers, Peter Bonta, Lieuwe Bos, Michela Botta, Justin de Brabander, Godelieve de Bree, Sanne de Bruin, Marianna Bugiani, Esther Bulle, David T.P. Buis, Osoul Chouchane, Alex Cloherty, Mirjam Dijkstra, Dave A. Dongelmans, Romein W.G. Dujardin, Paul Elbers, Lucas Fleuren, Suzanne Geerlings, Theo Geijtenbeek, Armand Girbes, Bram Goorhuis, Martin P. Grobusch, Laura Hagens, Jorg Hamann, Vanessa Harris, Robert Hemke, Sabine M. Hermans, Leo Heunks, Markus Hollmann, Janneke Horn, Joppe W. Hovius, Katja de Jong, Menno D. de Jong, Rutger Koning, Bregje Lemkes, Endry H.T. Lim, Niels van Mourik, Jeaninne Nellen, Esther J. Nossent5, Sabine Olie, Frederique Paulus, Edgar Peters, Dan A.I. Pina-Fuentes, Tom van der Poll, Bennedikt Preckel, Jan M. Prins, Jorinde Raasveld, Tom Reijnders, Maurits C.F.J. de Rotte, Michiel Schinkel, Marcus J. Schultz, Femke A.P. Schrauwen, Alex Schuurman, Jaap Schuurmans, Kim Sigaloff, Marleen A. Slim, Patrick Smeele, Marry Smit, Cornelis S. Stijnis, Willemke Stilma, Charlotte Teunissen, Patrick Thoral, Anissa M Tsonas, Pieter R. Tuinman, Marc van der Valk, Denise Veelo, Carolien Volleman, Heder de Vries, Lonneke A. Vught, Michèle van Vugt, Dorien Wouters, A.H. Koos Zwinderman, Matthijs C. Brouwer, W. Joost Wiersinga, Alexander P.J. Vlaar, and Diederik van de Beek.

ACKNOWLEDGMENTS

We would like to thank all the patients and the staff of the Amsterdam UMC post-COVID clinic for their contribution. This work was supported by the Patient-Led Research Collaborative of Long COVID (grant ID: C1) and the Netherlands Organisation for Health Research and Development (ZonMw, grant number 10430142210001). N.E. is supported by the Netherlands Organisation for Scientific Research (NWO) (Vici 09150182210016). H.-J.C. is supported by the Stichting Long-COVID consortium grant, the Netherlands Organisation for Health Research and Development (ZonMw, grant number 10430172310009). The funders had no role in study design, data collection, and analysis; decision to publish; or preparation of the manuscript.

AUTHOR CONTRIBUTIONS

Conceptualization, J.d.D. and N.E.; cohort, B.A., M.v.V., M.K.B., A.H.A.L., and AUMC COVID-19 biobank; methodology, H.-J.C., B.A., H.L.D.M.W., J.P., M.L., and A.B.; investigation, H.-J.C., B.A., H.L.D.M.W., J.P., C.E.G., A.B., E.S., S.V., P.S.S.R., N.K., S.V.G., and W.A.M.; formal analysis, H.-J.C., B.A., H.L.D.M.W., J.P., and A.B.; original draft, B.A. and H.-J.C.; review and editing, J.d.D., N.E., W.J.W., H.-J.C., B.A., H.L.D.M.W., P.S.S.R., J.P., A.B., and G.V.; funding, B.A., J.d.D., and N.E.

DECLARATION OF INTERESTS

The authors declare no competing interest.

STAR★METHODS

Detailed methods are provided in the online version of this paper and include the following:

- KEY RESOURCES TABLE
- EXPERIMENTAL MODEL AND STUDY PARTICIPANT DETAILS
  - Study population
  - Mouse model and behavioral assays
- METHOD DETAILS
  - Meso Scale Discovery (MSD) multiplex assay
  - OLINK proteomics
  - Human IgG purification
  - Immunohistochemistry
  - Autoantibody analysis by HuProt
  - Mouse plasma biomarker quantification by Luminex
  - Luminex-based autoantibody validation
- QUANTIFICATION AND STATISTICAL ANALYSIS

SUPPLEMENTAL INFORMATION

Supplemental information can be found online at https://doi.org/10.1016/j.xcrm.2026.102693.

Received: July 19, 2024
Revised: October 20, 2025
Accepted: February 19, 2026
Published: March 24, 2026

REFERENCES

1. WHO (2024). WHO Coronavirus (COVID-19) Dashboard (World Health Organization).
2. Ballering, A.V., van Zon, S.K.R., Olde Hartman, T.C., and Rosmalen, J.G.M.; Lifelines Corona Research Initiative (2022). Persistence of somatic symptoms after COVID-19 in the Netherlands: an observational cohort study. Lancet *400*, 452–461.
3. Davis, H.E., McCorkell, L., Vogel, J.M., and Topol, E.J. (2023). Long COVID: major findings, mechanisms and recommendations. Nat. Rev. Microbiol. *21*, 133–146.
4. Haslam, A., Olivier, T., and Prasad, V. (2023). The definition of long COVID used in interventional studies. Eur. J. Clin. Invest. *53*, e13989.
5. Mehandru, S., and Merad, M. (2022). Pathological sequelae of long-haul COVID. Nat. Immunol. *23*, 194–202.
6. Choutka, J., Jansari, V., Hornig, M., and Iwasaki, A. (2022). Unexplained post-acute infection syndromes. Nat. Med. *28*, 911–923.
7. Appelman, B., Charlton, B.T., Goulding, R.P., Kerkhoff, T.J., Breedveld, E.A., Noort, W., Offringa, C., Bloemers, F.W., van Weeghel, M., Schomakers, B.V., et al. (2024). Muscle abnormalities worsen after post-exertional malaise in long COVID. Nat. Commun. *15*, 17.
8. Wong, A.C., Devason, A.S., Umana, I.C., Cox, T.O., Dohnalová, L., Litichevskiy, L., Perla, J., Lundgren, P., Etwebi, Z., Izzo, L.T., et al. (2023). Serotonin reduction in post-acute sequelae of viral infection. Cell *186*, 4851–4867.e20.
9. Cervia-Hasler, C., Brüningk, S.C., Hoch, T., Fan, B., Muzio, G., Thompson, R.C., Ceglarek, L., Meledin, R., Westermann, P., Emmenegger, M., et al.

CellPress
OPEN ACCESS

(2024). Persistent complement dysregulation with signs of thromboinflammation in active Long Covid. Science *383*, eadg7942.

10. Altmann, D.M., Whettlock, E.M., Liu, S., Arachchillage, D.J., and Boyton, R.J. (2023). The immunology of long COVID. Nat. Rev. Immunol. *23*, 618–634.

11. Yang, C., Zhao, H., Espín, E., and Tebbutt, S.J. (2023). Association of SARS-CoV-2 infection and persistence with long COVID. Lancet Respir. Med. *11*, 504–506.

12. Díaz-Resendiz, K.J.G., Benitez-Trinidad, A.B., Covantes-Rosales, C.E., Toledo-Ibarra, G.A., Ortiz-Lazareno, P.C., Girón-Pérez, D.A., Bueno-Durán, A.Y., Pérez-Díaz, D.A., Barcelos-García, R.G., and Girón-Pérez, M.I. (2022). Loss of mitochondrial membrane potential (DeltaPsi(m)) in leucocytes as post-COVID-19 sequelae. J. Leukoc. Biol. *112*, 23–29.

13. Guo, L., Appelman, B., Mooij-Kalverda, K., Houtkooper, R.H., van Weeghel, M., Vaz, F.M., Dijkhuis, A., Dekker, T., Smids, B.S., Duitman, J.W., et al. (2023). Prolonged indoleamine 2,3-dioxygenase-2 activity and associated cellular stress in post-acute sequelae of SARS-CoV-2 infection. EBioMedicine *94*, 104729.

14. Mendes de Almeida, V., Engel, D.F., Ricci, M.F., Cruz, C.S., Lopes, Í.S., Alves, D.A., d' Auriol, M., Magalhães, J., Machado, E.C., Rocha, V.M., et al. (2023). Gut microbiota from patients with COVID-19 cause alterations in mice that resemble post-COVID symptoms. Gut Microbes *15*, 2249146.

15. Needham, E.J., Ren, A.L., Digby, R.J., Norton, E.J., Ebrahimi, S., Outtrim, J.G., Chatfield, D.A., Manktelow, A.E., Leibowitz, M.M., Newcombe, V.F.J., et al. (2022). Brain injury in COVID-19 is associated with dysregulated innate and adaptive immune responses. Brain *145*, 4097–4107.

16. Wang, E.Y., Mao, T., Klein, J., Dai, Y., Huck, J.D., Jaycox, J.R., Liu, F., Zhou, T., Israelow, B., Wong, P., et al. (2021). Diverse functional autoantibodies in patients with COVID-19. Nature *595*, 283–288.

17. Su, Y., Yuan, D., Chen, D.G., Ng, R.H., Wang, K., Choi, J., Li, S., Hong, S., Zhang, R., Xie, J., et al. (2022). Multiple early factors anticipate post-acute COVID-19 sequelae. Cell *185*, 881–895.e20.

18. Hoepel, W., Chen, H.J., Geyer, C.E., Allahverdiyeva, S., Manz, X.D., de Taeye, S.W., Aman, J., Mes, L., Steenhuis, M., Griffith, G.R., et al. (2021). High titers and low fucosylation of early human anti-SARS-CoV-2 IgG promote inflammation by alveolar macrophages. Sci. Transl. Med. *13*, eabf8654.

19. Dobrowolska, K., Zarębska-Michaluk, D., Poniedziałek, B., Jaroszewicz, J., Flisiak, R., and Rzymski, P. (2023). Overview of autoantibodies in COVID-19 convalescents. J. Med. Virol. *95*, e28864.

20. Muri, J., Cecchinato, V., Cavalli, A., Shanbhag, A.A., Matkovic, M., Biggiogero, M., Maida, P.A., Moritz, J., Toscano, C., Ghovehoud, E., et al. (2023). Autoantibodies against chemokines post-SARS-CoV-2 infection correlate with disease course. Nat. Immunol. *24*, 604–611.

21. Wallukat, G., Hohberger, B., Wenzel, K., Fürst, J., Schulze-Rothe, S., Wallukat, A., Hönicke, A.S., and Müller, J. (2021). Functional autoantibodies against G-protein coupled receptors in patients with persistent Long-COVID-19 symptoms. J. Transl. Autoimmun. *4*, 100100.

22. Achleitner, M., Steenblock, C., Dänhardt, J., Jarzebska, N., Kardashi, R., Kanczkowski, W., Straube, R., Rodionov, R.N., Bornstein, N., Tselmin, S., et al. (2023). Clinical improvement of Long-COVID is associated with reduction in autoantibodies, lipids, and inflammation following therapeutic apheresis. Mol. Psychiatry *28*, 2872–2877.

23. Bodansky, A., Wang, C.Y., Saxena, A., Mitchell, A., Kung, A.F., Takahashi, S., Anglin, K., Huang, B., Hoh, R., Lu, S., et al. (2023). Autoantigen profiling reveals a shared post-COVID signature in fully recovered and long COVID patients. JCI Insight *8*, e169515.

24. Goebel, A., Krock, E., Gentry, C., Israel, M.R., Jurczak, A., Urbina, C.M., Sandor, K., Vastani, N., Maurer, M., Cuhadar, U., et al. (2021). Passive transfer of fibromyalgia symptoms from patients to mice. J. Clin. Investig. *131*, e144201.

25. Dawes, J.M., Weir, G.A., Middleton, S.J., Patel, R., Chisholm, K.I., Pettingill, P., Peck, L.J., Sheridan, J., Shakir, A., Jacobson, L., et al. (2018). Immune or Genetic-Mediated Disruption of CASPR2 Causes Pain Hypersensitivity Due to Enhanced Primary Afferent Excitability. Neuron *97*, 806–822.e10.

26. Karbalaeimahdi, M., Farajnia, S., Bargahi, N., Ghadiri-Moghaddam, F., Rasouli Jazi, H.R., Bakhtiari, N., Ghasemali, S., and Zarghami, N. (2023). The Role of Interferons in Long Covid Infection. J. Interferon Cytokine Res. *43*, 65–76.

27. Schultheiß, C., Willscher, E., Paschold, L., Gottschick, C., Klee, B., Henkes, S.S., Bosurgi, L., Dutzmann, J., Sedding, D., Frese, T., et al. (2022). The IL-1beta, IL-6, and TNF cytokine triad is associated with post-acute sequelae of COVID-19. Cell Rep. Med. *3*, 100663.

28. Williams, E., Martins, T.B., Hill, H.R., Coiras, M., Shah, K.S., Planelles, V., and Spivak, A.M. (2022). Plasma cytokine levels reveal deficiencies in IL-8 and gamma interferon in Long-COVID. Preprint at medRxiv. https://doi.org/10.1101/2022.10.03.22280661.

29. Visser, D., Golla, S.S.V., Verfaillie, S.C.J., Coomans, E.M., Rikken, R.M., van de Giessen, E.M., den Hollander, M.E., Verveen, A., Yaqub, M., Barkhof, F., et al. (2022). Long COVID is associated with extensive *in-vivo* neuroinflammation on $[^{18}F]$DPA-714 PET. medRxiv. https://doi.org/10.1101/2022.06.02.22275916.

30. Braga, J., Lepra, M., Kish, S.J., Rusjan, P.M., Nasser, Z., Verhoeff, N., Vasdev, N., Bagby, M., Boileau, I., Husain, M.I., et al. (2023). Neuroinflammation After COVID-19 With Persistent Depressive and Cognitive Symptoms. JAMA Psychiatry *80*, 787–795.

31. Song, W.J., Hui, C.K.M., Hull, J.H., Birring, S.S., McGarvey, L., Mazzone, S.B., and Chung, K.F. (2021). Confronting COVID-19-associated cough and the post-COVID syndrome: role of viral neurotropism, neuroinflammation, and neuroimmune responses. Lancet Respir. Med. *9*, 533–544.

32. Del Valle, D.M., Kim-Schulze, S., Huang, H.H., Beckmann, N.D., Nirenberg, S., Wang, B., Lavin, Y., Swartz, T.H., Madduri, D., Stock, A., et al. (2020). An inflammatory cytokine signature predicts COVID-19 severity and survival. Nat. Med. *26*, 1636–1643.

33. Vrettou, C.S., Vassiliou, A.G., Keskinidou, C., Mourelatos, P., Asimakos, A., Spetsioti, S., Diamantopoulos, A., Jahaj, E., Antonoglou, A., Katsaounou, P., et al. (2024). A Prospective Study on Neural Biomarkers in Patients with Long-COVID Symptoms. J. Pers. Med. *14*, 313.

34. Gutman, E.G., Salvio, A.L., Fernandes, R.A., Duarte, L.A., Raposo-Vedovi, J.V., Alcaraz, H.F., Teixeira, M.A., Passos, G.F., de Medeiros, K.Q.M., Hammerle, M.B., et al. (2024). Long COVID: plasma levels of neurofilament light chain in mild COVID-19 patients with neurocognitive symptoms. Mol. Psychiatry *29*, 3106–3116.

35. Phetsouphanh, C., Darley, D.R., Wilson, D.B., Howe, A., Munier, C.M.L., Patel, S.K., Juno, J.A., Burrell, L.M., Kent, S.J., Dore, G.J., et al. (2022). Immunological dysfunction persists for 8 months following initial mild-to-moderate SARS-CoV-2 infection. Nat. Immunol. *23*, 210–216.

36. Santinelli, L., Gentilini Cacciola, E., Bortolani, L., Ridolfi, M., Maddaloni, L., Frasca, F., Fracella, M., Bugani, G., d'Ettorre, G., Mastroianni, C.M., et al. (2025). Long COVID and Type I IFN Signature in Working-Age Adults: A Cross-Sectional Study. Int. J. Mol. Sci. *26*, 9089.

37. Klein-Schneegans, A.S., Kuntz, L., Fonteneau, P., and Loor, F. (1989). Serum concentrations of IgM, IgG1, IgG2b, IgG3 and IgA in C57BL/6 mice and their congenics at the lpr (lymphoproliferation) locus. J. Autoimmun. *2*, 869–875.

38. Hargreaves, K., Dubner, R., Brown, F., Flores, C., and Joris, J. (1988). A new and sensitive method for measuring thermal nociception in cutaneous hyperalgesia. Pain *32*, 77–88.

39. Swarnakar, R., Jenifa, S., and Wadhwa, S. (2022). Musculoskeletal complications in long COVID-19: A systematic review. World J. Virol. *11*, 485–495.

40. Brooks, S.P., and Dunnett, S.B. (2009). Tests to assess motor phenotype in mice: a user's guide. Nat. Rev. Neurosci. *10*, 519–529.

41. Hamm, R.J., Pike, B.R., O'Dell, D.M., Lyeth, B.G., and Jenkins, L.W. (1994). The rotarod test: an evaluation of its effectiveness in assessing motor deficits following traumatic brain injury. J. Neurotrauma *11*, 187–196.
42. Bérubé, S., Kobayashi, T., Wesolowski, A., Norris, D.E., Ruczinski, I., Moss, W.J., and Louis, T.A. (2022). A pre-processing pipeline to quantify, visualize, and reduce technical variation in protein microarray studies. Proteomics *22*, e2100033.
43. Gregory-Flores, A., Bonet, I.J., Desaivre, S., Levine, J.D., McHardy, S.F., de Kraker, H.C., Clanton, N.A., LoCoco, P.M., Russell, N.M., Fleischer, C., et al. (2025). A small molecule PKCε inhibitor reduces hyperalgesia induced by paclitaxel or opioid withdrawal. JCI Insight *10*, e186805.
44. Fang, J., Wang, S., Zhou, J., Shao, X., Sun, H., Liang, Y., He, X., Jiang, Y., Liu, B., Jin, X., et al. (2021). Electroacupuncture Regulates Pain Transition Through Inhibiting PKCε and TRPV1 Expression in Dorsal Root Ganglion. Front. Neurosci. *15*, 685715.
45. Xu, Y.-R., and Lei, C.-Q. (2021). TAK1-TABs Complex: A Central Signalosome in Inflammatory Responses. Front. Immunol. *11*, 608976.
46. Wu, Q., and Liu, L. (2018). ORL(1) Activation Mediates a Novel ORL(1) Receptor Agonist SCH221510 Analgesia in Neuropathic Pain in Rats. J. Mol. Neurosci. *66*, 10–16.
47. Queiroz, M.A.F., Brito, W.R.D.S., Pereira, K.A.S., Pereira, L.M.S., Amoras, E.d.S.G., Lima, S.S., Santos, E.F.D., Costa, F.P.d., Sarges, K.M.L.d., Cantanhede, M.H.D., et al. (2024). Severe COVID-19 and long COVID are associated with high expression of STING, cGAS and IFN-α. Sci. Rep. *14*, 4974.
48. Fracella, M., Mancino, E., Nenna, R., Virgillito, C., Frasca, F., D'Auria, A., Sorrentino, L., Petrarca, L., La Regina, D., Matera, L., et al. (2024). Age-related transcript changes in type I interferon signaling in children and adolescents with long COVID. Eur. J. Immunol. *54*, e2350682.
49. Krishna, B.A., Lim, E.Y., Metaxaki, M., Jackson, S., Mactavous, L., NIHR BioResource; Lyons, P.A., Doffinger, R., Bradley, J.R., Smith, K.G.C., et al. (2024). Spontaneous, persistent, T cell-dependent IFN-γ release in patients who progress to Long Covid. Sci. Adv. *10*, eadi9379.
50. Bark, L., Larsson, I.M., Wallin, E., Simrén, J., Zetterberg, H., Lipcsey, M., Frithiof, R., Rostami, E., and Hultström, M. (2023). Central nervous system biomarkers GFAp and NfL associate with post-acute cognitive impairment and fatigue following critical COVID-19. Sci. Rep. *13*, 13144.
51. Telser, J., Grossmann, K., Weideli, O.C., Hillmann, D., Aeschbacher, S., Wohlwend, N., Velez, L., Kuhle, J., Maleska, A., Benkert, P., et al. (2023). Concentrations of Serum Brain Injury Biomarkers Following SARS-CoV-2 Infection in Individuals with and without Long-COVID-Results from the Prospective Population-Based COVI-GAPP Study. Diagnostics *13*, 2167.
52. Suzzi, S., Tsitsou-Kampeli, A., and Schwartz, M. (2023). The type I interferon antiviral response in the choroid plexus and the cognitive risk in COVID-19. Nat. Immunol. *24*, 220–224.
53. Vavougios, G.D., Tseriotis, V.S., Liampas, A., Mavridis, T., de Erausquin, G.A., and Hadjigeorgiou, G. (2024). Type I interferon signaling, cognition and neurodegeneration following COVID-19: update on a mechanistic pathogenetic model with implications for Alzheimer's disease. Front. Hum. Neurosci. *18*, 1352118.
54. Etter, M.M., Martins, T.A., Kulsvehagen, L., Pössnecker, E., Duchemin, W., Hogan, S., Sanabria-Diaz, G., Müller, J., Chiappini, A., Rychen, J., et al. (2022). Severe Neuro-COVID is associated with peripheral immune signatures, autoimmunity and neurodegeneration: a prospective cross-sectional study. Nat. Commun. *13*, 6777.
55. Woodruff, M.C., Bonham, K.S., Anam, F.A., Walker, T.A., Faliti, C.E., Ishii, Y., Kaminski, C.Y., Ruunstrom, M.C., Cooper, K.R., Truong, A.D., et al. (2023). Chronic inflammation, neutrophil activity, and autoreactivity splits long COVID. Nat. Commun. *14*, 4201.
56. Maciel, A.B.S., Pinto, A.S., Maia Silva, B., Goulart, C.L., Silva, L.F.A., Chaves, A.S., Mouta, G.S., Sato, C.M.S., Valente, J., Mwangi, V.I., et al. (2024). Inflammatory discoveries two years after acute severe COVID-19: a longitudinal biomarker profile assessment in long COVID individuals in the Brazilian Amazon. Front. Immunol. *15*, 1520193.
57. Johnson, D., and Jiang, W. (2023). Infectious diseases, autoantibodies, and autoimmunity. J. Autoimmun. *137*, 102962.
58. Ludwig, R.J., Vanhoorelbeke, K., Leypoldt, F., Kaya, Z., Bieber, K., McLachlan, S.M., Komorowski, L., Luo, J., Cabral-Marques, O., Hammers, C.M., et al. (2017). Mechanisms of Autoantibody-Induced Pathology. Front. Immunol. *8*, 603.
59. Franke, C., Boesl, F., Goereci, Y., Gerhard, A., Schweitzer, F., Schroeder, M., Foverskov-Rasmussen, H., Heine, J., Quitschau, A., Kandil, F.I., et al. (2023). Association of cerebrospinal fluid brain-binding autoantibodies with cognitive impairment in post-COVID-19 syndrome. Brain Behav. Immun. *109*, 139–143.
60. Zhang, Q., Pizzorno, A., Miorin, L., Bastard, P., Gervais, A., Le Voyer, T., Bizien, L., Manry, J., Rosain, J., Philippot, Q., et al. (2022). Autoantibodies against type I IFNs in patients with critical influenza pneumonia. J. Exp. Med. *219*, e20220514.
61. Gervais, A., Rovida, F., Avanzini, M.A., Croce, S., Marchal, A., Lin, S.C., Ferrari, A., Thorball, C.W., Constant, O., Le Voyer, T., et al. (2023). Autoantibodies neutralizing type I IFNs underlie West Nile virus encephalitis in approximately 40% of patients. J. Exp. Med. *220*, e20230661.
62. Garzelli, C., Taub, F.E., Scharff, J.E., Prabhakar, B.S., Ginsberg-Fellner, F., and Notkins, A.L. (1984). Epstein-Barr virus-transformed lymphocytes produce monoclonal autoantibodies that react with antigens in multiple organs. J. Virol. *52*, 722–725.
63. Fanton, S., Menezes, J., Krock, E., Sandström, A., Tour, J., Sandor, K., Jurczak, A., Hunt, M., Baharpoor, A., Kadetoff, D., et al. (2023). Anti-satellite glia cell IgG antibodies in fibromyalgia patients are related to symptom severity and to metabolite concentrations in thalamus and rostral anterior cingulate cortex. Brain Behav. Immun. *114*, 371–382.
64. Jensen, M.A., Dafoe, M.L., Wilhelmy, J., Cervantes, L., Okumu, A.N., Kipp, L., Nemat-Gorgani, M., and Davis, R.W. (2024). Catalytic Antibodies May Contribute to Demyelination in Myalgic Encephalomyelitis/Chronic Fatigue Syndrome. Biochemistry *63*, 9–18.
65. Chandra, A., Wormser, G.P., Klempner, M.S., Trevino, R.P., Crow, M.K., Latov, N., and Alaedini, A. (2010). Anti-neural antibody reactivity in patients with a history of Lyme borreliosis and persistent symptoms. Brain Behav. Immun. *24*, 1018–1024.
66. Li, T., Chen, X., Zhang, C., Zhang, Y., and Yao, W. (2019). An update on reactive astrocytes in chronic pain. J. Neuroinflammation *16*, 140.
67. Hainfellner, J.A., Voigtländer, T., Ströbel, T., Mazal, P.R., Maddalena, A.S., Aguzzi, A., and Budka, H. (2001). Fibroblasts can express glial fibrillary acidic protein (GFAP) in vivo. J. Neuropathol. Exp. Neurol. *60*, 449–461.
68. Danielyan, L., Tolstonog, G., Traub, P., Salvetter, J., Gleiter, C.H., Reisig, D., Gebhardt, R., and Buniatian, G.H. (2007). Colocalization of glial fibrillary acidic protein, metallothionein, and MHC II in human, rat, NOD/SCID, and nude mouse skin keratinocytes and fibroblasts. J. Invest. Dermatol. *127*, 555–563.
69. Xu, X., Yu, C., Xu, L., and Xu, J. (2022). Emerging roles of keratinocytes in nociceptive transduction and regulation. Front. Mol. Neurosci. *15*, 982202.
70. Moehring, F., Cowie, A.M., Menzel, A.D., Weyer, A.D., Grzybowski, M., Arzua, T., Geurts, A.M., Palygin, O., and Stucky, C.L. (2018). Keratinocytes mediate innocuous and noxious touch via ATP-P2X4 signaling. eLife *7*, e31684.
71. Sadler, K.E., Moehring, F., and Stucky, C.L. (2020). Keratinocytes contribute to normal cold and heat sensation. eLife *9*, e58625.
72. Ita, M.E., Singh, S., Troche, H.R., Welch, R.L., and Winkelstein, B.A. (2022). Intra-articular MMP-1 in the spinal facet joint induces sustained pain and neuronal dysregulation in the DRG and spinal cord, and alters ligament kinematics under tensile loading. Front. Bioeng. Biotechnol. *10*, 926675.
73. Allen, M., Ghosh, S., Ahern, G.P., Villapol, S., Maguire-Zeiss, K.A., and Conant, K. (2016). Protease induced plasticity: matrix metalloproteinase-1

CellPress
OPEN ACCESS

promotes neurostructural changes through activation of protease activated receptor 1. Sci. Rep. *6*, 35497.

74. Bastard, P., Zhang, Q., Cobat, A., Jouanguy, E., Zhang, S.Y., Abel, L., and Casanova, J.L. (2021). Insufficient type I IFN immunity underlies life-threatening COVID-19 pneumonia. C. R. Biol. *344*, 19–25.

75. Calabrese, L.H., Winthrop, K., Strand, V., Yazdany, J., and Walter, J.E. (2021). Type I interferon, anti-interferon antibodies, and COVID-19. Lancet Rheumatol. *3*, e246–e247.

76. Chauvineau-Grenier, A., Bastard, P., Servajean, A., Gervais, A., Rosain, J., Jouanguy, E., Cobat, A., Casanova, J.L., and Rossi, B. (2022). Autoantibodies Neutralizing Type I Interferons in 20% of COVID-19 Deaths in a French Hospital. J. Clin. Immunol. *42*, 459–470.

77. Hale, B.G. (2023). Autoantibodies targeting type I interferons: Prevalence, mechanisms of induction, and association with viral disease susceptibility. Eur. J. Immunol. *53*, e2250164.

78. Morgenroth, R., Reichardt, C., Steffen, J., Busse, S., Frank, R., Heidecke, H., and Mertens, P.R. (2020). Autoantibody Formation and Mapping of Immunogenic Epitopes against Cold-Shock-Protein YB-1 in Cancer Patients and Healthy Controls. Cancers (Basel) *12*, 3507.

79. Framil, M., García-Serrano, L., Morandeira, F., Luchoro, J.F., Antolí, A., Gomez-Vazquez, J.L., Sierra-Fortuny, À., and Solanich, X. (2025). Non-neutralizing anti-type I interferon autoantibodies could increase thrombotic risk in critical COVID-19 patients. Front. Immunol. *16*, 1556731.

80. Cooper, L., Xu, H., Polmear, J., Kealy, L., Szeto, C., Pang, E.S., Gupta, M., Kirn, A., Taylor, J.J., Jackson, K.J.L., et al. (2024). Type I interferons induce an epigenetically distinct memory B cell subset in chronic viral infection. Immunity *57*, 1037–1055.e6.

81. Chase, R., de la Peña, J.B., Smith, P.R., Lawson, J., Lou, T.F., Stanowick, A.D., Black, B.J., and Campbell, Z.T. (2022). Global analyses of mRNA expression in human sensory neurons reveal eIF5A as a conserved target for inflammatory pain. FASEB J. *36*, e22422.

82. Liu, H., Yan, Z., Zhu, D., Xu, H., Liu, F., Chen, T., Zhang, H., Zheng, Y., Liu, B., Zhang, L., et al. (2023). CD-NTase family member MB21D2 promotes cGAS-mediated antiviral and antitumor immunity. Cell Death Differ. *30*, 992–1004.

83. Do, L.D., Moritz, C.P., Muñiz-Castrillo, S., Pinto, A.L., Tholance, Y., Brugiere, S., Couté, Y., Stoevesandt, O., Taussig, M.J., Rogemond, V., et al. (2021). Argonaute Autoantibodies as Biomarkers in Autoimmune Neurologic Diseases. Neurol. Neuroimmunol. Neuroinflamm. *8*, e1032.

84. Zhang, H., Yue, J., Lian, C., Long, Y., and He, D. (2023). Case Report: Extraocular muscles paralysis associated with GAD65 antibody: a case series study. Front. Immunol. *14*, 1256089.

85. Fitzgerald, C.T., and Carter, L.P. (2011). Possible role for glutamic acid decarboxylase in fibromyalgia symptoms: A conceptual model for chronic pain. Med. Hypotheses *77*, 409–415.

86. Santos Guedes de Sa, K., Silva, J., Bayarri-Olmos, R., Brinda, R., Alec Rath Constable, R., Colom Diaz, P.A., Kwon, D.I., Rodrigues, G., Wenxue, L., Baker, C., et al. (2024). A causal link between autoantibodies and neurological symptoms in long COVID. Preprint at medRxiv. https://doi.org/10.1101/2024.06.18.24309100.

87. Mignolet M., Deroux C., Florkin T., Bielarz V., De Swert K., Halloin N., Sprimont L, Ladang A., George F., Gilloteaux J., et al. Pathogenic IgG from long COVID patients with neurological sequelae triggers sensitive but not cognitive impairments upon transfer into mice. bioRxiv. doi:10.1101/2025.11.20.689423.

88. Bevan, S., Javed, H., Israel, M., Primicheru, L., Mumu, M., Sun, H., Maurer, M., Oey, O., Fedele, L., Ribiero, A., et al. (2025). Autoantibodies Mediate Pain and Sensory Dysfunction in Post-COVID. Syndrome. https://doi.org/10.21203/rs.3.rs-7989936/v1.

89. Van Coillie, J., Pongracz, T., Rahmöller, J., Chen, H.J., Geyer, C.E., van Vught, L.A., Buhre, J.S., Šuštić, T., van Osch, T.L.J., Steenhuis, M., et al. (2023). The BNT162b2 mRNA SARS-CoV-2 vaccine induces transient afucosylated IgG1 in naive but not in antigen-experienced vaccinees. EBioMedicine *87*, 104408.

90. Krumholz, H.M., Wu, Y., Sawano, M., Shah, R., Zhou, T., Arun, A.S., Khosla, P., Kaleem, S., Vashist, A., Bhattacharjee, B., et al. (2023). Post-Vaccination Syndrome: A Descriptive Analysis of Reported Symptoms and Patient Experiences After Covid-19 Immunization. Preprint at medRxiv. https://doi.org/10.1101/2023.11.09.23298266.

91. Semmler, A., Mundorf, A.K., Kuechler, A.S., Schulze-Bosse, K., Heidecke, H., Schulze-Forster, K., Schott, M., Uhrberg, M., Weinhold, S., Lackner, K.J., et al. (2023). Chronic Fatigue and Dysautonomia following COVID-19 Vaccination Is Distinguished from Normal Vaccination Response by Altered Blood Markers. Vaccines *11*, 1642.

92. Sikkens, J.J., Buis, D.T.P., Peters, E.J.G., Dekker, M., Schinkel, M., Reijnders, T.D.Y., Schuurman, A.R., de Brabander, J., Lavell, A.H.A., Maas, J.J., et al. (2021). Serologic Surveillance and Phylogenetic Analysis of SARS-CoV-2 Infection Among Hospital Health Care Workers. JAMA Netw. Open *4*, e2118554.

93. Chaplan, S.R., Bach, F.W., Pogrel, J.W., Chung, J.M., and Yaksh, T.L. (1994). Quantitative assessment of tactile allodynia in the rat paw. J. Neurosci. Methods *53*, 55–63.

94. van Eenige, R., Verhave, P.S., Koemans, P.J., Tiebosch, I.A.C.W., Rensen, P.C.N., and Kooijman, S. (2020). RandoMice, a novel, user-friendly randomization tool in animal research. PLoS One *15*, e0237096.

STAR★METHODS

KEY RESOURCES TABLE

REAGENT or RESOURCE	SOURCE	IDENTIFIER
**Antibodies**		
Goat anti-Human IgG (H + L), Alexa Fluor 647 (secondary antibody)	Jackson ImmunoResearch Laboratories	109-606-088; RRID: AB_2337897
Human/Mouse GFAP Antibody Pair (capture & detection)	Abcam	ab244094
Mouse monoclonal anti-GFAP (clone GF12.24)	OriGene Technologies	BM2287
Donkey anti-Mouse IgG (H + L), Alexa Fluor 594 (secondary)	Invitrogen	A21203; RRID: AB_2535789
Rabbit polyclonal anti-Glutamine Synthetase (GS)	Abcam	ab73593; RRID: AB_2247588
Rat monoclonal anti-Mouse F4/80 (pan-macrophage, clone CI:A3-1)	Cedarlane Laboratories	CL8940AP; RRID: AB_10060355
Anti-Rabbit IgG (H + L), Alexa Fluor 750 (secondary)	Invitrogen	A21039; RRID: AB_10375716
Donkey anti-Rat IgG (H + L), Alexa Fluor 488 (secondary)	Invitrogen	A21208; RRID: AB_2535794
Rabbit monoclonal anti-ALDH1L1 (clone 7G8)	Invitrogen	14-9595-82; RRID: AB_2572952
Rabbit polyclonal anti-MAGEB10	Sino Biological	205166-T08
Rabbit monoclonal anti-AGO2	Sino Biological	50683-R036; RRID: AB_2860516
Rabbit monoclonal anti-KRT6A (clone YA1701)	MedChemExpress	HY-P81956
Rabbit monoclonal anti-GSTT1 (clone 2A7N6)	Invitrogen	MA5-55463; RRID: AB_3667703
Human monoclonal anti-GAD2 (GAD65) [HRP-conjugated]	Novus Biologicals	NBP3-28688H
Rabbit monoclonal anti-GIF (Intrinsic Factor)	Sino Biological	13544-R007
Rabbit polyclonal anti-MB21D2	Novus Biologicals	NBP1-79527
Goat anti-Human IgG Fc, PE-conjugated (secondary antibody)	SouthernBiotech	2040–09; RRID: AB_2795648
**Chemicals, peptides, and recombinant proteins**		
Protein G Sepharose 4 Fast Flow (for IgG purification)	Cytiva (GE Healthcare)	17061801
Slide-A-Lyzer MINI Dialysis Device (2 kDa MWCO)	Thermo Fisher Scientific	69576
NeuroTrace™ 435/455 Blue Fluorescent Nissl Stain	Thermo Fisher Scientific	N21479
Mouse GFAP protein, His-tag (standard)	Abcam	ab226309
Human ALDH1L1 protein	OriGene Technologies	TP313720
Human MAGEB10 protein	Novus Biologicals	NBP2-23225
Human AGO2 protein (Argonaute-2)	MedChemExpress	HY-P72835
Human KRT6A protein (Cytokeratin-6A)	Abnova	H00003853-P01
Human GSTT1 protein (Glutathione S-transferase theta-1)	Bio-Techne)	NBC1-28782
Human GAD2 protein (Glutamate decarboxylase 65)	ACROBiosystems	GA2-H5544
Human GIF protein (Gastric Intrinsic Factor)	Abcam	AB276557
Human MB21D2 protein (dCEF8)	OriGene Technologies	TP308468
**Critical commercial assays**		
HuProt™ Human Proteome Microarray	CDI Laboratories	HuProtV4.0_DECEMBER13_2021
MSD U-PLEX/R-PLEX Custom Multiplex Assay	Meso Scale Discovery	custom panel

(Continued on next page)

Continued

REAGENT or RESOURCE	SOURCE	IDENTIFIER
Olink Explore 3072 Proteomics Panel (plasma protein profiling)	Olink Proteomics	N/A
ProcartaPlex Mouse IL-4Rα Simplex Bead Kit	Thermo Fisher Scientific	EPX010-26102-901
ProcartaPlex Mouse Basic Kit (custom 5-plex cytokine panel)	Thermo Fisher Scientific	EPX010-20440-901
Deposited data		
HuProt autoantibody profile		Table S5

EXPERIMENTAL MODEL AND STUDY PARTICIPANT DETAILS

Study population

This study comprised Long COVID patients seen at the Amsterdam UMC outpatient Post COVID-19 Clinic. All patients were seen by a clinician, dedicated to post COVID-19, and had been diagnosed with Long COVID according to the WHO criteria (the continuation or development of new symptoms three months after SARS-CoV-2 infection, with these symptoms lasting for at least two months with no other explanation) and were required to have a reduction in working hours after SARS-CoV-2 infection. For the current study we selected patients between 18 and 65 years old who had previously a proven mild SARS-CoV-2 infection (non-hospitalized). Venous blood was obtained in the Amsterdam UMC post-COVID-19 biobank study, a minimum of 90 days after initial SARS-CoV-2 infection. The Amsterdam UMC post-COVID-19 Biobank study was approved by the institutional biobank ethics committee (Amsterdam UMC 2020_065). Demographics, comorbidities, symptomology and medications were derived from electronic health records. For sub-group analysis three Long COVID groups were created based on plasma biomarker analysis using Meso Scale Discovery (MSD). Patients were stratified in a two-step manner based on pre-specified markers. First, individuals with elevated GFAP and/or NFL relative to controls were assigned to LC-1. Patients with low GFAP but elevated NFL were also included in LC-1. The remaining patients were divided by IFN-β levels into LC-2 (high IFN-β) and LC-3 (low IFN-β). For this study we used three different control groups; healthy control subjects sampled prior to the SARS-CoV-2 pandemic (HC-pre) and healthy control subjects' samples after mild SARS-CoV-2 infection but without residual symptoms (HC-2022: S3 study, NL73478.029.20, $N = 15$[92] and HC-2024: MUSCLE-PASC/ME, $N = 15$). All blood samples were processed, aliquoted and frozen within 4h of blood draw. Written informed consent was obtained for all study participants.

Mouse model and behavioral assays

Experiments were conducted using adult male and female (aged 8–16 weeks) C57BL/6 mice (Janvier laboratories). Mice were maintained in the animal facility of the University of Utrecht and housed in groups under a 12h:12h light-dark cycle, with food and water available *ad libitum*. The cages contained environmental enrichment, including tissue papers and shelter. All experiments were performed in accordance with international guidelines and approved by the local experimental animal welfare body and the national Central Authority for Scientific Procedures on Animals (CCD, AVD11500202010805). All mice were acclimatized for the behavioral assays before measurements. Baseline measurements were performed before the mice were given any injection. After the baseline measurements, mice were injected intraperitoneal with ∼6.5 mg IgG/mouse (260mg/kg), approximately 1/3 of the total circulating mouse IgG.[37] The following behavioral tests were performed:

Heat withdrawal latency times were determined using the Hargreaves test (IITC Life Science).[38] Mechanical thresholds were determined using the von Frey test (Stoelting) with the up-and-down method previously described.[93] To minimize bias, animals were randomly assigned to the different groups prior to the start of experiment using Randomice software (v1.1.5, GitHub) based on the following variables: age, weight, cage mechanical sensitivity (Von Frey test) and locomotor activity (rotarod and open field tests) at baseline.[94] All experiments were performed by operators blinded to the treatments.

Local motor activity and stamina was determined with an open field test and rotarod analysis, respectively. Mice from different groups were tested interspersed throughout the trials. To measure local motor activity, one mouse at a time was placed in the center of an arena (30 cm × 15 cm) and spontaneous behavior was recorded for 30 min (video camera imagingsource DMK22AUC03) and analyzed (i.e., distance, immobility) using the ANY-maze software. To assess neurological deficits, like motor performance and stamina, mice were placed in the rotarod at a fix rotation of with accelerated speed.[40] Time to fall was recorded for 300s with 1) a fixed rotation of 12 rpm or 2) during the acceleration test which started at 4 rpm and increased overtime to 40 rpm. The open field arena and rotarod were cleaned thoroughly with a 5% alcohol/water solution between each mouse to minimize odor cues.

METHOD DETAILS

Meso Scale Discovery (MSD) multiplex assay

U-PLEX and R-PLEX Custom Human Cytokine assays were employed for the detection of IL-1β, IL-6, IL-10, TNF, IFN-α2a, IFN-β, IFN-γ, GFAP, neurofilament L (NFL), and total Tau. The analysis was performed on EDTA plasma (EDTA tube, 2000g for 10 min) of 34 Long COVID and 22 post-COVID non-Long COVID healthy controls (HC-2022, $N = 15$; HC-2024, $N = 7$). The lyophilized single or cocktail mix calibrators were reconstituted in provided assay diluents. MSD plates were prepared by coating them with supplied linkers and biotinylated capture antibodies as per the manufacturer's instructions. The assays were conducted according to the manufacturer's protocol, with the undiluted plasma samples and standards incubated overnight at 4°C. Electrochemiluminescence signals were measured using a MESO QuickPlex SQ 120 plate reader (MSD) and analyzed using Discovery Workbench Software (v4.0, MSD). The concentration of each sample was determined using a four-parameter logistic model generated with the standards, and the concentrations were calculated based on the certificate of analysis provided by MSD. Concentrations below the lower limit of detection were imputed as half of the lowest detected value.

OLINK proteomics

Olink Proteomics technology was employed for protein profiling analysis. Due to limited samples available, the analysis was performed on EDTA plasma (EDTA tube, 2000g for 10 min) of 31 out of 34 Long COVID patients. Per protocol, samples were randomized across plates and run alongside a negative control (buffer), plate control, and a sample control. A total of 2,944 proteins were measured, with 2,865 proteins quantified after quality check filtering. For data normalization we employed the normalizeVSN and normalizeQuantiles packages. Normalized protein expressions (NPX) are reported as log2 values as per OLINK protocol.

Human IgG purification

IgG was purified from 800 μL of patient or healthy donor sera/EDTA plasma using Protein G-conjugated beads (Cytiva cat# 17061801). Protein G beads were added to 1-mL gravity flow columns (Thermo Scientific, 89896) and washed with PBS. Serum/plasma was diluted 1:1 with PBS (pH = 7) and applied to the washed column. The flow-through was collected and reapplied to the column five times to increase recovery. After washing, bound IgG was eluted with 0.1 M glycine (pH 2.7) and immediately neutralized with 1 M Tris buffer (pH 9). Protein concentration was determined by Nanodrop spectrophotometry. Residual salt was removed using Slide-A-Lyzer MINI Dialysis Device (Thermo Scientific). The IgG were then concentrated in saline buffer using Vivaspin to achieve a final concentration of 13 mg/mL. Purity of the concentrated IgG was verified by SDS-PAGE and Coomassie Blue staining, which showed clean IgG bands without detectable contaminants. Endotoxin levels in the final IgG preparations were confirmed to be undetectable using the Pierce Chromogenic Endotoxin Quant Kit (Thermo Scientific, A39552).

Immunohistochemistry

Murine lumbar spinal cord and lumbar dorsal (L3-L5) root ganglia (DRG) tissue were embedded and frozen in optimal cutting temperature (OCT) freezing matrix (Sakura) using dry ice. Tissue slides of 10 μm thick were cut (Leica CM3050 S Cryostat) and kept at −80°C. On the day of staining, tissue slides were thawed at room temperature for 30 min and tissue was encircled using a Dako-pen. Slides were washed 3-times with 200 μL PBS, and fixated with 4% paraformaldehyde for 10 min. After washing slides with PBS with 0.3% Triton X-100 twice for 5 min. Tissue was blocked with 2% IgG-free BSA blocking buffer (ImmunoResearch cat:001-000-161). After tapping the blocking buffer from slides, they were incubated for 2 h with 200 μL 1:250 goat anti-human IgG-Alexa fluor-647 in 3-time diluted blocking buffer at room temperature (ImmunoResearch Jackson cat:109-606-088). Subsequently, tissue slides were washed 3-times with diluted blocking buffer and further blocked with 200 μL 1:500 human IgG (nanogram, Sanquin) for 30 min. Slides were washed 1-time with diluted blocking buffer. Spinal cord sections were incubated with, mouse anti-GFAP (OriGene, BM2287; 1:200) overnight at 4°. The next day, slides were washed with 1:3 diluted blocking buffer 3-times for 1 min. Subsequently, slides were incubated with anti-mouse Alexa Fluor 594 antibody (Invitrogen, A21203; 1:500) for 90 min at room temperature. DRG sections were incubated with rabbit anti-glutamine synthesis (Abcam, ab73593; 1:200), mouse anti-GFAP (OriGene, BM2287; 1:200) and rat anti-F4/80 (Cedarlane, CL8940AP; 1:500) overnight at 4°C. The next day, slides were washed with 1:3 diluted blocking buffer (3-times, 1 min) and incubated with anti-mouse Alexa Fluor 594 antibody (Invitrogen, A21203), anti-rabbit Alexa Fluor 750 (Invitrogen, A21039), and anti-rat Alexa Fluor 488 (Invitrogen, A21208) all at 1:500 for 90 min at room temperature. After washing 3-times with PBS, slides were incubated with NeuroTrace 435/455 Blue Fluorescent Nissl Stain (Thermo Fisher, N21479; 1:300) for both spinal cord and DRG for 20 min. After washing sections were imbedded using FluoSafe (Merck, 345789) and kept at 4°C until imaging. Imaging was performed using a Thunder Wide Field Fluorescence microscope (Leica). The intensity and area of human IgG staining in mice was quantified using ImageJ software. The positive area for IgG was defined as the area with set pixel intensity and normalized to the total area of the section.

Autoantibody analysis by HuProt

Pooled isolated IgG samples were analyzed using HuProt human proteome arrays (CDI Laboratories) per manufacturer's protocol. In short, the arrays were first blocked (0.1% Tween 20, 5% BSA, TBS) at room temperature for 1 h. Subsequently, the antibodies were applied to HuProt arrays at a concentration of 1 μg/mL in the blocking buffer and incubated at room temperature for 1 h. After probing,

the arrays were washed three times with TBST (1xTBS, 0.1% Tween 20) for 10 min each. Human antibodies were detected with Alexa 647-*anti*-human IgG Fc secondary antibody (0.25 μg/mL) at room temperature for 1 h, followed by 3 washes with TBST and 3 rinses with ddH2O. The arrays were then dried and scanned using a GenePix 4000B scanner for data collection. Local background fluorescence was first subtracted from each protein spot on the HuProt arrays to remove ambient signal. IgG spot intensities were then normalized across arrays and blocks using a robust linear model[42] and standardized within each block to the on-array human IgG calibration spots. Long COVID-associated autoreactivities were identified as protein features with signal intensities >2-fold higher than those of pooled pre-pandemic healthy controls and exceeding the intensity of the 1 μg/mL on-array IgG standard.

Mouse plasma biomarker quantification by Luminex

Mouse plasma collected 15 days post human IgG injection was analyzed using Luminex-based bead assays (ProcartaPlex, Life Technologies Austria). The Mouse IL-4R Simplex Kit (EPX010-26102-901) and Mouse Basic Kit (EPX010-20440-901) customized with IL12-p70, CXCL10, IFN-α2a, IFN-β, IFN-γ analytes were used according to the manufacturer's protocol. For GFAP, custom assay was generated using paired capture/detection antibodies (ab244094, Abcam) with recombinant mouse GFAP-His (ab226309, Abcam) as the standard. Samples and standards were measured and concentrations were calculated from 4-parameter logistic standard curves.

Luminex-based autoantibody validation

Recombinant human proteins, ALDH1L1 (OriGene, TP313720), MAGEB10 (Novus Biologicals, NBP2-23225), AGO2 (MedChemExpress, HY-P72835), KRT6A (Abnova, H00003853-P01), GSTT1 (Novus Biologicals, NBC1-28782), GAD2 (ACROBiosystems, GA2-H5544), GIF (Abcam, AB276557), MB21D2 (OriGene, TP308468), and BSA (Sigma-Aldrich, A1595) were covalently coupled to carboxylated MagPlex microspheres (Luminex) per manufacturer's protocol. Coupling efficiency and protein integrity were confirmed using antigen-specific monoclonal antibodies: anti-ALDH1L1 (Invitrogen, 14-9595-82), anti-MAGEB10 (Sino Biological, 205166-T08), anti-AGO2 (Sino Biological, 50683-R036), anti-KRT6A (MedChemExpress, HY-P81956), anti-GSTT1 (Invitrogen, MA5-55463), anti-GAD2 (Bio-Techne, NBP3-28688H), anti-GIF (Sino Biological, 13544-R007), anti-MB21D2 (Novus Biologicals, NBP1-79527). Pooled IgG from Long COVID patients, pre-pandemic healthy controls, or post-COVID healthy controls was diluted in assay buffer (PBS, 0.1% BSA, 0.02% Tween 20, 0.05% sodium azide) and incubated with antigen-coated beads overnight at 4°C while shaking at 600 rpm. After washing, bound human IgG was detected with phycoerythrin (PE)-conjugated goat anti-human IgG Fc (Southern Biotech, 2040-09) at 2 μg/mL for 30 min at room temperature shaking at 600 rpm. Fluorescence intensity was measured and median fluorescence intensity (MFI) values were recorded per bead set. Each assay included monoclonal or polyclonal antibody standards to ensure measurements were within a linear range.

QUANTIFICATION AND STATISTICAL ANALYSIS

For subject demographics and targeted biomarker analysis, histograms and Shapiro-Wilk tests were employed to assess data distributions and normality. Categorical values were depicted in absolute numbers alongside percentages in brackets. Parametric quantitative variables were shown as means ± standard deviation, while nonparametric quantitative variables were presented as median and interquartile ranges (25th and 75th percentiles). For case-control and subgroup comparisons of targeted plasma biomarkers, we fit linear regression models on transformed concentrations with group (LC vs. control) or subgroup (LC-1/LC-2/LC-3) as the main effect and age, sex, and days-since-most-recent infection as covariates (model: $y = \beta 0 + \beta 1_{group} + \beta 2_{age} + \beta 3_{sex} + \beta 4_{days\_since\_infection} + \varepsilon$) Estimated marginal means and pairwise contrasts were obtained with emmeans, p values were from F-tests on the group/subgroup term with Satterthwaite df, and Benjamini-Hochberg (BH) correction was applied across markers and pairwise tests. Principal component analysis of the MSD data was performed with raw intensity instead of standard-based converted concentrations to avoid missing values. Categorical data were analyzed using Fisher's exact test. Non-normally distributed data underwent Box-Cox transformation. Continuous parametric data were assessed using either a t test analysis of variance, with Tukey HSD post-hoc testing applied when appropriate. Continuous nonparametric data were analyzed using the Mann-Whitney U test, Kruskal-Wallis H test, or pairwise Kruskal-Wallis test with Benjamini-Hochberg (BH) correction where appropriate. For Olink proteomics, partial least squares discriminant analysis (PLS-DA) and differential analysis for variance-stabilized quantile-normalized data were conducted using R packages mixOmics (v.6.24.0) and limma (v3.56.2). Principle components derived from PLS-DA were subjected to Gene Set Enrichment Analysis using R package fgsea (v.1.26.0). For tissue enrichment analysis, we contrasted tissue-selective gene sets from GTEx (v8; median nTPM). We used the moderated t statistic (limma) to rank features for GSEA (fgsea). Pathway analyses of the autoantigens were performed using the Metascape platform on 2025-09-02. In the behavioral assessment of mice, repeated measured data was adjusted for pre-human IgG injection baseline measurements. Post-hoc comparisons of continuous data were performed using Empirical Mean Differences with BH adjustment with R package emmeans (v.1.10.1). A significance threshold of $p < 0.05$ was applied. The analyses and visualization were executed under R environment (v.4.5.0) or using GraphPad Prism (v.9.0).

Cell Reports Medicine, Volume *7*

Supplemental information

Transfer of IgG from long COVID patients induces symptomology in mice

Hung-Jen Chen, Brent Appelman, Hanneke L.D.M. Willemen, Amelie Bos, Judith Prado, W. Ashwin Mak, Noa Keijzer, Patrícia Silva Santos Ribeiro, Sara Vieira Goncalves, Sabine Versteeg, Chiara.E. Geyer, Mads Larsen, Eline Schüchner, Marije K. Bomers, Ayesha H.A. Lavell, Amsterdam UMC COVID-19 biobank, Braeden Charlton, Rob Wüst, W. Joost Wiersinga, Michèle van Vugt, Gestur Vidarsson, Niels Eijkelkamp, and Jeroen den Dunnen

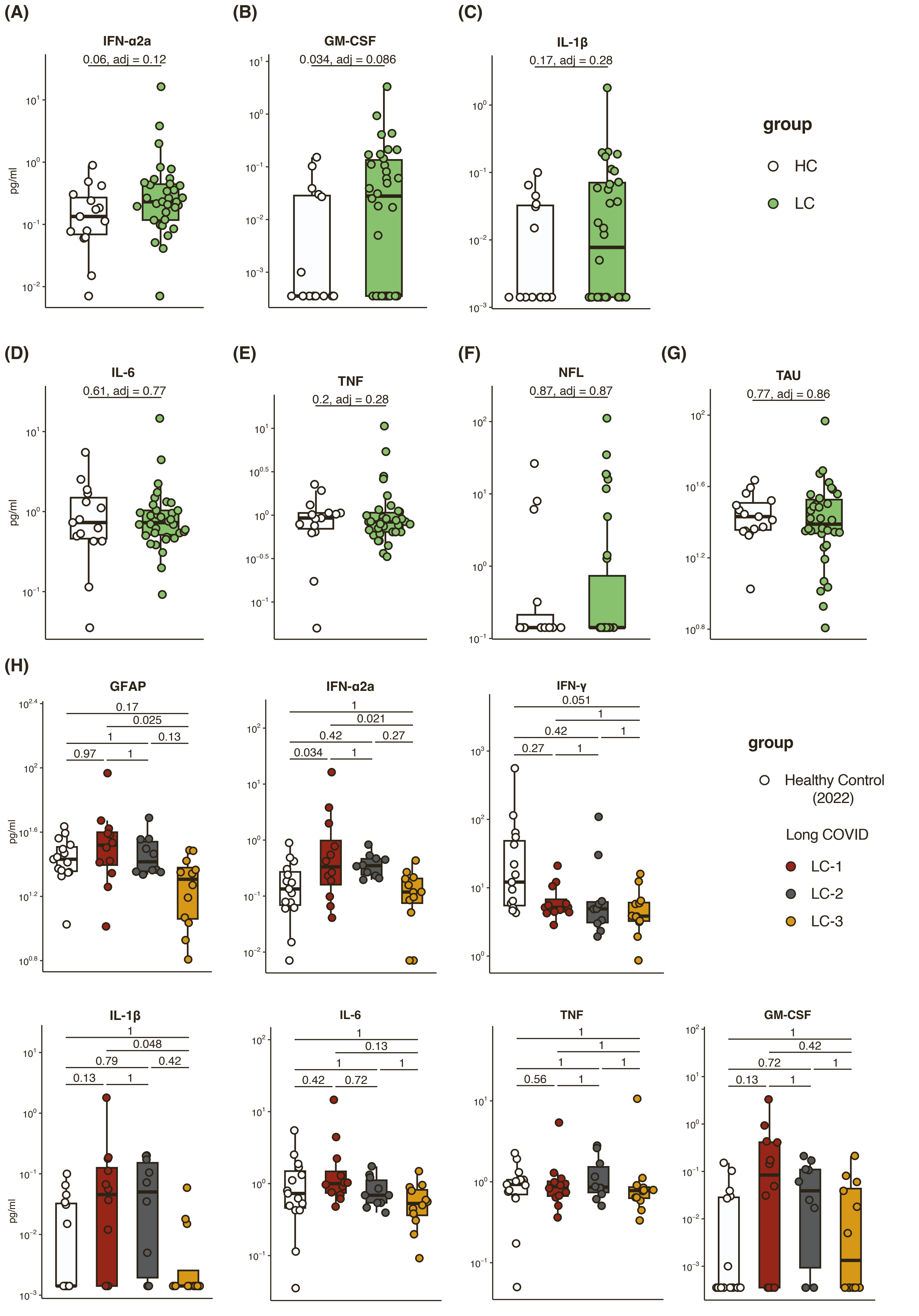
(A)
IFN-α2a
0.06, adj = 0.12
pg/ml
(B)
GM-CSF
0.034, adj = 0.086
(C)
IL-1β
0.17, adj = 0.28
group
HC
LC
(D)
IL-6
0.61, adj = 0.77
(E)
TNF
0.2, adj = 0.28
(F)
NFL
0.87, adj = 0.87
(G)
TAU
0.77, adj = 0.86
(H)
GFAP
IFN-α2a
IFN-γ
IL-1β
IL-6
TNF
GM-CSF
group
Healthy Control (2022)
Long COVID
LC-1
LC-2
LC-3

Figure S1: Plasma biomarkers of Long COVID compared to healthy SARS-CoV-2 recovered participants. Targeted quantitative measurements of cytokines and neuro-injury markers of (A-G) 34 Long COVID patients (LC) and 15 healthy controls (HC) with prior SARS-CoV-2 infection using MSD. (H) Subgroup comparisons. Boxes show median and IQR; points are individuals. Numbers above brackets are p values with BH-adjusted p values in parentheses from linear models with group/endotype as the main effect and age, sex, and days-since-infection as covariates; pairwise contrasts are BH-corrected unless stated. All values are shown in pg/ml on a logarithmic y-axis Shown is the interquartile range and the median. ns, not significant. Abbreviations: HC, healthy control; LC, Long COVID; NFL, neurofilament light chain.

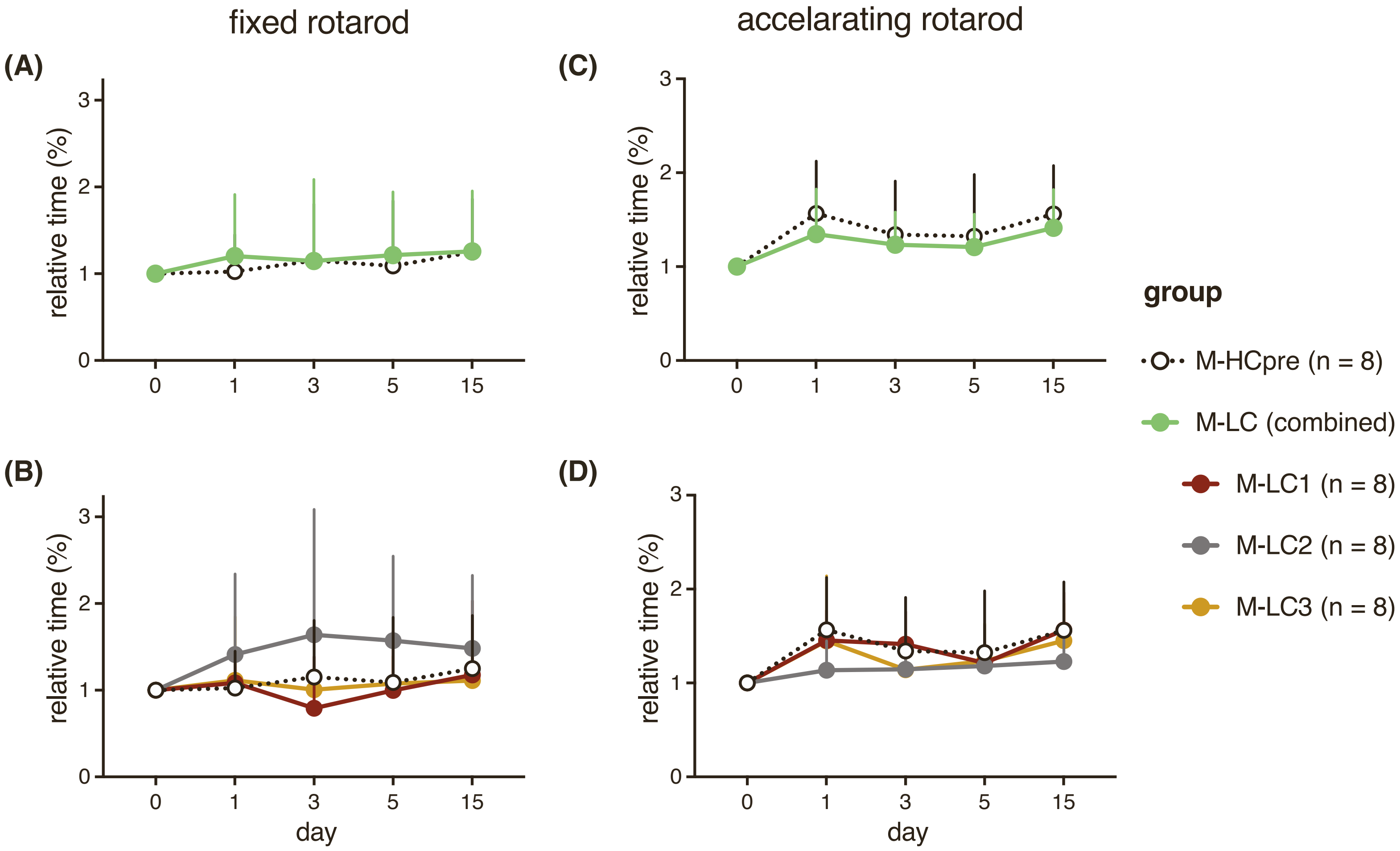

Figure S2. IgG from Long COVID patients did not change time spent on the rotarod. Rotarod test of mice injected with IgG for pre-pandemic healthy controls (M-HCpre) of Long COVID patients (M-LC) (A-B) relative time spent on the rotarod in the fixed-speed test (C-D) relative time spent on the rotarod in the accelerating test. Data points are shown as the mean + SD. Statistical significance is calculated with a linear mixed effects model with post-hoc comparison using the emmeans package with BH adjustment. Shown is the mean with standard deviation.

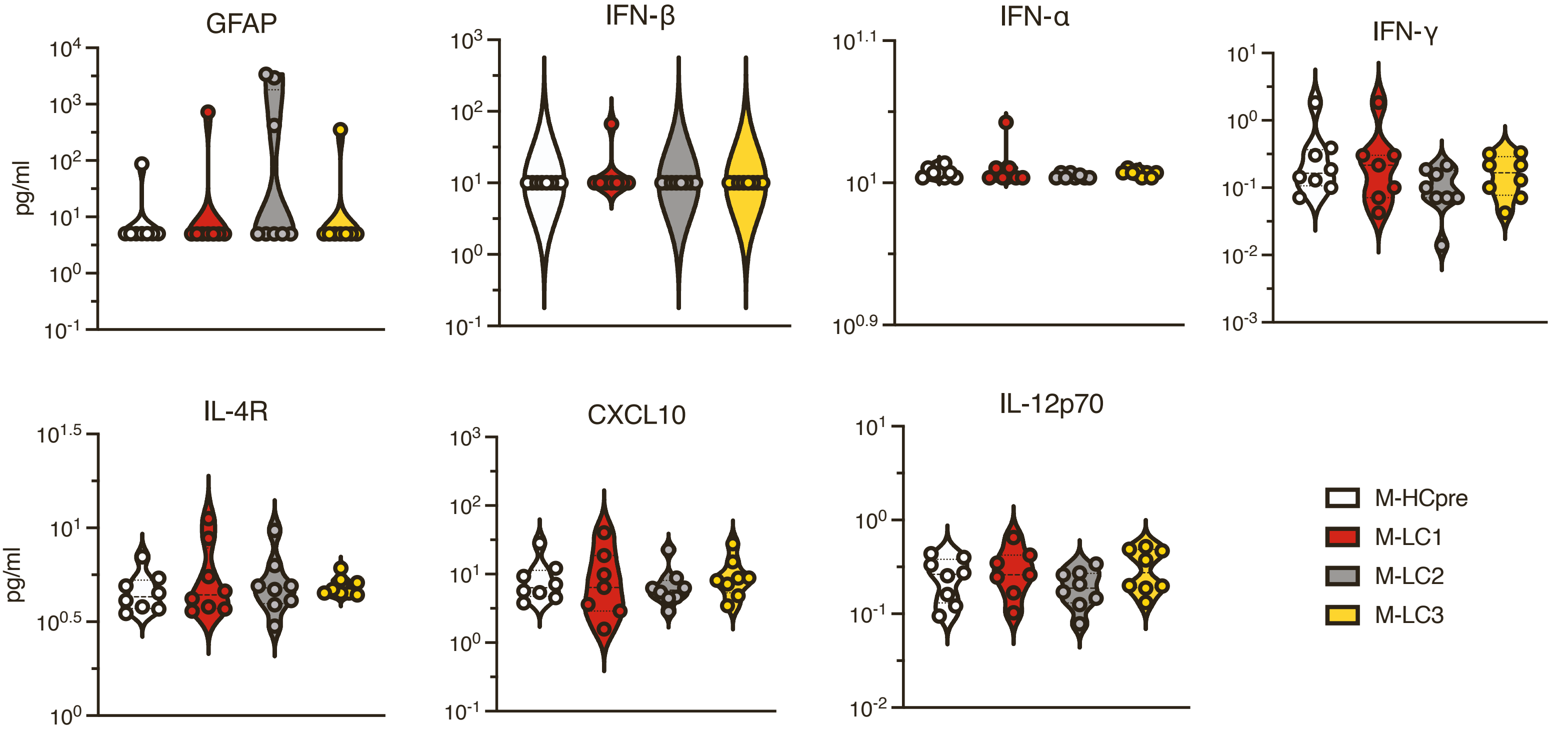

Figure S3. Plasma cytokine levels in recipient mice measured by Luminex at day 15 after injection with pooled human IgG from pre-pandemic healthy controls (M-HCpre) or Long COVID subgroups (M-LC1, M-LC2, M-LC3). Undetectable samples were imputed as half of the detection limit.

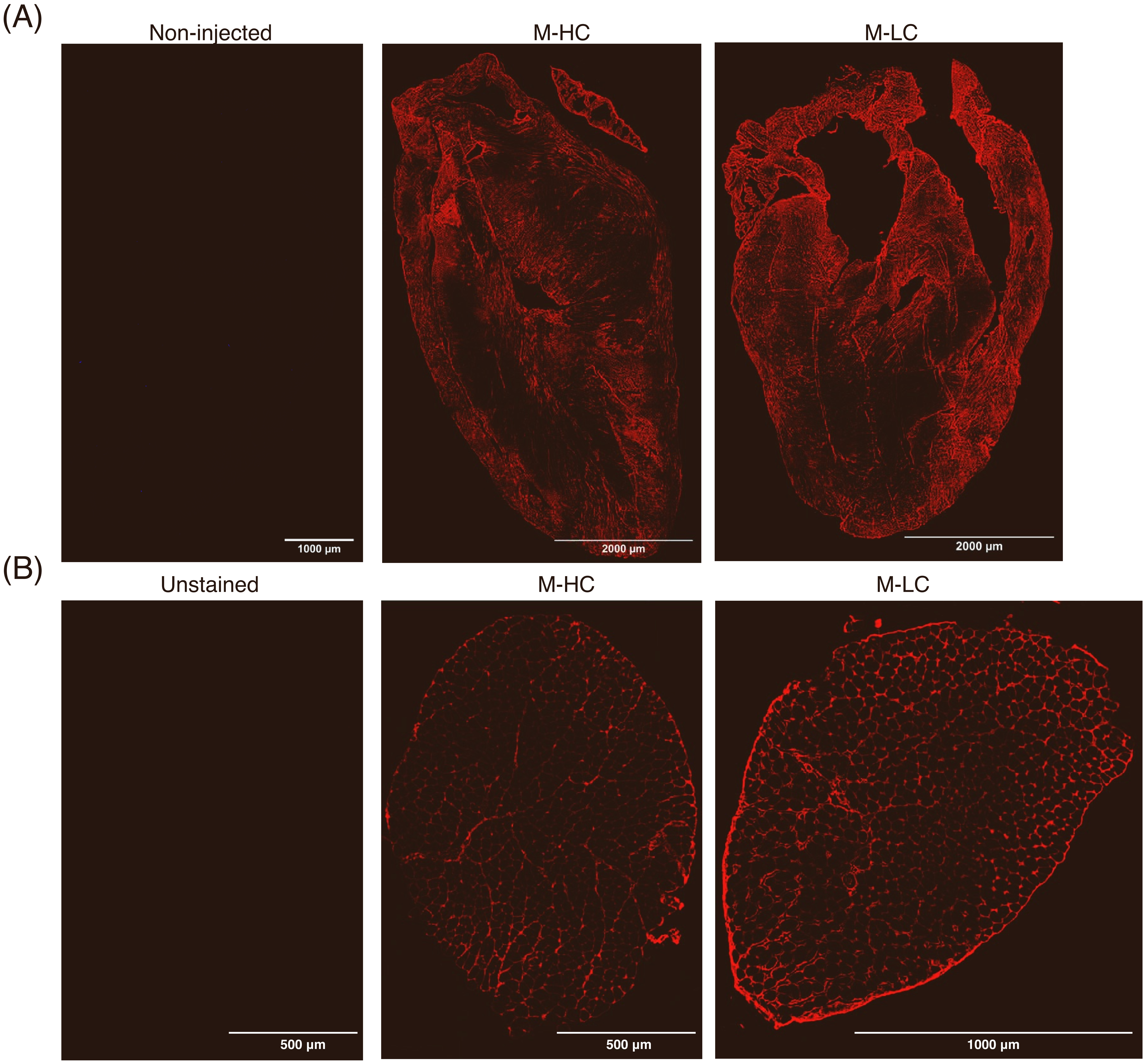
(A)
Non-injected
M-HC
M-LC
1000 µm
2000 µm
2000 µm
(B)
Unstained
M-HC
M-LC
500 µm
500 µm
1000 µm

Figure S4: Detection of injected human IgG (hIgG) in murine heart and skeletal muscle 15-day post-injection. (A) Representative staining of hIgG (red) in hearts from non-injected mice (left), mice injected with pooled IgG from pre-pandemic healthy controls (M-HCpre, middle), or Long COVID patients (M-LC, right. Shown example from the M-LC2 subgroup). (B) Representative staining of hIgG in skeletal muscle from unstained M-LC (left, shown example from the M-LC2 subgroup), M-HCpre (middle), and M-LC (right, shown example from the M-LC2 subgroup). Scale bars, 500-2000 μm.

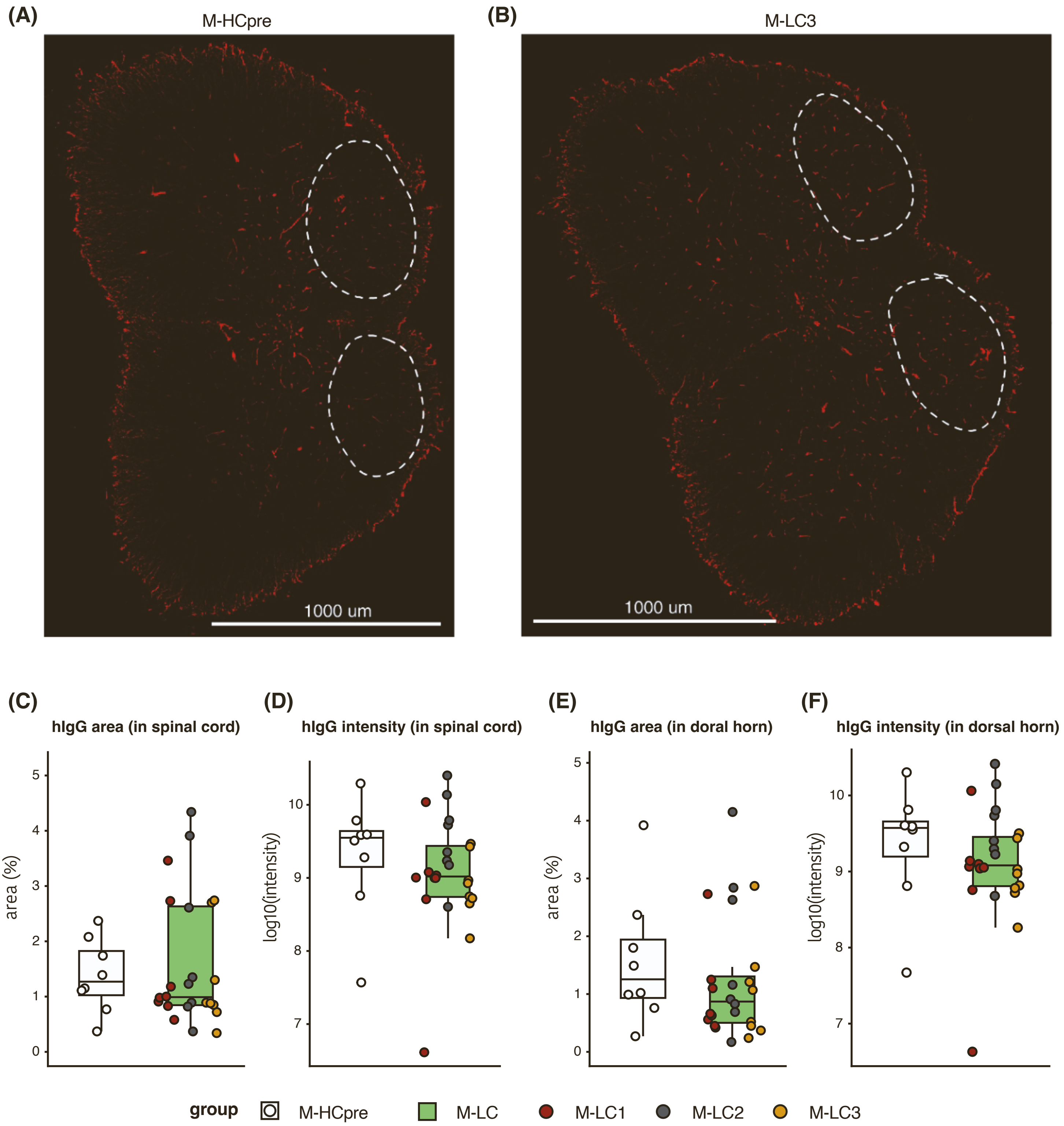

Figure S5: Detection of injected human IgG (hIgG) in murine spinal cord 15-day post-injection. (A-B) Representative staining of hIgG (red) in spinal cord sections from mice injected with pooled IgG from pre-pandemic healthy controls (M-HCpre, A) or Long COVID patients (M-LC, B; Shown example from the M-LC3 subgroup). White dashed circles indicate the dorsal horn area. Scale bars, 1000 μm. (C) Quantification of hIgG-positive area normalized to total spinal cord area per mouse. (D) Quantification of hIgG intensity in the spinal cord. (E) Quantification of hIgG-positive area within the dorsal horn region. Boxes show median and IQR. (F) Quantification of hIgG intensity within the dorsal horn region. Each dot represents one mouse; colors indicate IgG source: M-HCpre (white), M-LC1 (red), M-LC2 (grey), M-LC3 (yellow). No significant differences were observed across (sub)groups by two-sided unpaired t-tests.

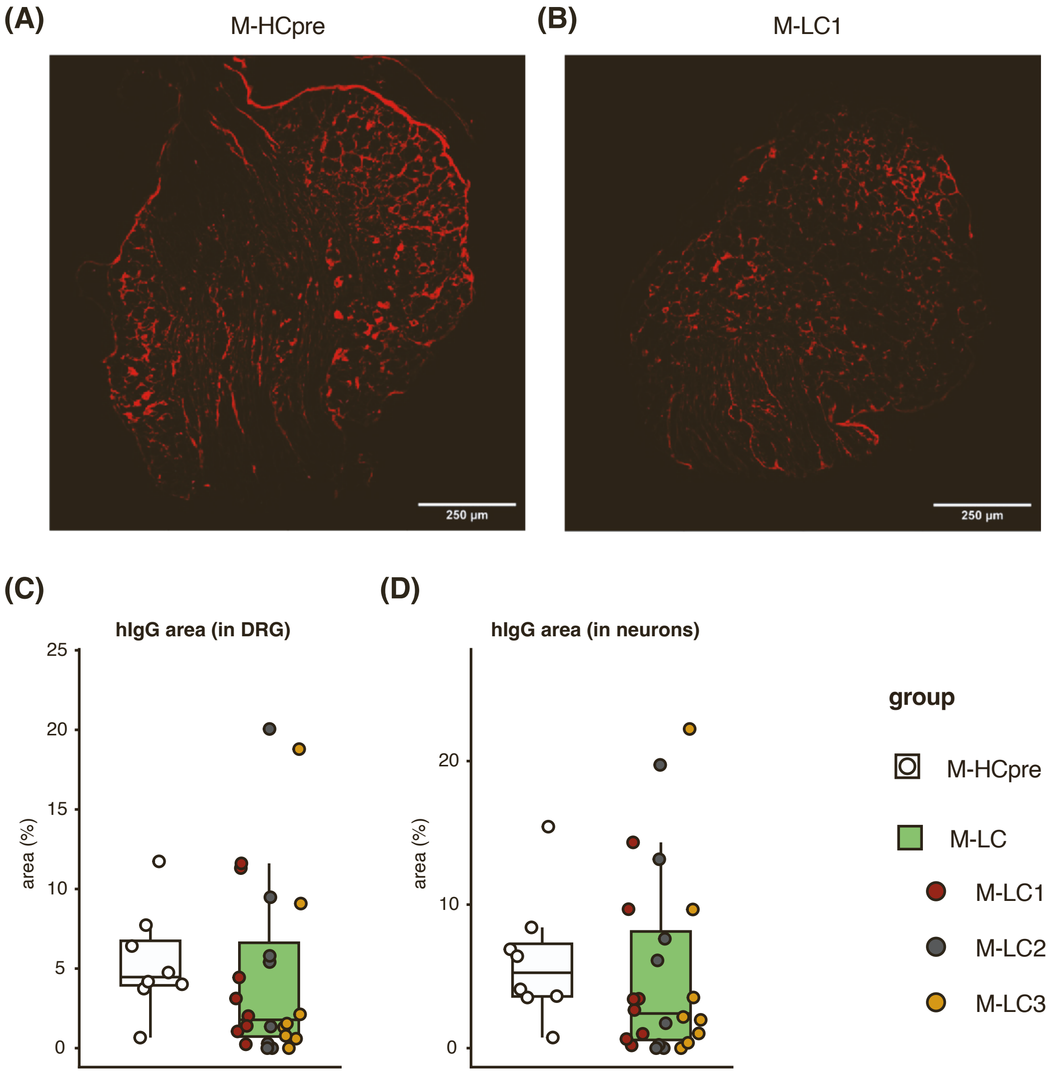

Figure S6: Detection of injected human IgG (hIgG) in murine dorsal root ganglia (DRG) 15-day post-injection. (A-B) Representative staining of hIgG (red) in DRG from mice injected with pooled IgG antibodies from pre-pandemic healthy controls (M-HCpre, A) or Long COVID patients (M-LC, B; Shown example from the M-LC1 subgroup). Scale bars, 250 µm. (C) Quantification of hIgG-positive area normalized to total DRG area per mouse. (D) Quantification of hIgG-positive area within neuronal regions (soma-containing area). Boxes show median and IQR. Each dot represents one mouse; colors indicate the IgG source: M-HCpre (white), M-LC1 (red), M-LC2 (grey), M-LC3 (yellow). No significant differences were observed across (sub)groups by two-tailed unpaired t-tests.

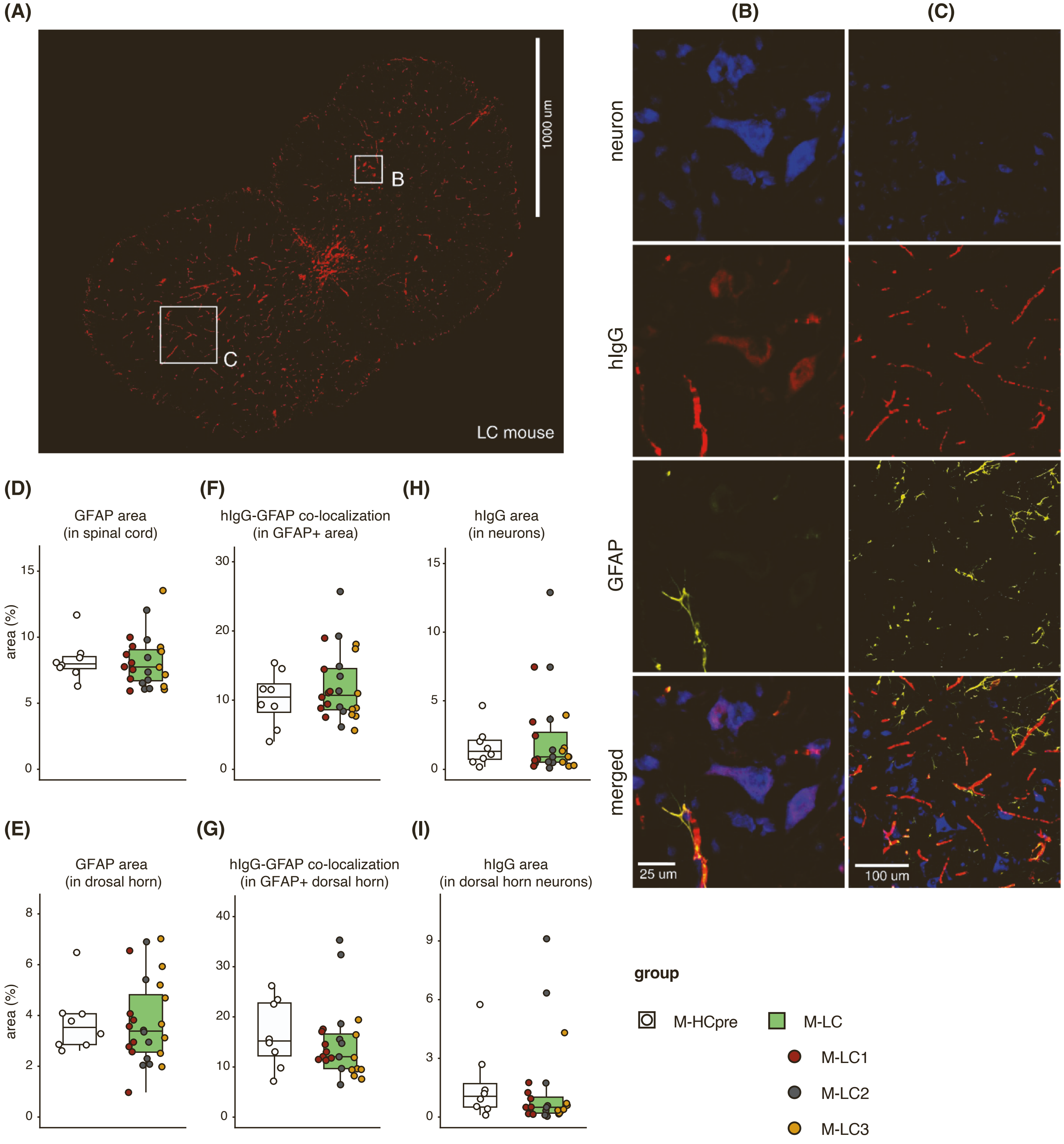

Figure S7: Co-localization of injected human IgG (hIgG) with neurons and astrocytes in murine spinal cord 15-day post-injection. (A) Representative staining of spinal cord sections from mice injected with pooled IgG from Long COVID patients (M-LC, Shown example from the M-LC1 subgroup). Sections were stained for neurons (NeuroTrace, blue), hIgG (red), and astrocytes (GFAP, yellow). White boxes indicate regions magnified in panels (B-C). (B) Magnified view showing hIgG co-localization with neurons. (C) Magnified view showing partial co-localization of hIgG with astrocytes. (D-E) Quantification of GFAP-positive area in total spinal cord (D) and dorsal horn (E). (F-G) Quantification of hIgG co-localization with GFAP in total spinal cord (F) and dorsal horn (G). (H-I) Quantification of hIgG co-localization with NeuroTrace in total spinal cord (H) and dorsal horn (I). Boxes show median and IQR. Each dot represents one mouse; colors indicate IgG source: M-HCpre (white), M-LC1 (red), M-LC2 (grey), M-LC3 (yellow). No significant differences were observed across (sub)groups by two-tailed unpaired t-tests. Scale bars, 1000 μm (A), 25-100 μm (B-C).

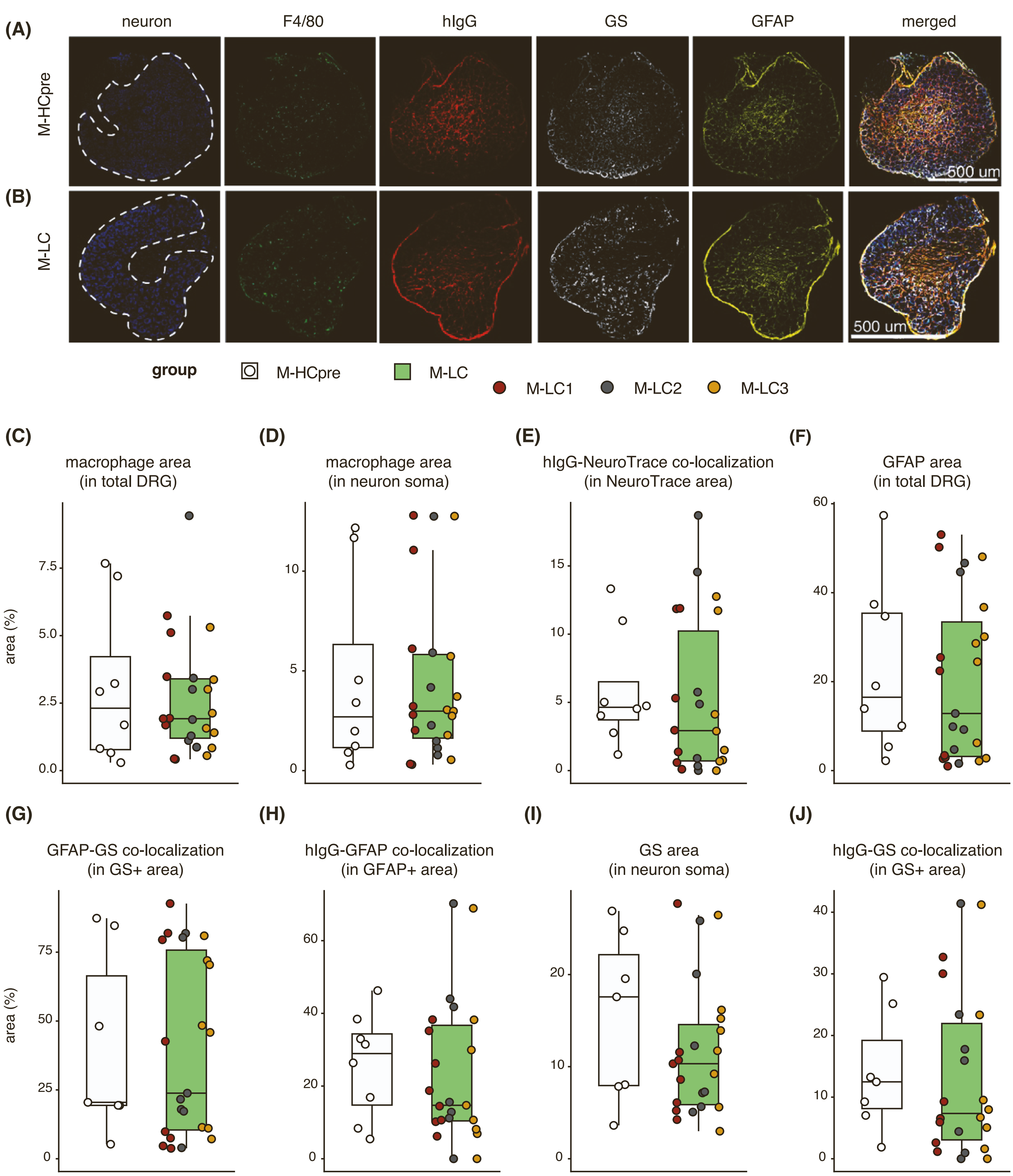
(A)
neuron
F4/80
hIgG
GS
GFAP
merged
M-HCpre
500 um
(B)
M-LC
500 um
group
M-HCpre
M-LC
M-LC1
M-LC2
M-LC3
(C)
macrophage area (in total DRG)
area (%)
(D)
macrophage area (in neuron soma)
(E)
hIgG-NeuroTrace co-localization (in NeuroTrace area)
(F)
GFAP area (in total DRG)
(G)
GFAP-GS co-localization (in GS+ area)
area (%)
(H)
hIgG-GFAP co-localization (in GFAP+ area)
(I)
GS area (in neuron soma)
(J)
hIgG-GS co-localization (in GS+ area)

Figure S8: Detection of injected human IgG (hIgG) and immune cell and neuron activation markers in murine dorsal root ganglia (DRG) 15-day post-injection. (A-B) Representative staining of DRG from mice injected with pooled IgG from pre-pandemic healthy controls (M-HCpre, A) or Long COVID patients (M-LC, B; shown example from the M-LC3 subgroup). Sections were stained for neurons (NeuroTrace, blue), macrophages (F4/80, green), hIgG (red), satellite glial cells (glutamine synthetase, GS, white), and astrocytes (GFAP, yellow). White dashed outlines indicate the soma-containing region of the DRG defined by NeuroTrace labeling. Scale bars, 500 µm. (C-D) Quantification of F4/80^{+} macrophage area in total DRG (C) and within neuronal soma regions (D). (E) Quantification of hIgG co-localization with NeuroTrace staining. (FG) Quantification of GFAP^{+} and GS^{+} areas in DRG soma-containing regions. (H-J) Quantification of hIgG co-localization with GFAP^{+} (H) and GS^{+} (J) areas, and total GS^{+} area in neuronal soma (I). Boxes show median and IQR. Each dot represents one mouse; colors indicate IgG source: M-HCpre (white), M-LC1 (red), M-LC2 (grey), M-LC3 (yellow). No significant differences were observed across groups by two-sided t-tests.

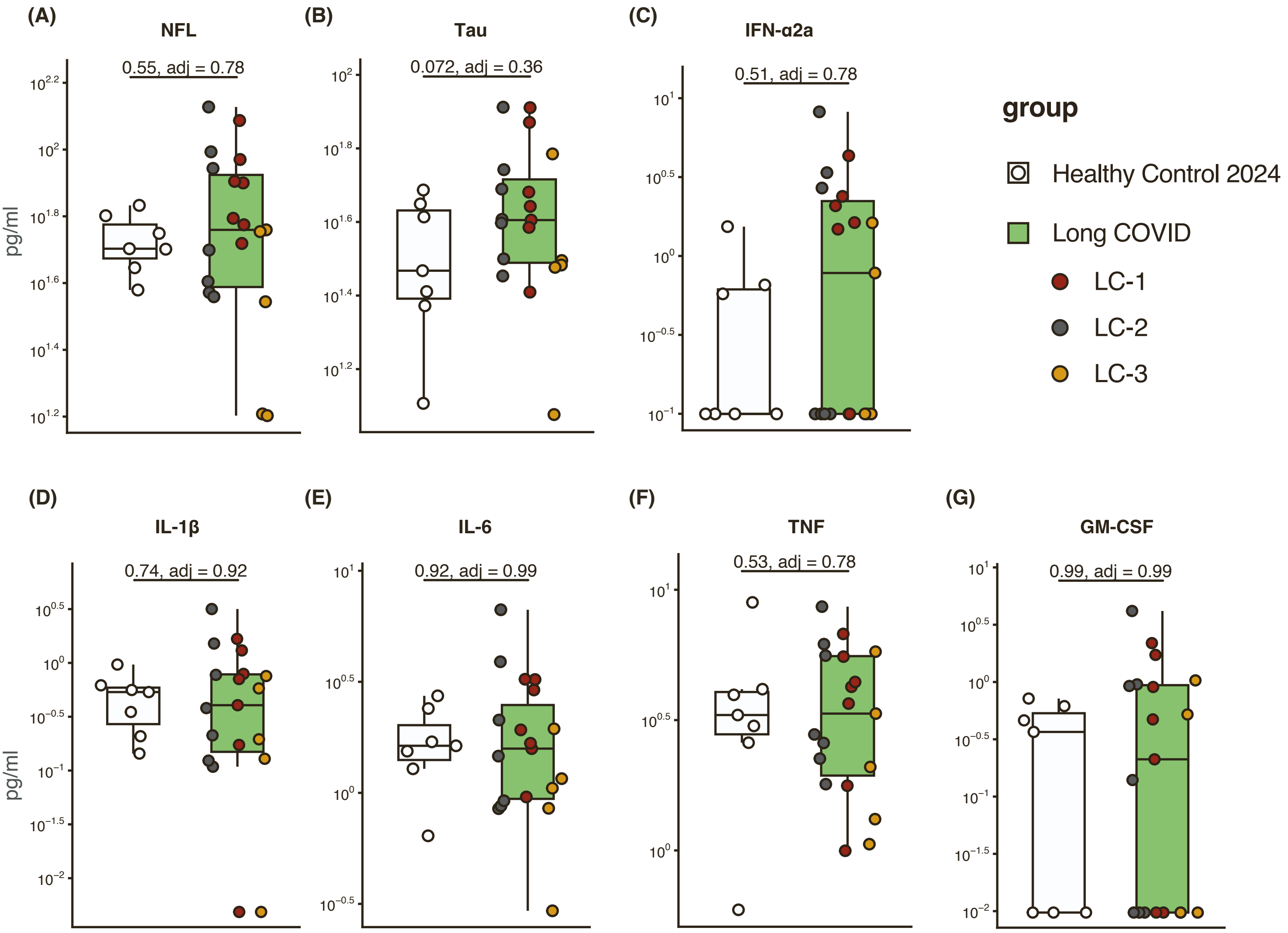

Figure S9. Two-year follow-up plasma biomarkers of Long COVID and post-COVID healthy cohort (2024) and subgroup comparisons. Targeted quantitative measurements using MSD of plasma biomarkers in follow-up plasma from (A-G) the same LC patient group (green) versus a new cohort of post-COVID, non-LC healthy controls (HC2024; white). Boxes show median and IQR; points are individuals. Individual LC donors are colored by subgroup: LC-1 (red), LC-2 (grey), LC-3 (yellow). Numbers above brackets are p values with BH-adjusted p values in parentheses from linear models adjusted for age, sex, and days-since-infection.

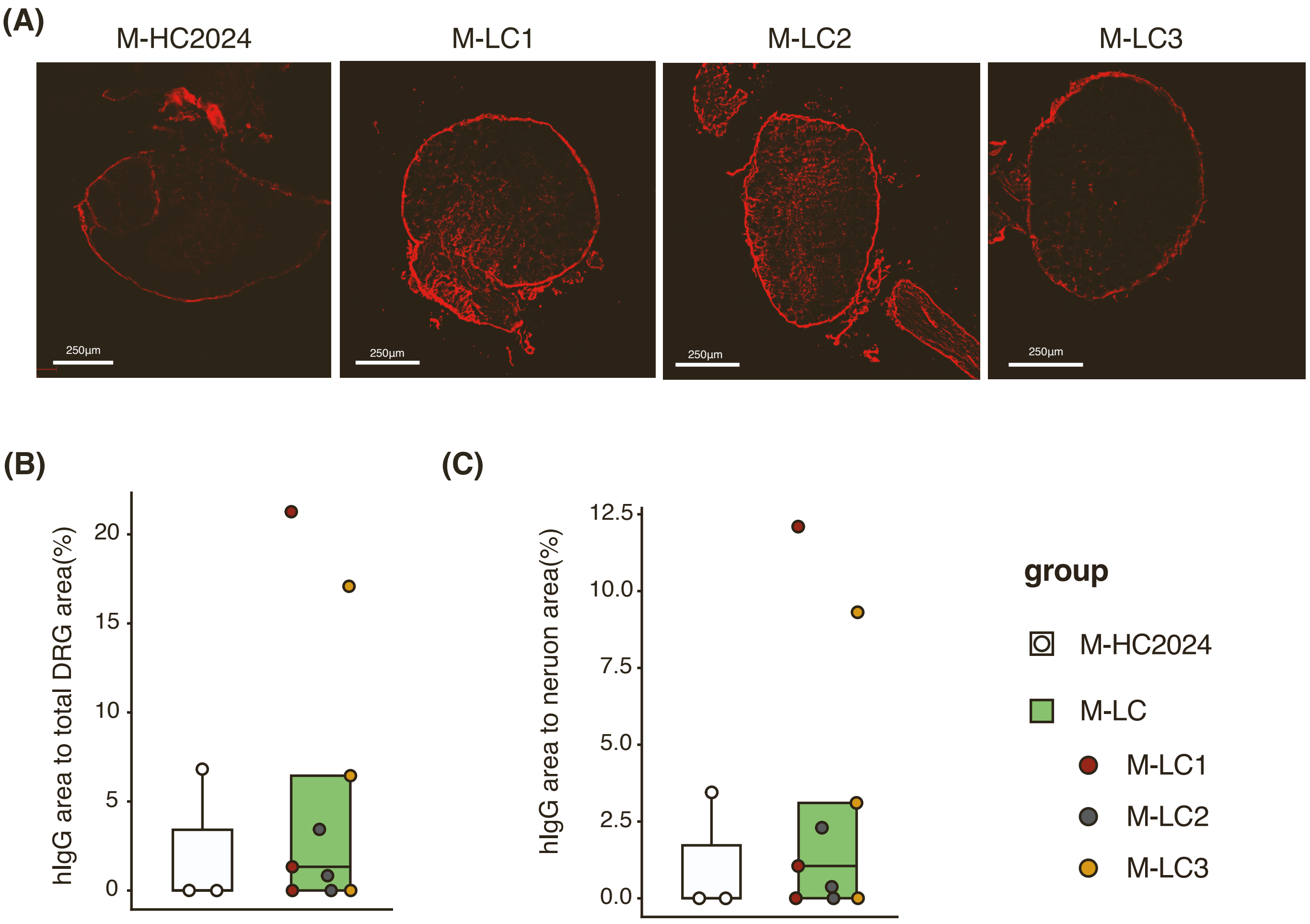

Figure S10: Detection of injected human IgG (hIgG) in murine dorsal root ganglia (DRG) 1-day post-injection. (A) Representative staining of hIgG (red) in DRG from mice injected with pooled IgG from post-SARS-CoV-2-infection healthy controls (M-HC2024) or Long COVID patients (M-LC1-3). Scale bars, 250 μm. (B) Quantification of hIgG-positive area normalized to total DRG area per mouse for M-HC2024 and combined M-LC subgroups. (C) Quantification of hIgG-positive area normalized to total DRG area per mouse across the three Long COVID subgroups (M-LC1, M-LC2, M-LC3) and controls. Boxes show median and IQR. Each dot represents one mouse; colors indicate subgroup: M-HC2024 (white), M-LC1 (red), M-LC2 (gray), M-LC3 (yellow). No significant differences were observed between groups by two-tailed unpaired t-tests.

Supplementary Table 1. overview of cohorts

Cohort	Description	Study	Assays
Long COVID	Patient	AUMC post-COVID-19 Biobank	MSD 2022/2024, proteomics, passive transfer 2022/2024
HC-2022	Post-COVID, non-Long COVID healthy control	S3	MSD 2022
HC-pre	Pre-pandemic healthy control	Sanquin Biobank	passive transfer 2022
HC-2024	Post-COVID, non-Long COVID healthy control	MUSCLE-PASC/ME	MSD 2024, passive transfer 2024

Supplementary Table 2. Baseline characteristics of Long COVID subgroups (2022)

	LC-1<br>N = 12	LC-2<br>N = 10	LC-3<br>N = 12	*P* value
Sex = Male (%)	3 (25.0)	1 (10)	2 (16.7)	0.65
Age (median [IQR])	51 [45, 58]	40 [31, 45]	42 [34, 46]	0.08
Charlson Comorbidity Index (median [IQR])	1 [0, 2]	0 [0, 0]	0 [0, 0]	**0.01**
Time from infection to sampling, days (median [IQR])	232 [160, 316]	330 [274, 375]	231 [190, 381]	0.15
Vaccination = Yes (%)	10 (83)	9 (90)	11 (91.7)	0.80
Working hours prior SARS-CoV-2 (median [IQR])	34 [28, 38]	32 [32, 43]	35 [24, 40]	0.89

Supplementary Table 3. Differentially regulated protein tables OLINK

Supplementary Table 4. Demography of pre-pandemic controls (HC-pre)

	pre-pandemic healthy controls<br>N = 34
Sex = Male (%)	6 (17.6)
Age (median [IQR])	43 [34, 50]

Supplementary Table 5. Normalized IgG autoantibody signal intensities

Intensities are presented as log2-transformed values. Missing values indicate spots in which the foreground fluorescence intensity fell below the local background signal. Protein identifiers are reported as the JHU protein ID and the corresponding NCBI gene symbol.

Supplementary Table 6. Demography of selected Long COVID patients and non-Long COVID healthy controls (2024)

	HC-2024	LC-1 (2024)	LC-2 (2024)	LC-3 (2024)	p	test	Missing
n	7	7	7	5			
sex = Male (%)	3 (42.9)	1 (14.3)	1 (14.3)	1 (20.0)	NaN		0.0
age (median [IQR])	34.00 [26.00, 50.50]	51.00 [51.00, 60.00]	45.00 [32.50, 47.50]	37.00 [34.00, 43.00]	0.104	nonnorm	0.0
BMI (median [IQR])	20.72 [19.90, 22.76]	26.18 [24.39, 30.07]	24.03 [23.51, 25.73]	26.57 [22.84, 31.99]	0.082	nonnorm	0.0
days_since_infection_latest (median [IQR])	678.00 [546.00, 765.00]	499.00 [422.50, 870.50]	863.00 [665.50, 946.00]	336.00 [219.00, 838.00]	0.401	nonnorm	0.0
Working_hours_prepandemic (median [IQR])	40.00 [36.00, 40.00]	34.00 [22.00, 37.00]	36.00 [30.00, 45.00]	35.00 [24.00, 40.00]	0.230	nonnorm	0.0
Working_hours_postpandemic (median [IQR])	40.00 [36.00, 40.00]	7.00 [0.00, 18.00]	0.00 [0.00, 28.00]	0.00 [0.00, 0.00]	0.004	nonnorm	0.0
PEM = Yes (%)	0 (NaN)	6 (85.7)	6 (85.7)	5 (100.0)	NaN		26.9
Charleson_comorbidity_index (median [IQR])	0.00 [0.00, 0.50]	1.00 [0.50, 1.50]	0.00 [0.00, 0.00]	0.00 [0.00, 0.00]	0.029	nonnorm	0.0
SF36_pain (median [IQR])	100.00 [90.00, 100.00]	77.50 [67.50, 83.75]	45.00 [43.75, 67.50]	22.50 [20.00, 67.50]	0.006	nonnorm	0.0
SF36_physical_functioning (median [IQR])	100.00 [97.50, 100.00]	70.00 [60.00, 70.00]	55.00 [45.00, 65.00]	60.00 [10.00, 70.00]	0.002	nonnorm	0.0
SF36_limitations_phys_health (median [IQR])	100.00 [62.50, 100.00]	0.00 [0.00, 0.00]	0.00 [0.00, 0.00]	0.00 [0.00, 0.00]	<0.001	nonnorm	0.0
SF36_limitations_emotional_health (median [IQR])	33.33 [16.67, 66.67]	0.00 [0.00, 33.33]	0.00 [0.00, 50.00]	100.00 [0.00, 100.00]	0.462	nonnorm	0.0
SF36_energy (median [IQR])	85.00 [82.50, 87.50]	50.00 [35.00, 60.00]	45.00 [30.00, 67.50]	25.00 [25.00, 30.00]	0.004	nonnorm	0.0
SF36_emotional_welbeing (median [IQR])	88.00 [76.00, 90.00]	76.00 [64.00, 80.00]	64.00 [56.00, 90.00]	72.00 [64.00, 72.00]	0.357	nonnorm	0.0
SF36_social_functioning (median [IQR])	100.00 [81.25, 100.00]	62.50 [62.50, 68.75]	37.50 [25.00, 81.25]	25.00 [0.00, 50.00]	0.007	nonnorm	0.0
SF36_general_healthy (median [IQR])	90.00 [80.00, 92.50]	40.00 [32.50, 57.50]	40.00 [27.50, 47.50]	25.00 [25.00, 25.00]	0.001	nonnorm	0.0
MFI (median [IQR])	94.00 [90.00, 97.00]	65.00 [57.00, 69.00]	50.00 [44.00, 62.50]	48.00 [40.00, 54.00]	0.001	nonnorm	0.0
EQ5Dvandaag (median [IQR])	85.00 [83.00, 90.00]	72.00 [67.50, 74.50]	54.00 [40.00, 67.00]	25.00 [20.00, 55.00]	0.003	nonnorm	0.0
